# Evaluation of the Aspects of Digital Interventions That Successfully Support Weight Loss: Systematic Review With Component Network Meta-Analysis

**DOI:** 10.2196/65443

**Published:** 2025-05-22

**Authors:** Michael Nunns, Samantha Febrey, Rebecca Abbott, Jill Buckland, Rebecca Whear, Liz Shaw, Alison Bethel, Kate Boddy, Jo Thompson Coon, G.J. Melendez-Torres

**Affiliations:** 1 Isca Evidence, University of Exeter Medical School Faculty of Health & Life Sciences University of Exeter Exeter United Kingdom; 2 NIHR Applied Research Collaboration South West Peninsula (PenARC), University of Exeter Medical School University of Exeter St Lukes Campus Exeter United Kingdom

**Keywords:** systematic review, component network meta-analysis, obesity, digital interventions, digital technology, weight loss, PRISMA

## Abstract

**Background:**

Obesity is a chronic complex disease associated with increased risks of developing several serious and potentially life-threatening conditions. It is a growing global health issue. Pharmacological treatment is an option for patients living with overweight or obesity. Digital technology may be leveraged to support patients with weight loss in the community, but it is unclear which of the multiple digital options are important for success.

**Objective:**

This systematic review and component network meta-analysis aimed to identify components of digital support for weight loss interventions that are most likely to be effective in supporting patients to achieve weight loss goals.

**Methods:**

We searched MEDLINE, Embase, APA PsycInfo, and Cochrane Central Register of Controlled Trials from inception to November 2023 for randomized controlled trials using any weight loss intervention with digital components and assessing weight loss outcomes in adults with BMI ≥25 kg/m^2^ (≥23 kg/m^2^ for Asian populations). Eligible trials were prioritized for synthesis based on intervention relevance and duration, and the target population. Trial arms with substantial face-to-face elements were deprioritized. Prioritized trials were assessed for quality using the Cochrane Risk of Bias Tool v1. We conducted intervention component analysis to identify key digital intervention features and a coding framework. All prioritized trial arms were coded using this framework and were included in component network meta-analysis.

**Results:**

Searches identified 6528 reports, of which 119 were included. After prioritization, 151 trial arms from 68 trials were included in the synthesis. Nine common digital components were identified from the 151 trial arms: provision of information or education, goal setting, provision of feedback, peer support, reminders, challenges or competitions, contact with a specialist, self-monitoring, and incentives or rewards. Of these, 3 components were identified as “best bets” because they were consistently and numerically, but not usually significantly, most likely to be associated with weight loss at 6 and 12 months. These were patient information, contact with a specialist, and incentives or rewards. An exploratory model combining these 3 components was significantly associated with successful weight loss at 6 months (–2.52 kg, 95% CI –4.15 to –0.88) and 12 months (–2.11 kg, 95% CI –4.25 to 0.01). No trial arms used this specific combination of components.

**Conclusions:**

Our findings indicate that the design of digital interventions to support weight loss should be carefully crafted around core components. On their own, no single digital component could be considered essential for success, but a combination of information, specialist contact, and incentives warrants further examination.

**Trial Registration:**

PROSPERO CRD42023493254; https://tinyurl.com/ysyj8j8s

## Introduction

### Background

Obesity is a complex chronic disease associated with increased risks of developing life-limiting and life-threatening conditions, including cardiovascular disease, stroke, and type 2 diabetes [1]. It is a growing global health problem, with the number of obese adults across the world more than doubling since 1990 [2]. This is reflected in the rising prevalence of obesity in the United Kingdom, with 27% of adults in England considered obese in 2017 [3], and this proportion is expected to rise to 35% by 2030 [4].

Weight loss interventions center on lifestyle and behavioral approaches to reduce calorie intake or increase physical activity, pharmacological treatment, and bariatric surgery [5]. Digital technology may be leveraged to support patients with weight loss by providing key motivational features to improve adherence to interventions and sustain weight loss. Digital interventions may offer more coverage, flexibility, and sustainability in service provision and require less resources than face-to-face services.

There is an abundance of literature on the use of digital technology to support weight loss interventions, with several systematic reviews evaluating the effectiveness of interventions using mobile phones (or “mHealth”) or other electronic (or “eHealth”) and digital technologies to lose weight (eg, [6-13]).

This abundant and varied evidence, with equivocal findings, provides both a challenge and an opportunity. The challenge is to make sense of such a heterogeneous body of evidence and identify consistencies between digital approaches and successful outcomes. The volume and variability of trials in this field provide the opportunity for a dataset with which one can establish patterns between intervention components and trial outcomes. In turn, this can inform the development of more effective and efficient digital tools to support weight loss in the community.

### Overall Aim and Objective

The main aim is to identify components of digital support for weight loss interventions that are most likely to be effective in supporting patients to achieve weight loss goals.

The research questions are as follows:

1. What is the breadth and scope of evidence for the effectiveness of weight loss interventions that include digital components?
2. How do we categorize the nature and content of digital components of included interventions?
3. What is the relative effectiveness of different digital intervention components for digitally led weight loss interventions?

## Methods

### Protocol

The protocol for this review was prospectively registered with the International Prospective Register of Systematic Reviews (PROSPERO; ID: CRD42023493254).

### Search Methods

The search strategy was developed by 2 information specialists (JB and AB) in MEDLINE and translated to other databases. The searches used a combination of relevant controlled vocabulary terms (eg, Medical Subject Headings) and free-text terms. Validated search filters for randomized controlled trials (RCTs) were used for searching MEDLINE [14] and Embase [15]. The search strategies for all databases are shown in Multimedia Appendix 1.

### Information Sources

Four bibliographic databases were searched on November 20, 2023: MEDLINE (1946 to current), Embase (1974 to current), APA PsycInfo (1806 to current) via OvidSP, and Cochrane Central Register of Controlled Trials (CENTRAL) (2003 to current) via Wiley Cochrane Library. The databases were searched from inception with no date or language restrictions. Search results were downloaded into EndNote (Version 20; Clarivate Analytics) and deduplicated. Forward and backward citation chasing of prioritized reports was conducted in Scopus (1788 to present) and Citationchaser [16]. The results of citation searching were deduplicated using Systematic Review Accelerator [17], downloaded into EndNote, and deduplicated against the database search results. Finally, a simple search of the terms “trial” and “random” in the title and abstract fields was carried out to identify relevant papers for full-text screening.

### Inclusion and Exclusion Criteria

The below inclusion and exclusion criteria according to the PICO (population, intervention, comparator, and outcome) framework were applied to the identified records.

#### Participants or Population

All participants were required to be adults (18 years or older) with a BMI of ≥25 kg/m^2^ (or ≥23 kg/m^2^ for Asian populations). We excluded trials where participants were recruited from specialist or tertiary settings (eg, hospital or inpatient clinics) or where individuals with specific comorbidities (eg, hypertension and diabetes) were targeted for recruitment.

#### Intervention

Any type of weight loss intervention was eligible, so long as there was a digital component associated with its delivery and it was not delivered as part of secondary or tertiary care weight loss management. Following the definition adopted by Chan et al [18], we defined “digital” intervention components as follows: interventions that are delivered (either in part or full) via an online platform (eg, website, web application, and online forum), a computer or smartphone‐based platform (eg, mobile app, SMS text message–based intervention, and game), or an electronic device of any type. Interventions were required to explicitly target people with obesity or those who were overweight.

#### Comparator or Control

Any comparator was eligible.

#### Outcome

A measure of weight loss, such as absolute or percentage change in body mass or BMI from baseline, was required for inclusion. Outcomes must have been collected at least 6 months (approximately 24 weeks) after baseline assessment.

#### Study Design

Only RCTs and cluster RCTs were eligible for inclusion.

#### Date Limit

There were no restrictions.

### Process for Applying Inclusion Criteria

The title and abstract of each record retrieved by the search were screened by 2 independent reviewers (MN, SF, GJMT, JTC, KB, AB, RA, RW, or LS) to identify records that were clearly irrelevant. The full text of each remaining record was then sought and screened by 2 independent reviewers (MN, SF, GJMT, JTC, KB, AB, RA, RW, LS, or JB) to determine inclusion. Disagreements at each title and abstract stage were not discussed, and instead, full texts were sought. At full-text screening, disagreements were resolved through discussion and group consensus. Articles excluded at the full-text screening stage were coded to indicate the first reason for exclusion.

### Critical Appraisal

Risk of bias was assessed with the Cochrane Risk of Bias Tool v1 [19]. Appraisal was conducted by one reviewer (RA) and checked by a second reviewer (RW), with disagreements resolved through discussion. The results of critical appraisal informed the discussion of evidence.

### Data Extraction

Key information was extracted from included trials by one reviewer and checked by a second reviewer (MN, SF, JB, RA, or GJMT). Discrepancies in extracted data were highlighted and resolved to ensure accuracy. Data were extracted in relation to the following variables to provide an overview of the studies included in the synthesis: study details (author names, title, and date of publication), study aims (stated aim and intervention focus), funding and conflict of interest information, sample characteristics (sample size, age, female percentage, BMI, ethnicity, relevant comorbidities, etc), and outcomes (all categories of outcomes reported).

Sample characteristics relating to age, ethnicity or race, socioeconomic status, and gender or sex were discussed in the context of PROGRESS Plus, a framework used to help draw out the consideration of equity characteristics within research design and analysis [20]. PROGRESS stands for place of residence, race/ethnicity/culture/language, occupation, gender or sex, religion, education, socioeconomic status, and social capital. “Plus” represents other factors associated with discrimination, exclusion, marginalization, or vulnerability, such as personal characteristics, relationships that limit opportunities for health, and environmental situations that provide limited control of opportunities for health. Additional information was extracted to allow description and categorization of the trial arms, as detailed in the synthesis section.

Outcome data were extracted by one reviewer (GJMT) and checked by another (RA). We sought effect sizes (or data to allow their calculation) for weight loss at time points around 6, 12, and 24 months (or longer) from the start of the trial, where available. When multiple estimates of the same effect were provided, we preferred the most complete set of estimates available, extracting imputed estimates over completer-only analyses. We preferred multiple imputation-based estimates over simpler methods but treated multiple imputations using missing not at random models as sensitivity analyses, where these were presented. We preferred model-based estimates of mean differences over simple differences computed from means and SDs, and we preferred model-based estimates of SEs. Where necessary, we estimated SEs of mean differences using the exact *P* values provided. Failing this, we constructed SEs using available data on means, group-specific SEs or SDs, and sample sizes. The only exception to this was in cluster trials with incomplete presentation of outcome data, where we used approximate *P* values to construct SEs for mean differences (eg, [21]).

### Synthesis

The synthesis involved a narrative description of studies, intervention component analysis (ICA), and component network meta-analysis (NMA).

### Prioritization

To optimize the validity of the synthesis, we focused on interventions that were predominantly digital, thereby reducing the number of unknown nondigital variables that would not be coded.

### ICA Process

ICA was conducted to identify key features of interventions that would be carried forward to the component NMA. ICA is an inductive approach that facilitates the identification of critical features of an intervention [22]. ICA is appropriate when dealing with heterogeneous and potentially poorly described interventions, attributing significance to common features without the constraint of “fitting” components to established behavior change taxonomies [22,23].

The ICA process involved the following key stages:

- Two reviewers (MN and SF) selected 10 diverse but well-described interventions from the included papers and independently extracted and categorized key components, using “open coding.”
- The reviewers compared and combined component lists, identifying a tentative set of codes.
- Using axial coding (ie, considering relationships between identified components), the reviewers independently coded the remaining interventions.
- The reviewers met at regular intervals to compare categories, adding or collapsing them as necessary. Further meetings with the review team and members of PERSPEX took place to sense check axial coding and overall categories.
- A final list of intervention component descriptors was produced, using findings from axial coding to organize codes hierarchically (eg, peer support, with axial codes relating to Facebook, chat function, etc).
- All trial arms in the included studies were coded using the intervention component descriptors, with coding checked by a second reviewer (MN or SF).

Intervention component descriptors were tabulated and described with examples. The coded trial arms were taken forward to the component NMA.

### Component NMA

We considered effectiveness on weight loss outcomes using random effects component NMA in a frequentist paradigm, which was implemented using the package *netmeta* in R [24]. We used the component codes (R codes can be found in Multimedia Appendix 2) developed through ICA in an additive model and estimated meta-analyses based on the quality and sufficiency of evidence, focusing on 6-month and 12-month outcomes. We considered heterogeneity over each evidence network but were unable to compare model fit to a standard NMA model due to the complexity of the component scheme. To identify “best bet” components, we examined components that within a given outcome had numerically positive effects at both time points and that had similar patterns of benefits over all included outcomes. Because of the nature of component NMA, we were unable to test for the presence of publication bias.

### Patient and Public Involvement and Engagement

This review benefited from several interactions with PERSPEX, a group of 17 public collaborators who bring their carer, patient, or public perspective to the work of Isca Evidence. PERSPEX members meet monthly online, and membership is culturally, geographically, and demographically diverse [25].

PERSPEX contributed substantially to this review throughout the process. The review topic was first discussed at the August 2023 PERSPEX meeting. Members shared their knowledge of digital support and highlighted digital exclusion and health inequality as areas of concern. Feedback was then sought on the protocol; members could choose to either view a video summary of the draft protocol or read a text-based, plain English version. This led to changes to increase clarity and improve readability. In addition to the protocol summaries, PERSPEX reviewed the search strategy by reading an initial version and making suggestions for additional search terms. PERSPEX provided further input in the early synthesis by reviewing the initial categories identified as part of the ICA (allocated by the review team) to describe the different ways that digital technologies were used within the included studies.

### Ethical Considerations

No ethical approval was required for this project, which exclusively involved secondary analysis of data. To incorporate a diverse range of experiences and views into this work, the research team drew upon the knowledge and expertise of the PERSPEX team throughout the conduct of this review. The PERSPEX team represents individuals living with a range of health conditions, who have different communication preferences. Hence, the research team used a variety of modes of communication to engage with the group, including face-to-face verbal updates and plain-language protocols. During this review, we were not required to handle any personal information.

## Results

### Study Selection

**Figure 1:**
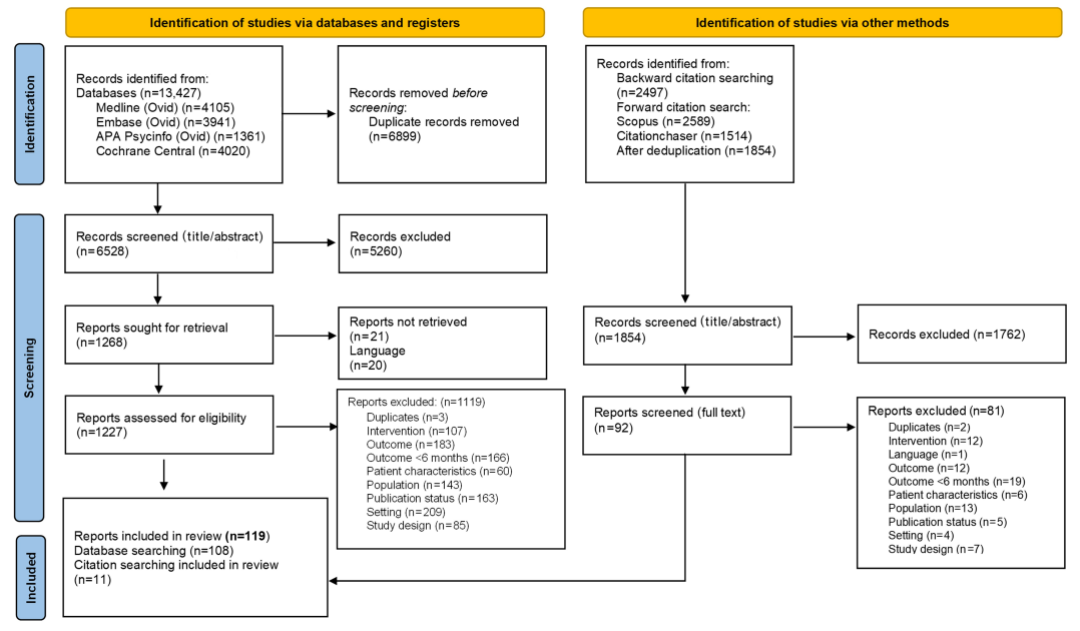
PRISMA (Preferred Reporting Items for Systematic Reviews and Meta-Analyses) flowchart summarizing the results of the literature search and screening for eligibility.

Figure 1 depicts the study selection process and summarizes reasons for exclusion at full-text screening. Bibliographic searches identified 6528 records after deduplication. After title and abstract screening, 1268 (19.4%) references were taken forward and screened at the full-text stage. This led to the exclusion of 1147 reports, with setting (209 reports, 18.2%), outcome (183 reports, 15.9%), no measurements at 24 weeks or beyond (166 reports, 14.4%), and publication status (163 reports, 14.2%) being the most common reasons for ineligibility. Citation chasing identified a further 1854 records after deduplication, of which 92 (4.9%) records were taken forward to full-text screening, and 11 (0.6%) were included in the review. In total, 119 reports from 99 studies were eligible for inclusion. Further prioritization of studies, based on trial arm characteristics, was performed (ICA), and 68 trials were prioritized for full synthesis.

### Sample Characteristics

Multimedia Appendix 3 provides information about the 68 prioritized studies. Of the 68 studies, 36 (53%) were conducted in the United States [21,26-66], 8 (12%) in Australia [67-77], 7 (10%) in the United Kingdom [78-88], 5 (7%) in Germany [89-93], 3 (4%) in Finland [94-96], 2 (3%) in the Netherlands [97-100], and 1 (1%) each in Denmark [101-103], Brazil [104], Switzerland [105], Spain [106], Canada [107], Republic of Korea [108], and Latvia [109].

The interventions evaluated were predominantly aimed at weight loss (52/68, 76%), with others aiming to promote weight maintenance (7/68, 10%) [27,28,36,48,68,77,88,97] or physical activity (1/68, 1%) [107]. Moreover, 8 (12%) studies reported multiple aims, including weight loss and increased physical activity (1/68, 1%) [105], weight loss and disease risk reduction (5/68, 7%) [39,71,72,92,96,109], increased physical activity and disease risk reduction (1/68, 1%) [42], and cost-effectiveness outcomes alongside weight loss (1/68, 1%) [83]. The number of randomized participants per study ranged from 28 [29] to 1386 [98-100]. The mean age of participants ranged from 21.8 years [108] to 58.0 years [61], and the female percentage ranged from 0% (n=7) [38,52,73-77,79,80,107] to 100% (n=10) [26-29,41,42,57,65,66,70,108]. Mean BMI ranged from 28.0 kg/m^2^ [97] to 46.6 kg/m^2^ [29].

Of the 68 studies, 25 (37%) reported no conflicts of interest to disclose [27-32,34,35,37,38,42,45,54,57,59,62,68-76,81-83,96, 105-107,109,110], 8 (12%) did not report this information [26,58,64-66,77,97-100], and 35 (51%) reported some conflicts of interest. Moreover, 67 (99%) studies reported funding sources, with 10 (15%) being funded by an organization directly linked to the diet industry [32-35,59,60,89,108], pharmaceutical industry [56], or other business or commercial sectors [53,56,67,68]. Conversely, 3 (4%) studies did not receive external funding [71,72,101-103,106]. 

### PROGRESS-Plus Data

All 68 prioritized studies reported the age and gender or sex of their study populations, 38 (56%) reported race or ethnicity [21,26-28,30-38,40-65,78-80,83-87], 49 (72%) reported education attainment [21,26-28,30-35,37,38,40-52,55-65,67-70, 73-81,84-89,93-95,97-102,107], 19 (28%) reported employment [21,27,28,30-35,37,38,42,43,51,57,63,69,77-80,86-89,101,102,105], 4 (6%) reported occupation [37,63,73,74,78], 18 (26%) reported income [27,28,30,31,38,41,42,44-46,48,51,57,67,68,70,75-77, 88,107], 5 (7%) reported a deprivation or poverty score [27,28,79-81,86,87], 2 (3%) reported a socioeconomic status score [75-77], and 1 (1%) reported the rural residency of the study population [41]. Of the 68 studies, 16 (24%) specifically targeted a particular gender for recruitment, with 7 (10%) [38,52,73-77,79,80,107] including only men and 9 (13%) [26-29,41,42,57,65,66,70] including only women. Moreover, 1 (1%) had a complete female population, although this was not specified in the inclusion criteria [108]. The inclusion criteria of 3 (4%) studies targeted Black and minority ethnic populations, all of which also targeted women. Furthermore, 1 (1%) study described the intervention as culturally tailored toward African American women [42]. Bennett et al [27] recruited women from health centers with populations that were predominantly racial or ethnic minorities and socially disadvantaged with a low household income [28]. In the study by Steinberg et al [57], recruitment was targeted toward racial or ethnic minority women, and 82% of the recruited population represented non-Hispanic Black women [57]. Additionally, 1 (1%) study recruited women from low-income communities [26]. In the study by Hageman et al [41], recruitment was targeted toward women from rural communities. Of the 7 (10%) studies that targeted men only, 1 specifically recruited men from a rural community [38]. There were no studies targeting older populations specifically. Some studies had an inclusion age of 18 years or older [40,45,64,65,79-82,84,85,88,98-100], while others had an upper age limit restriction, with the oldest limit being 75 years [105] and the youngest limit being 35 years [46].

### Critical Appraisal

**Figure 2:**
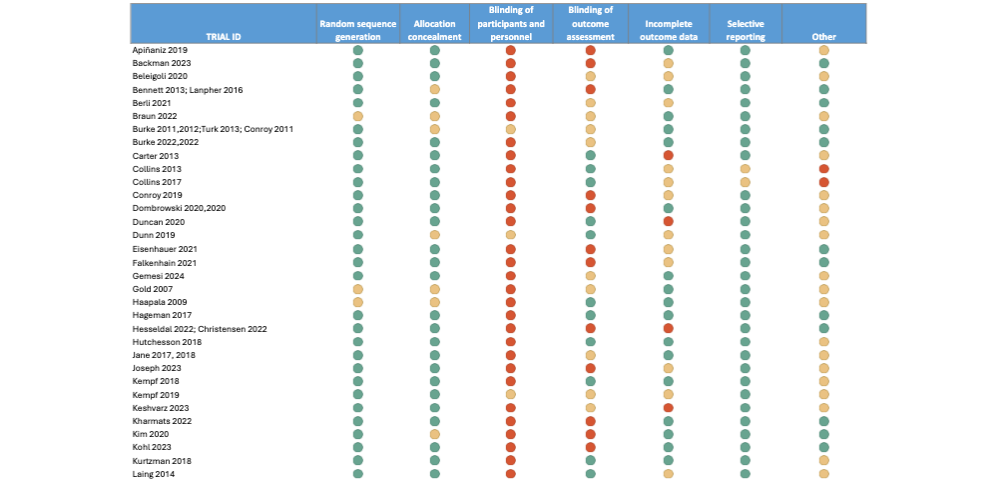
Results of risk of bias appraisal of prioritized studies.

The outcome of critical appraisal of prioritized studies is summarized in Figure 2 [21,26-81,83-92,94-102,104-109]. All studies could not blind participants to their allocation due to the nature of the intervention. Taking this into account, 20 of the 68 (29%) studies were considered to be at relatively low risk of bias (1 area of high risk due to blinding of participants with or without 1 area of unclear reporting) [21,31,41,44,46-48,52, 54,60,63,70,73-77,81,82, 86-88,91,95]. Some concern for risk of bias (multiple areas of unclear reporting with or without 1 area of high risk due to blinding of participants) was identified in 25 (37%) studies [29,32-35,37,40,45,49,50,53,56-59,61,64-66, 71,72,89,90,94,96-100,104,105,109]. Areas that were commonly unclearly reported were allocation concealment (23/68, 34%), blinding of outcome assessment (25/68, 37%), other sources of bias (34/68, 50%), and incomplete outcome data (18/68, 26%). Moreover, 7 (10%) studies did not report their random sequence allocation clearly, and 23 (34%) studies were considered at high risk (more than one area of high risk, including due to blinding). The other areas of high risk identified were blinding of outcome assessment [26-28,36,38,39,42,43,51,55,62,79,80,83-85,92,93, 101,102,106,108], incomplete outcome data [69,78,84,85, 101,102,107], and other forms of bias [67,68].

### ICA Process

#### Development of the Coding Structure

##### Categorizing Interventions

The 99 included trials were highly heterogeneous in terms of the trial arms being compared. Our broad approach to the definition of “digital intervention” meant that interventions that were notionally digital and largely delivered face to face could be included in the ICA, but the nondigital components would not be captured. An example of a notionally digital intervention is the in-person group exercise intervention with email feedback or the “Football Fans In Training” intervention evaluated by Wyke et al [111], which is almost exclusively delivered face to face, with a pedometer used to facilitate activity self-monitoring. The main active ingredients of this intervention would not be captured by our ICA. By contrast, many interventions were fully or primarily digital in their delivery. As our focus was on the effect of digital components of interventions on overall effectiveness, we opted to filter out trial arms that heavily involved face-to-face interactions. We coded trial arms as digital only, digitally led, notionally digital, face-to-face, treatment as usual (TAU), TAU+, or waitlist control, as follows:

- Digital only: Trial arm received content wholly via digital means. This could refer to a single digital component or a combination of digital approaches. No face-to-face delivery of content occurred, but this definition allows for a baseline in-person contact to set-up digital components or in-person assessment visits.
- Digitally led: Trial arm received content primarily via digital means. Nondigital elements, such as an occasional peer-group meeting and face-to-face advice visit, were eligible for inclusion, but these additional nondigital components were supplementary to the main delivery mode of the intervention.
- Notionally digital: Trial arm received content primarily through in-person contact. Digital components might have been involved in content delivery, but these were supplementary to the main approach.
- Face-to-face: Trial arm received only in-person content, either as a group or as individuals.
- TAU (sometimes called usual care): Trial arm may or may not have received limited, often freely available, printed diet, physical activity, or lifestyle information.
- TAU+: Trial arm received nondigital “intervention” information (eg, printed intervention manual). No face-to-face counseling or advice was provided, but the information provided was beyond usual care.
- Waitlist control: Trial arm received nothing other than what it would have normally received.

For inclusion in ICA, studies were required to have trial arms comparing any of the following categories: digital only, digitally led, TAU, TAU+, and waitlist. This approach removed comparisons where our focus on digital component coding would fail to recognize the presence of significant nondigital intervention components. Coding identified 39 of 233 (16.7%) arms as notionally digital and 23 (9.9%) as face-to-face, leaving 68 trials (from 84 papers) and 151 (64.8%) arms for inclusion in the ICA (down from 99 studies and 233 trial arms). Multimedia Appendix 4 lists the trials or arms that were not carried forward because they were notionally digital or primarily face-to-face.

##### Describing Intervention Components

During the multiple rereading of interventions to determine their approach, key characteristics emerged that would form the basis of the ICA coding framework. Despite using an array of digital technologies, all interventions targeted behavior change using similar categories of approaches: provision of information or education, goal setting, provision of feedback, peer support, reminders, challenges or competitions, contact with a specialist, self-monitoring, and incentives or rewards.

These categories were identified from the included trials after initial discussion among reviewers (MN and SF) and subsequent discussion with the wider research team. Categories were discussed with PERSPEX to check sense and to identify categories that might be missing or that could be collapsed. These discussions led to the addition of the “incentives or rewards” category and consideration of whether the intervention was tailored or customized in any way for participants. We also coded whether the intervention more broadly targeted diet, exercise, or both, and whether any face-to-face components were delivered individually or in groups. Table 1 provides a description of digital components, with examples and the frequency of each component among the 151 trial arms.

**Table 1:** Descriptions, examples, and frequencies of digital components of interventions.

Component	Description	Examples	Frequency (N=151), n (%)
Self-monitoring	Participants tracked their own physical activity, dietary intake, or anthropometric measurements (usually weight). This was typically performed using smartphone apps linked to a fitness tracker or by logging in to web-based platforms, but could also be completed using emails or via online consultation.	Self-monitoring smartphone app (Eisenhauer et al [38]; page 3): “received a premium version of a commercially available weight loss app (Lose-It!, FitNow Inc., Boston, MA) that allowed for real-time self-monitoring of eating and activity” Provision of digital tools to facilitate self-monitoring (LaRose et al [46]; page 4): “Participants received digital tools (self-monitoring app and wireless scale) to facilitate self-monitoring” Self-monitoring using a website (Womble et al [66]; page 1013): “[it was recommended to] log on daily to eDiets.com and to record food intake daily during the first 16 weeks”	103 (68.2)
Information or education	Participants received educational information in relation to diet, exercise, weight loss, or general well-being. Most commonly, this was provided via websites, email newsletters, or videos. Participants may receive access to a source of information to sample at their leisure (eg, website or smartphone app) or receive more structured sessions (eg, podcasts, text messages, and webinars).	Website providing educational content (Hutchesson et al [70]; Table number 1, page 3): “Resources provide advice on weight loss, general healthy eating and physical activity, and the 10 Steps to Success” Information delivered by text message (Eisenhauer et al [38]; page 3): “The [intervention] group also received one-way text messages containing content on healthy eating and physical activity” Information provided by a podcast (Dunn et al [37]; page 1527): “Podcasts included behavioral weight-loss techniques based on Social Cognitive Theory and the Diabetes Prevention Program”	99 (65.6)
Feedback	Participants received feedback on any aspect of the intervention or progress toward goals. This could be delivered using a variety of media, with email and text messaging being the most common, and smartphone and web-platform messages were also widely used. Message content was usually in relation to self-monitoring data and could be automated or bespoke.	Feedback via email (Hutchesson et al [70]; Table number 1, page 3): “Participants received automated personalized email feedback from their accredited practising dietitian…focusing on: setting a realistic weight loss goal, their energy requirements for weight loss, and their current eating behaviours and physical activity levels compared to the 10 Steps to Success” Use of email for feedback (LaRose et al [46]; page 4): “Participants received…weekly tailored, written feedback via email on goal progress” Multimodal feedback in response to self-monitoring (Gemesi et al [89]; page 119): “According to the data entered by the users, automated feedback was generated in form of weight trajectory curves, reminders (e.g. to enter the current weight at least once a week), motivating notifications, and interpretations (e.g. which food categories are under- or over-represented in the diet)”	74 (49.0)
Goal setting	Participants set goals for any intervention component or outcome, such as weight loss, physical activity, or diet, over any time period. This was most commonly performed via smartphone apps and web-based platforms. Initial goals were usually set at the beginning of the intervention but could relate to daily, weekly, monthly, or longer time points and require revisiting as necessary.	Goal setting using a web-based platform (Thomas et al [61]; page 589): “The [Weight Watchers] programme focused on the use of a points system to track dietary intake relative to a daily goal in order to produce an energy deficit and increase dietary quality. The daily points goal was personalized based on sex, age, starting weight and activity level” Goal setting using a smartphone app (Gemesi et al [89]; page 119): “participants could set their daily/weekly goals by choosing ones suggested by the app or by choosing self-appointed ones” Website and app providing support for goal setting (Simpson et al [86]; Fig 3, page 13): “Encourage and provide support for goal-setting, action-planning and problem-solving”	72 (47.7)
Reminders	Participants received reminders to complete tasks related to the intervention, such as to log or submit data (self-monitoring), perform exercise, or attend sessions. These may typically be sent through emails, text messages, and notifications from a smartphone app or website.	Use of HTML newsletters sent via email to act as a reminder (Hutchesson et al [70]; Table number 1, page 3): “…reminded participants to complete other program tasks (e.g., quiz, self-monitoring, and goal setting)” Use of a smartphone app to provide reminders (Patel et al [51]; Table number 1, page 3): “In-app automated reminders to track diet and/or weight sent daily” Use of text messaging to provide reminders (Joseph et al [42]; page 5): “Text messages included inspirational quotes, reminders, and tips for how to increase daily [physical activity]”	54 (35.8)
Peer support	Peer support involved contact with other participants, intervention deliverers, and nominated partners or “buddies” and usually occurred via messaging services, such as WhatsApp or SMS text messaging, email, or social media groups like Facebook.	Use of social media to provide peer support (Hutchesson et al [70]; Table number 1, page 3): “Participants joined a private Facebook group and followed a private Instagram account using their personal account” Discussion forum hosted on a website (Hageman et al [41]; Table number 1, page 4): “Discussion board: Peer-led asynchronous discussion with weekly primers posted by peer” Buddy system facilitated by email (Tate et al [59]; page 1621): “A weight loss e-buddy network system that enabled users to match themselves with other persons in the United States with similar characteristics and act as peer support for weight loss via e-mail”	50 (33.1)
Specialist contact	Participants had contact with a trained expert, for example, a dietitian, nutritionist, or psychiatrist. This may have been to discuss progress, revise goals, or provide education. Typically, contact was via email, chat functions, or online meeting platforms such as Zoom.	Email contact from a specialist (Leahey et al [48]; page 4): “Each week participants…emailed the self-monitoring data to their professional coach (a registered dietitian with training in behavioral weight control), they received an email from their coach providing social reinforcement (support, encouragement)” Specialist contact facilitated by chat or video-based digital methods (West et al [65]; page 515): “Groups met online for 1 hour each week in a text‐based or video‐based synchronous chat session facilitated by an experienced behavioural weight control counsellor” Specialist contact via email (Hutchesson et al [70]; Table number 1, page 3): “Participants received automated personalized email feedback from their accredited practising dietitian (APD) focusing on: setting a realistic weight loss goal, their energy requirements for weight loss, and their current eating behaviours and physical activity levels” Semiautomated email contact with primary care physicians (Tate et al [21]; page 5): “In the portal, [primary care providers] could view and edit the default messages for 6 days, after which messages were sent to the patient”	27 (17.9)
Competition or challenge	Competitions, challenges, or games were used within the intervention as extra games or to set short-term goals. Examples include step-based challenges within a smartphone app (eg, Fitbit), hypothetical scenarios, or challenges involving logging food intake for 7 days in a row. Participants may compete with others or by themselves.	Challenge within a web-based system (Thomas et al [61]; page 591): “The first mini-game asked participants to plan ahead and problem solve to stay under a calorie goal while ordering food from a takeout menu or cooking at home” Challenge provided through an app (Kim et al [108]; page 5): “Weekly group missions were provided to the digital CBT group based on the expectation that social supports (eg, communicating needs and building positive support) would intensify the motivation”	15 (10.0)
Incentive or reward	Digital incentives or rewards were offered either as an explicit component of the intervention or in relation to progress toward goals (eg, virtual “badges” for achievements). Incentives could be facilitated using digital means, for example, a digital “bank,” or could be linked to peer support and challenges (social incentives).	Virtual reward (Tate et al [21]; page 17): “Progress page where participants could view graphs of their weight, diet, and exercise, view progress toward their goals, and receive virtual badges for achieving weight loss milestones” Virtual reward (Simpson et al [86]; Table number 46, page 137): “In-app reward of medals/trophies for regular login and progress” Virtual bank (Leahey et al [47]; page 4): “If participants completed all reporting, they earned a maximum of $45 during the entire program. The SII website included a “bank” which displayed the participant’s previous week’s earnings and total earnings” Social incentive (Kurtzman et al [44]; page 1670): “every Monday, the team was endowed with 70 points… Each day, the team was informed of the one member who was selected at random to represent their team. If that member weighed in on the prior day… the team kept its points; otherwise, 10 points were lost” Social reward (Leahey et al [48]; page 4): “Each week participants self-monitored their weight and the prescribed diet or activity behavior for at least 5 days, and emailed the self-monitoring data to their professional coach…they received an email from their coach providing social reinforcement”	10 (6.6)

### ICA Findings

Multimedia Appendix 5 shows how the 151 trial arms were coded. Of the 151 trial arms, 90 (59.6%) were digital only, 28 (18.5%) were digitally led, 12 (7.9%) were TAU+, 10 (6.6%) were TAU, and 11 (7.3%) were waitlist. Most arms (130/151, 86.1%) targeted diet and exercise, with 7 (4.6%) targeting diet alone [37,39,81,82,108] and 5 (3.3%) focusing on physical activity alone [42,105,107]. In 3 (1.9%) trial arms from the same study, the intervention targets were unclear [79,80], and in 1 (0.6%), the focus was on general health and well-being [42]. While all prioritized studies included a weight loss outcome at 6 months or beyond, intervention durations ranged from 14 days [105] to 24 months [32,34-36,104,110]. The median intervention duration was 6 months (mean 8.4, SD 5.4 months). There were 8 (12%) trials with more than two included arms [41,48,51,71,72,78-80,90,96].

A tenet of all interventions was to achieve a certain amount of weight loss, reduce calories by a certain amount, or increase activity to a certain level, and as such, all included goal setting to some extent, whether as a social process or an intervention activity, and it was made explicit or facilitated with digital technologies. We coded digitally facilitated goal setting as an intervention exercise. Websites and web-based platforms were the most common approaches for this, featuring in 31 of 151 (20.5%) trial arms. Applications, predominantly for use with a smartphone, were adopted in 24 (15.9%) trial arms. Goal setting was often conducted during initial consultation and orientation, where this occurred, and therefore, it could have been completed in-person. Goal setting and revision, and checking against progress could be completed quickly and easily using a digital platform. For example, in the study by Kurtzman et al [44], baseline weight was entered and a target based on 6% or 8% weight loss was provided. Smartphone apps offer similar and arguably more convenient functionality, with Eisenhauer et al [38] demonstrating that they facilitated the engagement of men from rural communities in a weight loss program owing to their ease of use with respect to goal setting (and provision of real-time feedback).

The use of other technologies was often linked with achieving goals. For example, reminders were often in relation to meeting targets for diet or exercise, or to completing self-monitoring, which itself was usually conducted to measure progress against goals. Incentives were provided, and competitions or challenges were designed with goals in mind. Feedback frequently focused on progress in relation to goals. Only the provision of broader information and educational content was less explicitly about achieving goals, although it was still linked to the overall goal of achieving weight loss.

The linked nature of components was emphasized by the fact that only 7 of 151 (4.6%) intervention arms (not counting trial arms designated as comparators by study authors) from 5 (7%) studies [71,78,90,91,109] were coded as having a single digital component. This was unsurprising because of the abundant literature highlighting the importance of multiple behavior change techniques in weight loss interventions. Furthermore, in many cases, a single digital device or technology was able to provide multiple components of the intervention. For example, smartphone apps facilitated three or more components in 18 (26%) trials [37-39,42,44,45,51,60,61,69,72,78,86, 87,89,92,93,95,101,102,108], and this was the case in 17 (25%) trials using websites [21,41,47,49,50,52,59-61,64,65,70,73,79, 80,83,96,97,101,102,112] and in 7 (10%) trials using web-based platforms [36,40,61,66-68,104].

### Component NMA

We estimated component NMA models for absolute weight loss, absolute change in BMI, weight loss expressed as a percentage of baseline weight, and responders (defined as the proportion achieving more than 5% weight loss from baseline). Model diagnostics are presented in Multimedia Appendix 6. Meta-analyses varied in size from 43 trials for absolute weight loss at 6 months to 14 trials for absolute change in BMI at 12 months. Across the main analyses, heterogeneity was as expected, ranging from an *I*^2^ of 30.1% for change in BMI at 12 months to 61.1% for absolute weight loss at 6 months. Analyses for weight loss expressed as a percentage of baseline weight are presented as exploratory given the high levels of heterogeneity relative to the richness of the data structure. All extracted effect sizes can be found in Multimedia Appendix 7.

Because of the data structures of each evidence network, we were unable to compare model fit to standard combination-based NMA models, meaning we were unable to formally assess for the presence of inconsistency. However, heterogeneity estimates in every case were lower than those from pairwise meta-analyses of interventions against TAU, indicating that inconsistency would be unlikely to threaten the validity of our analyses. Goal setting could only be evaluated as a component at 12 months for the absolute weight loss outcome given its co-occurrence with other components in our meta-analyses.

**Figure 3:**
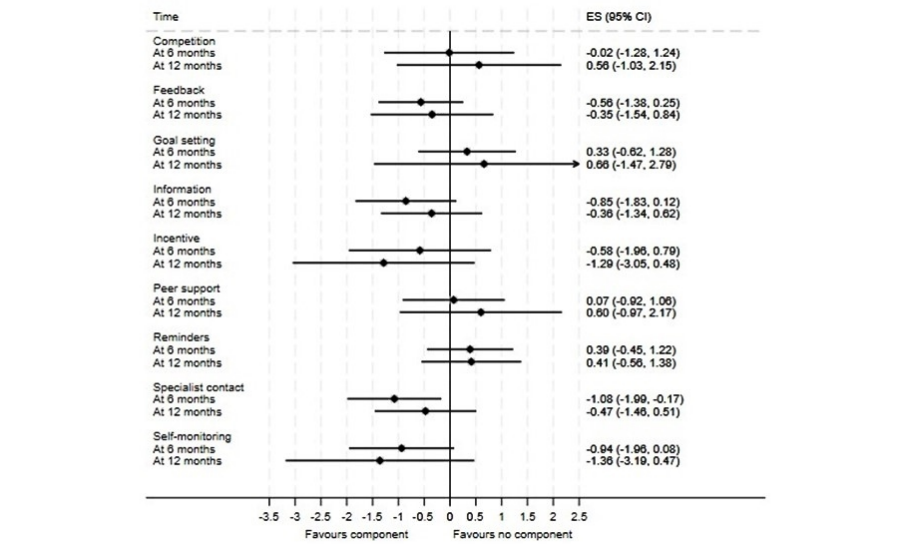
Component network meta-analysis of digital components of interventions for absolute weight loss at 6 and 12 months. ES: effect size.

**Figure 4:**
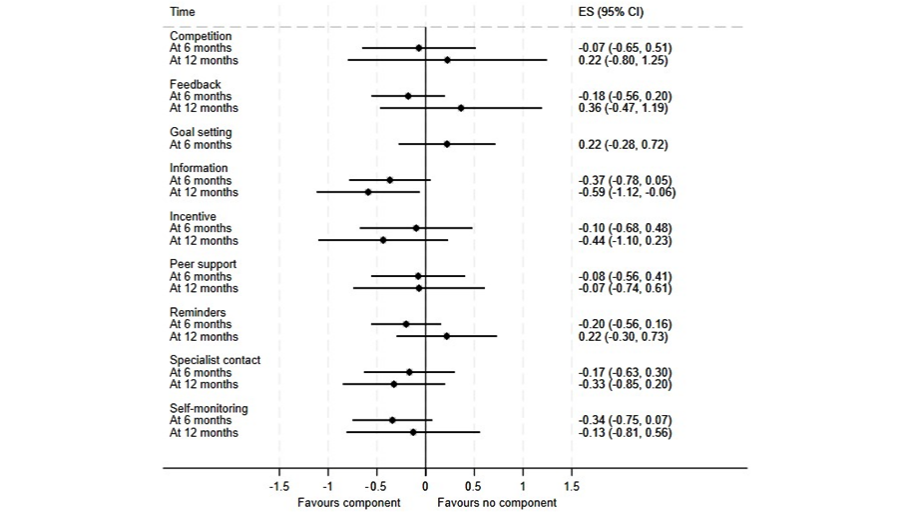
Component network meta-analysis of digital components of interventions for change in BMI at 6 and 12 months. ES: effect size.

**Figure 5:**
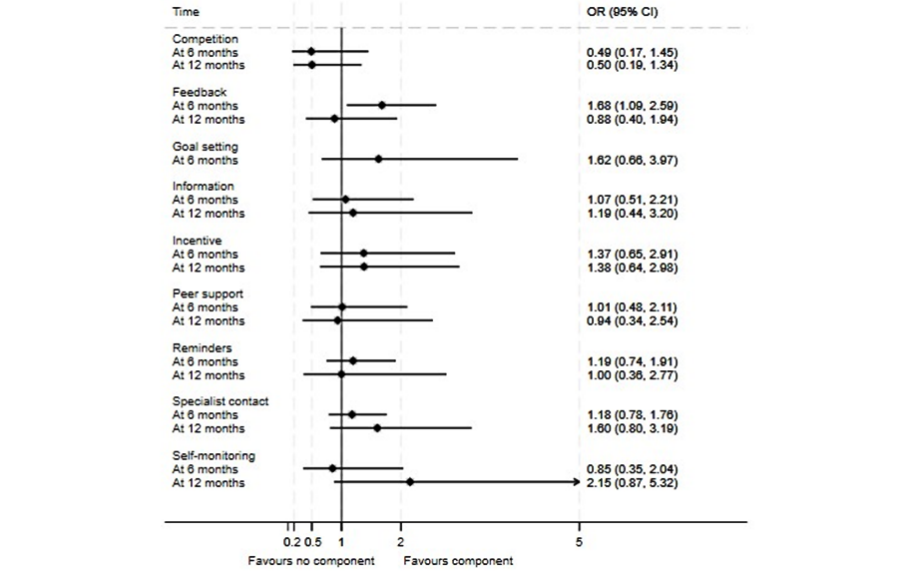
Component network meta-analysis of digital components of interventions for participants achieving 5% weight loss at 6 and 12 months. OR: odds ratio.

Findings from meta-analyses are presented in Figures 3-5 (see Multimedia Appendix 8 for meta-analyses of weight loss as a percentage of baseline weight). In analyses for absolute weight loss (Figure 3), the only component linked to a statistically significant effect was specialist contact, which was associated with weight loss of 1.08 kg (95% CI 0.17-1.99) at 6 months. A similar effect was in the evidence for this component in weight loss as a percentage of baseline weight (–2.21%, 95% CI –3.64 to –0.78; Multimedia Appendix 8). In analyses for BMI (Figure 4), the only component linked to a statistically significant effect was information, which was linked to a BMI loss of 0.59 (95% CI 0.06-1.12) at 6 months. Feedback was the only component linked to a significant increase in the odds of achieving at least 5% weight loss (odds ratio 1.68, 95% CI 1.09-2.59) at 6 months (Figure 5), but the estimate of the effect at 12 months suggested some numerical, but not statistical, evidence of harm.

Reading across outcomes, 3 components emerged as the best bets: information, incentives or rewards, and specialist contact. Each of these components had numerically beneficial effect estimates in every meta-analysis model. An exploratory random effects meta-analysis modeling the potential effectiveness of an intervention comprising these 3 components alone suggested significant short-term impacts on absolute weight loss (–2.52 kg, 95% CI –4.15 to –0.88), indicative long-term impacts on this outcome (–2.11 kg, 95% CI –4.25 to 0.01), and significant effects for BMI loss at 12 months (–1.35, 95% CI –2.40 to –0.30) but not 6 months (–0.63, 95% CI –1.38 to 0.11). However, no trial arms used this specific combination of components.

## Discussion

### Principal Findings

This review is the first to investigate which digital components of weight loss interventions are associated with successful weight loss. After deprioritizing trials not evaluating predominantly digital interventions, we focused on 68 trials, roughly equally categorized as those that had low risk of bias, those that had some concern over risk of bias, and those that had high risk of bias. We performed ICA on 151 trial arms from the 68 studies with data from over 14,500 participants to identify the key digital components of interventions. We then conducted component NMA to ascertain which of the components were associated with successful outcomes.

The ICA identified 9 components facilitated by digital technology: goal setting, self-monitoring, peer support, reminders, feedback, specialist contact, information or education, competitions or challenges, and incentives or rewards. These components were delivered or facilitated by a range of digital technologies, with websites, web-based platforms, apps (ie, for smartphones), SMS text messages, and emails being the most common.

The component NMA showed that no single digital component stood out as being singularly linked to effectiveness at 6-month or 12-month time points, but there were 3 “best bet” components that were consistently associated with favorable, but not usually statistically significant, point estimates across outcomes. These were provision of information or education, specialist contact, and incentives or rewards. An exploratory analysis modeling the potential effectiveness of an intervention comprising these 3 components alone suggested significant 6-month impacts and indicative 12-month impacts on absolute weight loss, with a significant reduction in BMI at 12 months.

It is unsurprising that a single digital component did not stand out as being crucial for weight loss interventions. All 9 components have appropriate underpinnings from behavioral science that would suggest their effectiveness, but there are also reasons why they (or their digital versions) may have limited impacts. Goal setting is likely a valuable approach for weight loss. For example, Melendez-Torres et al [113] identified directive, provider-led goal setting as an important component of interventions. However, our null findings suggest that a more self-directed approach inherent with digital approaches does not mimic this, but perhaps an approach involving goal setting by or in consultation with a specialist might mimic this. A mix of complementary components is likely to be the most successful approach.

Our exploratory analysis suggests that a combined approach involving digitally facilitated information, specialist contact, and incentives or rewards may be linked with successful weight loss, though we note that none of our included trial arms used this specific 3-component approach, and thus, we are unable to further validate this proposal with direct evidence.

Websites and web-based platforms, smartphone apps, newsletters, and emails were the most common sources of information provision, and it is logical to conclude that provision of high-quality, on-demand information is beneficial for patients.

Components that employ regular or repetitive participant interaction, such as reminders, automated feedback, and self-monitoring, are likely to fade in effectiveness over time, even when habit formation has occurred. The median intervention duration in this review was comfortably beyond the proposed 66 days suggested to be linked with habit formation [114]. However, motivation and behavior change habits are typically short-lived [115]. Perhaps relatedly, weight loss interventions targeting behavior change frequently see a peak in effectiveness around 6 months, followed by a tendency for weight regain [116].

Our review suggests that interventions combining the 3 digital components of information, specialist contact, and incentives or rewards may lead to weight loss benefits, but trials are needed. We provide examples of these elements from included trials. The nature (eg, intensity, mode of delivery, timing, etc) of digital interventions combining key components should be investigated. In general, there is a need for more trials of digital interventions to capture longer-term outcome data (beyond 24 months).

### Limitations

Although we refined the evidence to include interventions that were wholly or predominantly digitally delivered, we were unable to account for other factors that may have influenced outcomes, such as nondigital intervention versions of the 9 coded components and patient characteristics like age and comorbidities. Our refined approach also led to the elimination of 82 trial arms that were only notionally digital or compared with face-to-face comparators, reducing the data available for synthesis.

The component NMA used coding that captured the presence of a component but not its nature (ie, frequency, intensity, etc). The examples we highlight for each “best bet” component demonstrate variations in delivery, and it can be expected that different modes of delivery will yield variations in effectiveness. As such, further investigation is required to determine the optimal components of future interventions, which may vary between individuals.

Many of the included trials imposed an upper BMI limit of 39.9 kg/m^2^ for inclusion into the study (upper range for “obese” patients). Therefore, the needs of patients with severe obesity are not well represented in this review.

We focused on weight loss outcomes, and it was beyond our scope to consider other important health-related or process-relevant outcomes, such as effects on well-being and mental health, or consider the implementation of, engagement with, or adherence to interventions. As such, it must be acknowledged that this work addresses a small part of the weight loss conundrum.

### Comparison With Prior Work

Digital self-monitoring, which is one of the intervention components where leveraging technology has the most obvious advantage over analog approaches, was shown in a systematic review to be associated with weight loss [117], but it can have greater benefits when part of a broader weight loss intervention involving specialist contact [118]. Contact with a specialist has previously been shown to complement and enhance the power of other intervention components, such as self-monitoring and goal setting [113,118]. While the provision of contact with specialists has resource implications for weight loss interventions, our findings suggest that digital technology can be leveraged to help facilitate contact between patients and specialists. Examples from trials in this analysis include contact through videoconferencing, removing the need for travel (eg, [26,29]), implementing an embedded forum with contact initiated from either side [104], and using the electronic health record portal to allow primary care practitioners to review progress and provide feedback [36].

Weight regain is a complex and multifaceted phenomenon, influenced in part by behavioral factors (eg, noncompliance, lack of motivation, and waning of newly formed habits) and by the interaction of hormonal and metabolic factors [119]. As such, behavior change interventions may be expected to have limited long-term effectiveness. Weight loss that is maintained for 2 years or longer is likely to be sustained for several years [120,121], but we did not have enough data at 24 months or beyond to be able to explore digital intervention components associated with weight loss at this time point.

### Conclusions

Overall, this is the first systematic review exploring which digital components of weight loss interventions are associated with successful weight loss outcomes, using a novel approach combining ICA and component NMA based on 68 trials. Our findings suggest that there are 9 widely used digital approaches in weight loss interventions: goal setting, feedback, self-monitoring, information or education, reminders, contact with a specialist, peer support, competitions or challenges, and incentives or rewards. No single component stands alone as being crucial to intervention success, but there are 3 “best bets” that have effect estimates numerically, but not statistically, linked with successful outcomes. These are information or education, specialist contact, and incentives or rewards. Exploratory modeling suggests that a digital intervention combining these 3 components may be associated with successful weight loss or BMI reduction at 6 and 12 months.

Our findings, drawn from a sizeable body of weight loss RCTs, indicate no consistent association between components of digital support and weight loss outcomes. This report provides directions for future research in an effort to establish more reliable support. We suggest that further evidence is needed to develop simplified digital interventions to support people with weight loss. These should provide high-quality digital information, should facilitate contact with a specialist (eg, a dietitian), and may include incentives or rewards for progress and intervention compliance. There is a need for such trials to have longer-term follow-up that extends beyond 2 years.

## Appendix

Multimedia Appendix 1 Search strategies for all databases.

Multimedia Appendix 2 R Codes for component network meta-analysis.

Multimedia Appendix 3 Sample characteristics.

Multimedia Appendix 4 Trials or trial arms not carried forward to full synthesis.

Multimedia Appendix 5 Intervention coding table.

Multimedia Appendix 6 Component network meta-analysis model diagnostics.

Multimedia Appendix 7 Extracted effect sizes for all trial arms included in the component network meta-analysis.

Multimedia Appendix 8 Results of component network meta-analysis for percentage weight loss.

Multimedia Appendix 9 PRISMA (Preferred Reporting Items for Systematic Reviews and Meta-Analyses) checklist.

## Abbreviations

ICA: intervention component analysis
NMA: network meta-analysis
RCT: randomized controlled trial
TAU: treatment as usual

## References

[1] (2016). Managing obesity in men. National Institute for Health and Care Research (NIHR).

[2] Obesity and overweight. World Health Organization (WHO).

[3] (2017). Statistics on Obesity, Physical Activity and Diet - England. NHS Digital.

[4] Obesity Update 2017. The Organisation for Economic Co-operation and Development (OECD).

[5] (2023). Obesity: identification, assessment and management. National Institute for Health and Care Excellence.

[6] Aguilar-Martínez A, Solé-Sedeño JM, Mancebo-Moreno G, Medina FX, Carreras-Collado R, Saigí-Rubió F (2014). Use of mobile phones as a tool for weight loss: a systematic review. J Telemed Telecare.

[7] Cavero-Redondo I, Martinez-Vizcaino V, Fernandez-Rodriguez R, Saz-Lara A, Pascual-Morena C, Álvarez-Bueno C (2020). Effect of behavioral weight management interventions using lifestyle mHealth self-monitoring on weight loss: a systematic review and meta-analysis. Nutrients.

[8] Dounavi K, Tsoumani O (2019). Mobile health applications in weight management: a systematic literature review. Am J Prev Med.

[9] O'Boyle J, Davidson P (2022). The effects of mHealth versus eHealth on weight loss in adults: a systematic review. Topics in Clinical Nutrition.

[10] Podina IR, Fodor LA (2018). Critical review and meta-analysis of multicomponent behavioral e-health interventions for weight loss. Health Psychol.

[11] Ryan K, Dockray S, Linehan C (2019). A systematic review of tailored eHealth interventions for weight loss. Digit Health.

[12] Wang E, Abrahamson K, Liu PJ, Ahmed A (2020). Can mobile technology improve weight loss in overweight adults? a systematic review. West J Nurs Res.

[13] Willmott TJ, Pang B, Rundle-Thiele S, Badejo A (2019). Weight management in young adults: systematic review of electronic health intervention components and outcomes. J Med Internet Res.

[14] Cochrane Handbook for Systematic Reviews of Interventions Version 6.4. The Cochrane Collaboration.

[15] Glanville J, Foxlee R, Wisniewski S, Noel-Storr A, Edwards M, Dooley G (2019). Translating the Cochrane EMBASE RCT filter from the Ovid interface to Embase.com: a case study. Health Info Libr J.

[16] Haddaway NR, Grainger MJ, Gray CT (2021). Citationchaser: an R package and shiny app for forward and backward citations chasing in academic searching. Zenodo.

[17] The Systematic Review Accelerator (SRA). Institute for Evidence-Based Healthcare.

[18] Chan A, De Simoni A, Wileman V, Holliday L, Newby C, Chisari C, Ali S, Zhu N, Padakanti P, Pinprachanan V, Ting V, Griffiths CJ (2022). Digital interventions to improve adherence to maintenance medication in asthma. Cochrane Database Syst Rev.

[19] Cochrane Handbook for Systematic Reviews of Interventions Version 5.2. The Cochrane Collaboration.

[20] PROGRESS-Plus. Cochrane.

[21] Tate DF, Kraschnewski JL, Martinez C, Diamond M, Veldheer S, Hwang KO, Lehman EB, Yang C, Sciamanna CN (2022). A cluster-randomized controlled trial of automated internet weight-loss programs in primary care: Role of automated provider feedback. Obesity (Silver Spring).

[22] Sutcliffe K, Thomas J, Stokes G, Hinds K, Bangpan M (2015). Intervention Component Analysis (ICA): a pragmatic approach for identifying the critical features of complex interventions. Syst Rev.

[23] Rizzo AJ, Orr N, Shaw N, Farmer C, Chollet A, Young H, Berry V, Rigby E, Hagell A, Bonell C, Melendez-Torres GJ (2023). Exploring the activities and target audiences of school-based violence prevention programs: systematic review and intervention component analysis. Trauma Violence Abuse.

[24] Balduzzi S, Rücker G, Nikolakopoulou A, Papakonstantinou T, Salanti G, Efthimiou O, Schwarzer G (2023). netmeta: an R package for network meta-analysis using frequentist methods. J Stat Soft.

[25] PERSPEX: Public Engagement in Research for health and Social Policy at Exeter. University of Exeter.

[26] Backman DR, Kohatsu ND, Padovani AJ, Dao C, Ritley D, Fleuret JE, McCracken CR (2022). Achieving weight loss through a community-based, telewellness programme: A randomised controlled trial. Health Education Journal.

[27] Bennett GG, Foley P, Levine E, Whiteley J, Askew S, Steinberg DM, Batch B, Greaney ML, Miranda H, Wroth TH, Holder MG, Emmons KM, Puleo E (2013). Behavioral treatment for weight gain prevention among black women in primary care practice: a randomized clinical trial. JAMA Intern Med.

[28] Lanpher MG, Askew S, Bennett GG (2016). Health literacy and weight change in a digital health intervention for women: a randomized controlled trial in primary care practice. J Health Commun.

[29] Braun TD, Olson K, Panza E, Lillis J, Schumacher L, Abrantes AM, Kunicki Z, Unick JL (2022). Internalized weight stigma in women with class III obesity: A randomized controlled trial of a virtual lifestyle modification intervention followed by a mindful self-compassion intervention. Obes Sci Pract.

[30] Burke LE, Sereika SM, Bizhanova Z, Parmanto B, Kariuki J, Cheng J, Beatrice B, Cedillo M, Pulantara IW, Wang Y, Loar I, Conroy MB (2022). The effect of tailored, daily, smartphone feedback to lifestyle self-monitoring on weight loss at 12 months: the SMARTER randomized clinical trial. J Med Internet Res.

[31] Burke LE, Sereika SM, Parmanto B, Bizhanova Z, Kariuki JK, Cheng J, Beatrice B, Loar I, Pulantara IW, Wang Y, Cedillo M, Conroy MB (2022). Effect of tailored, daily feedback with lifestyle self-monitoring on weight loss: The SMARTER randomized clinical trial. Obesity (Silver Spring).

[32] Burke LE, Conroy MB, Sereika SM, Elci OU, Styn MA, Acharya SD, Sevick MA, Ewing LJ, Glanz K (2011). The effect of electronic self-monitoring on weight loss and dietary intake: a randomized behavioral weight loss trial. Obesity (Silver Spring).

[33] Burke LE, Styn MA, Sereika SM, Conroy MB, Ye L, Glanz K, Sevick MA, Ewing LJ (2012). Using mHealth technology to enhance self-monitoring for weight loss: a randomized trial. Am J Prev Med.

[34] Turk MW, Elci OU, Wang J, Sereika SM, Ewing LJ, Acharya SD, Glanz K, Burke LE (2013). Self-monitoring as a mediator of weight loss in the SMART randomized clinical trial. Int J Behav Med.

[35] Conroy M, Yang K, Elci O, Gabriel K, Styn M, Wang J, Kriska AM, Sereika SM, Burke LE (2011). Physical activity self-monitoring and weight loss: 6-month results of the SMART trial. Med Sci Sports Exerc.

[36] Conroy MB, McTigue KM, Bryce CL, Tudorascu D, Gibbs BB, Arnold J, Comer D, Hess R, Huber K, Simkin-Silverman LR, Fischer GS (2019). Effect of electronic health record–based coaching on weight maintenance. Ann Intern Med.

[37] Dunn CG, Turner-McGrievy GM, Wilcox S, Hutto B (2019). Dietary self-monitoring through calorie tracking but not through a digital photography app is associated with significant weight loss: the 2SMART pilot study-a 6-month randomized trial. J Acad Nutr Diet.

[38] Eisenhauer CM, Brito F, Kupzyk K, Yoder A, Almeida F, Beller RJ, Miller J, Hageman PA (2021). Mobile health assisted self-monitoring is acceptable for supporting weight loss in rural men: a pragmatic randomized controlled feasibility trial. BMC Public Health.

[39] Falkenhain K, Locke SR, Lowe DA, Reitsma NJ, Lee T, Singer J, Weiss EJ, Little JP (2021). Keyto app and device versus WW app on weight loss and metabolic risk in adults with overweight or obesity: A randomized trial. Obesity (Silver Spring).

[40] Gold BC, Burke S, Pintauro S, Buzzell P, Harvey-Berino J (2007). Weight loss on the web: A pilot study comparing a structured behavioral intervention to a commercial program. Obesity (Silver Spring).

[41] Hageman P, Pullen C, Hertzog M, Pozehl B, Eisenhauer C, Boeckner L (2017). Web-based interventions alone or supplemented with peer-led support or professional email counseling for weight loss and weight maintenance in women from rural communities: results of a clinical trial. J Obes.

[42] Joseph RP, Todd M, Ainsworth BE, Vega-López S, Adams MA, Hollingshead K, Hooker SP, Gaesser GA, Keller C (2023). Smart Walk: A culturally tailored smartphone-delivered physical activity intervention for cardiometabolic risk reduction among African American women. Int J Environ Res Public Health.

[43] Kharmats AY, Wang C, Fuentes L, Hu L, Kline T, Welding K, Cheskin LJ (2022). Monday-focused tailored rapid interactive mobile messaging for weight management 2 (MTRIMM2): results from a randomized controlled trial. Mhealth.

[44] Kurtzman GW, Day SC, Small DS, Lynch M, Zhu J, Wang W, Rareshide CAL, Patel MS (2018). Social incentives and gamification to promote weight loss: the LOSE IT randomized, controlled trial. J Gen Intern Med.

[45] Laing BY, Mangione CM, Tseng C, Leng M, Vaisberg E, Mahida M, Bholat M, Glazier E, Morisky DE, Bell DS (2014). Effectiveness of a smartphone application for weight loss compared with usual care in overweight primary care patients. Ann Intern Med.

[46] LaRose JG, Leahey TM, Lanoye A, Bean MK, Fava JL, Tate DF, Evans RK, Wickham EP, Henderson MM (2022). Effect of a lifestyle intervention on cardiometabolic health among emerging adults: a randomized clinical trial. JAMA Netw Open.

[47] Leahey TM, Subak LL, Fava J, Schembri M, Thomas G, Xu X, Krupel K, Kent K, Boguszewski K, Kumar R, Weinberg B, Wing R (2015). Benefits of adding small financial incentives or optional group meetings to a web-based statewide obesity initiative. Obesity (Silver Spring).

[48] Leahey TM, Fava JL, Seiden A, Fernandes D, Doyle C, Kent K, La Rue M, Mitchell M, Wing RR (2016). A randomized controlled trial testing an Internet delivered cost-benefit approach to weight loss maintenance. Prev Med.

[49] Olson R, Wipfli B, Thompson SV, Elliot DL, Anger WK, Bodner T, Hammer LB, Perrin NA (2016). Weight control intervention for truck drivers: the SHIFT randomized controlled trial, United States. Am J Public Health.

[50] Wipfli B, Hanson G, Anger K, Elliot DL, Bodner T, Stevens V, Olson R (2019). Process evaluation of a mobile weight loss intervention for truck drivers. Saf Health Work.

[51] Patel ML, Hopkins CM, Brooks TL, Bennett GG (2019). Comparing self-monitoring strategies for weight loss in a smartphone app: randomized controlled trial. JMIR Mhealth Uhealth.

[52] Patrick K, Calfas KJ, Norman GJ, Rosenberg D, Zabinski MF, Sallis JF, Rock CL, Dillon LW (2011). Outcomes of a 12-month web-based intervention for overweight and obese men. Ann Behav Med.

[53] Rogers RJ, Lang W, Barone Gibbs B, Davis KK, Burke LE, Kovacs SJ, Portzer LA, Jakicic JM (2016). Applying a technology-based system for weight loss in adults with obesity. Obes Sci Pract.

[54] Ross KM, Wing RR (2016). Impact of newer self-monitoring technology and brief phone-based intervention on weight loss: A randomized pilot study. Obesity (Silver Spring).

[55] Shapiro JR, Koro T, Doran N, Thompson S, Sallis JF, Calfas K, Patrick K (2012). Text4Diet: a randomized controlled study using text messaging for weight loss behaviors. Prev Med.

[56] Shuger SL, Barry VW, Sui X, McClain A, Hand GA, Wilcox S, Meriwether RA, Hardin JW, Blair SN (2011). Electronic feedback in a diet- and physical activity-based lifestyle intervention for weight loss: a randomized controlled trial. Int J Behav Nutr Phys Act.

[57] Steinberg DM, Levine EL, Askew S, Foley P, Bennett GG (2013). Daily text messaging for weight control among racial and ethnic minority women: randomized controlled pilot study. J Med Internet Res.

[58] Tate DF, Wing RR, Winett RA (2001). Using Internet technology to deliver a behavioral weight loss program. JAMA.

[59] Tate DF, Jackvony EH, Wing RR (2006). A randomized trial comparing human e-mail counseling, computer-automated tailored counseling, and no counseling in an Internet weight loss program. Arch Intern Med.

[60] Thomas JG, Raynor HA, Bond DS, Luke AK, Cardoso CC, Foster GD, Wing RR (2017). Weight loss in Weight Watchers Online with and without an activity tracking device compared to control: A randomized trial. Obesity (Silver Spring).

[61] Thomas JG, Goldstein CM, Bond DS, Hadley W, Tuerk PW (2020). Web-based virtual reality to enhance behavioural skills training and weight loss in a commercial online weight management programme: The Experience Success randomized trial. Obes Sci Pract.

[62] Turner-McGrievy G, Tate D (2011). Tweets, Apps, and Pods: Results of the 6-month Mobile Pounds Off Digitally (Mobile POD) randomized weight-loss intervention among adults. J Med Internet Res.

[63] Turner-McGrievy GM, Wilcox S, Boutté A, Hutto BE, Singletary C, Muth ER, Hoover AW (2017). The dietary intervention to enhance tracking with mobile devices (DIET Mobile) study: a 6-month randomized weight loss trial. Obesity (Silver Spring).

[64] West DS, Harvey JR, Krukowski RA, Prewitt TE, Priest J, Ashikaga T (2016). Do individual, online motivational interviewing chat sessions enhance weight loss in a group-based, online weight control program?. Obesity (Silver Spring).

[65] West DS, Stansbury M, Krukowski RA, Harvey J (2019). Enhancing group-based internet obesity treatment: A pilot RCT comparing video and text-based chat. Obes Sci Pract.

[66] Womble LG, Wadden TA, McGuckin BG, Sargent SL, Rothman RA, Krauthamer-Ewing ES (2004). A randomized controlled trial of a commercial internet weight loss program. Obes Res.

[67] Collins CE, Morgan PJ, Hutchesson MJ, Callister R (2013). Efficacy of standard versus enhanced features in a Web-based commercial weight-loss program for obese adults, part 2: randomized controlled trial. J Med Internet Res.

[68] Collins CE, Morgan PJ, Hutchesson MJ, Oldmeadow C, Barker D, Callister R (2017). Efficacy of web-based weight loss maintenance programs: a randomized controlled trial comparing standard features versus the addition of enhanced personalized feedback over 12 months. Behav Sci (Basel).

[69] Duncan M, Fenton S, Brown W, Collins C, Glozier N, Kolt G, Holliday E, Morgan P, Murawski B, Plotnikoff R, Rayward A, Stamatakis E, Vandelanotte C, Burrows T (2020). Efficacy of a multi-component m-health weight-loss intervention in overweight and obese adults: a randomised controlled trial. Int J Environ Res Public Health.

[70] Hutchesson MJ, Callister R, Morgan PJ, Pranata I, Clarke ED, Skinner G, Ashton LM, Whatnall MC, Jones M, Oldmeadow C, Collins CE (2018). A targeted and tailored eHealth weight loss program for young women: the be positive be health randomized controlled trial. Healthcare (Basel).

[71] Jane M, Hagger M, Foster J, Ho S, Kane R, Pal S (2017). Effects of a weight management program delivered by social media on weight and metabolic syndrome risk factors in overweight and obese adults: A randomised controlled trial. PLoS One.

[72] Jane M, Foster J, Hagger M, Ho S, Kane R, Pal S (2018). Psychological effects of belonging to a Facebook weight management group in overweight and obese adults: Results of a randomised controlled trial. Health Soc Care Community.

[73] Morgan PJ, Lubans DR, Collins CE, Warren JM, Callister R (2009). The SHED-IT randomized controlled trial: evaluation of an Internet-based weight-loss program for men. Obesity (Silver Spring).

[74] Morgan PJ, Lubans DR, Collins CE, Warren JM, Callister R (2011). 12-month outcomes and process evaluation of the SHED-IT RCT: an internet-based weight loss program targeting men. Obesity (Silver Spring).

[75] Morgan PJ, Callister R, Collins CE, Plotnikoff RC, Young MD, Berry N, McElduff P, Burrows T, Aguiar E, Saunders KL (2013). The SHED-IT community trial: a randomized controlled trial of internet- and paper-based weight loss programs tailored for overweight and obese men. Ann Behav Med.

[76] Blomfield RL, Collins CE, Hutchesson MJ, Young MD, Jensen ME, Callister R, Morgan PJ (2014). Impact of self-help weight loss resources with or without online support on the dietary intake of overweight and obese men: the SHED-IT randomised controlled trial. Obes Res Clin Pract.

[77] Young MD, Callister R, Collins CE, Plotnikoff RC, Aguiar EJ, Morgan PJ (2017). Efficacy of a gender-tailored intervention to prevent weight regain in men over 3 years: A weight loss maintenance RCT. Obesity (Silver Spring).

[78] Carter MC, Burley VJ, Nykjaer C, Cade JE (2013). Adherence to a smartphone application for weight loss compared to website and paper diary: pilot randomized controlled trial. J Med Internet Res.

[79] Dombrowski SU, McDonald M, van der Pol M, Grindle M, Avenell A, Carroll P, Calveley E, Elders A, Glennie N, Gray CM, Harris FM, Hapca A, Jones C, Kee F, McKinley MC, Skinner R, Tod M, Hoddinott P (2020). Game of Stones: feasibility randomised controlled trial of how to engage men with obesity in text message and incentive interventions for weight loss. BMJ Open.

[80] Dombrowski SU, McDonald M, van der Pol M, Grindle M, Avenell A, Carroll P, Calveley E, Elders A, Glennie N, Gray CM, Harris FM, Hapca A, Jones C, Kee F, McKinley MC, Skinner R, Tod M, Hoddinott P (2020). Text messaging and financial incentives to encourage weight loss in men with obesity: the Game of Stones feasibility RCT. Public Health Res.

[81] Little P, Stuart B, Hobbs FR, Kelly J, Smith ER, Bradbury KJ, Hughes S, Smith PWF, Moore MV, Lean MEJ, Margetts BM, Byrne CD, Griffin S, Davoudianfar M, Hooper J, Yao G, Zhu S, Raftery J, Yardley L (2016). An internet-based intervention with brief nurse support to manage obesity in primary care (POWeR+): a pragmatic, parallel-group, randomised controlled trial. The Lancet Diabetes & Endocrinology.

[82] Little P, Stuart B, Hobbs FR, Kelly J, Smith ER, Bradbury KJ, Hughes S, Smith PW, Moore MV, Lean ME, Margetts BM, Byrne CD, Griffin S, Davoudianfar M, Hooper J, Yao G, Zhu S, Raftery J, Yardley L (2017). Randomised controlled trial and economic analysis of an internet-based weight management programme: POWeR+ (Positive Online Weight Reduction). Health Technol Assess.

[83] McConnon A, Kirk SF, Cockroft JE, Harvey EL, Greenwood DC, Thomas JD, Ransley JK, Bojke L (2007). The Internet for weight control in an obese sample: results of a randomised controlled trial. BMC Health Serv Res.

[84] Mueller J, Richards R, Jones RA, Whittle F, Woolston J, Stubbings M, Sharp SJ, Griffin SJ, Bostock J, Hughes CA, Hill AJ, Ahern AL (2022). Supporting weight management during COVID-19: a randomized controlled trial of a web-based, ACT-based, guided self-help intervention. Obes Facts.

[85] Mueller J, Richards R, Jones RA, Whittle F, Woolston J, Stubbings M, Sharp SJ, Griffin SJ, Bostock J, Hughes CA, Hill AJ, Boothby CE, Ahern AL (2023). Supporting Weight Management during COVID-19 (SWiM-C): twelve-month follow-up of a randomised controlled trial of a web-based, ACT-based, guided self-help intervention. Int J Obes (Lond).

[86] Simpson SA, Matthews L, Pugmire J, McConnachie A, McIntosh E, Coulman E, Hughes K, Kelson M, Morgan-Trimmer S, Murphy S, Utkina-Macaskill O, Moore L (2020). An app-, web- and social support-based weight loss intervention for adults with obesity: the HelpMeDoIt! feasibility RCT. Public Health Res.

[87] Simpson SA, Matthews L, Pugmire J, McConnachie A, McIntosh E, Coulman E, Hughes K, Kelson M, Morgan-Trimmer S, Murphy S, Utkina-Macaskill O, Moore LAR (2020). An app-, web- and social support-based weight loss intervention for adults with obesity: the 'HelpMeDoIt!' feasibility randomised controlled trial. Pilot Feasibility Stud.

[88] Sniehotta FF, Evans EH, Sainsbury K, Adamson A, Batterham A, Becker F, Brown H, Dombrowski SU, Jackson D, Howell D, Ladha K, McColl E, Olivier P, Rothman AJ, Steel A, Vale L, Vieira R, White M, Wright P, Araújo-Soares V (2019). Behavioural intervention for weight loss maintenance versus standard weight advice in adults with obesity: A randomised controlled trial in the UK (NULevel Trial). PLoS Med.

[89] Gemesi K, Winkler S, Schmidt-Tesch S, Schederecker F, Hauner H, Holzapfel C (2024). Efficacy of an app-based multimodal lifestyle intervention on body weight in persons with obesity: results from a randomized controlled trial. Int J Obes (Lond).

[90] Kempf K, Röhling M, Martin S, Schneider M (2019). Telemedical coaching for weight loss in overweight employees: a three-armed randomised controlled trial. BMJ Open.

[91] Kempf K, Röhling M, Stichert M, Fischer G, Boschem E, Könner J, Martin S (2018). Telemedical coaching improves long-term weight loss in overweight persons: a randomized controlled trial. Int J Telemed Appl.

[92] Kohl J, Brame J, Centner C, Wurst R, Fuchs R, Sehlbrede M, Tinsel I, Maiwald P, Fichtner UA, Armbruster C, Farin-Glattacker E, Gollhofer A, König D (2023). Effects of a web-based lifestyle intervention on weight loss and cardiometabolic risk factors in adults with overweight and obesity: randomized controlled clinical trial. J Med Internet Res.

[93] Roth L, Ordnung M, Forkmann K, Mehl N, Horstmann A (2023). A randomized-controlled trial to evaluate the app-based multimodal weight loss program zanadio for patients with obesity. Obesity (Silver Spring).

[94] Haapala I, Barengo NC, Biggs S, Surakka L, Manninen P (2009). Weight loss by mobile phone: a 1-year effectiveness study. Public Health Nutr.

[95] Markkanen JO, Oikarinen N, Savolainen MJ, Merikallio H, Nyman V, Salminen V, Virkkula T, Karppinen P, Oinas-Kukkonen H, Hukkanen J (2024). Mobile health behaviour change support system as independent treatment tool for obesity: a randomized controlled trial. Int J Obes (Lond).

[96] Teeriniemi A, Salonurmi T, Jokelainen T, Vähänikkilä H, Alahäivälä T, Karppinen P, Enwald H, Huotari M, Laitinen J, Oinas-Kukkonen H, Savolainen MJ (2018). A randomized clinical trial of the effectiveness of a Web-based health behaviour change support system and group lifestyle counselling on body weight loss in overweight and obese subjects: 2-year outcomes. J Intern Med.

[97] van Genugten L, van Empelen P, Boon B, Borsboom G, Visscher T, Oenema A (2012). Results from an online computer-tailored weight management intervention for overweight adults: randomized controlled trial. J Med Internet Res.

[98] van Wier MF, Ariëns GAM, Dekkers JC, Hendriksen IJ, Smid T, van Mechelen W (2009). Phone and e-mail counselling are effective for weight management in an overweight working population: a randomized controlled trial. BMC Public Health.

[99] van Wier MF, Dekkers J, Hendriksen I, Heymans M, Ariëns GAM, Pronk N, Smid T, van Mechelen W (2011). Effectiveness of phone and e-mail lifestyle counseling for long term weight control among overweight employees. J Occup Environ Med.

[100] van Wier MF, Dekkers JC, Bosmans JE, Heymans MW, Hendriksen IJ, Pronk NP, van Mechelen W, van Tulder MW (2012). Economic evaluation of a weight control program with e-mail and telephone counseling among overweight employees: a randomized controlled trial. Int J Behav Nutr Phys Act.

[101] Hesseldal L, Christensen JR, Olesen TB, Olsen MH, Jakobsen PR, Laursen DH, Lauridsen JT, Nielsen JB, Søndergaard J, Brandt CJ (2022). Long-term weight loss in a primary care-anchored eHealth lifestyle coaching program: randomized controlled trial. J Med Internet Res.

[102] Christensen JR, Hesseldal L, Olesen TB, Olsen MH, Jakobsen PR, Laursen DH, Lauridsen JT, Nielsen JB, Søndergaard J, Brandt CJ (2022). Long-term weight loss in a 24-month primary care-anchored telehealth lifestyle coaching program: Randomized controlled trial. J Telemed Telecare.

[103] Christensen JR, Laursen DH, Lauridsen JT, Hesseldal L, Jakobsen PR, Nielsen JB, Søndergaard J, Brandt CJ (2022). Reversing type 2 diabetes in a primary care-anchored eHealth lifestyle coaching programme in Denmark: a randomised controlled trial. Nutrients.

[104] Beleigoli A, Andrade AQ, Diniz MDF, Ribeiro AL (2020). Personalized web-based weight loss behavior change program with and without dietitian online coaching for adults with overweight and obesity: randomized controlled trial. J Med Internet Res.

[105] Berli C, Scholz U (2021). Long-term and transfer effects of an action control intervention in overweight couples: a randomized controlled trial using text messages. Front Psychol.

[106] Apiñaniz A, Cobos-Campos R, Sáez de Lafuente-Moríñigo A, Parraza N, Aizpuru F, Pérez I, Goicoechea E, Trápaga N, García L (2019). Effectiveness of randomized controlled trial of a mobile app to promote healthy lifestyle in obese and overweight patients. Fam Pract.

[107] Keshavarz M, Sénéchal M, Bouchard D (2023). Online circuit training increases adherence to physical activity: a randomized controlled trial of men with obesity. Med Sci Sports Exerc.

[108] Kim M, Kim Y, Go Y, Lee S, Na M, Lee Y, Choi S, Choi HJ (2020). Multidimensional cognitive behavioral therapy for obesity applied by psychologists using a digital platform: open-label randomized controlled trial. JMIR Mhealth Uhealth.

[109] Silina V, Tessma MK, Senkane S, Krievina G, Bahs G (2017). Text messaging (SMS) as a tool to facilitate weight loss and prevent metabolic deterioration in clinically healthy overweight and obese subjects: a randomised controlled trial. Scand J Prim Health Care.

[110] Burke L, Lee AH, Pasalich M, Jancey J, Kerr D, Howat P (2012). Effects of a physical activity and nutrition program for seniors on body mass index and waist-to-hip ratio: a randomised controlled trial. Prev Med.

[111] Wyke S, Hunt K, Gray C, Fenwick E, Bunn C, Donnan P (2015). Football Fans in Training (FFIT): a randomised controlled trial of a gender-sensitised weight loss and healthy living programme for men - end of study report. Public Health Research.

[112] Morgan PJ, Collins CE, Plotnikoff RC, Cook AT, Berthon B, Mitchell S, Callister R (2011). Efficacy of a workplace-based weight loss program for overweight male shift workers: the Workplace POWER (Preventing Obesity Without Eating like a Rabbit) randomized controlled trial. Prev Med.

[113] Melendez-Torres GJ, Sutcliffe K, Burchett HED, Rees R, Richardson M, Thomas J (2018). Weight management programmes: Re-analysis of a systematic review to identify pathways to effectiveness. Health Expect.

[114] Lally P, van Jaarsveld C, Potts H, Wardle J (2009). How are habits formed: Modelling habit formation in the real world. Euro J Social Psych.

[115] Cleo G, Isenring E, Thomas R, Glasziou P (2017). Could habits hold the key to weight loss maintenance? A narrative review. J Hum Nutr Diet.

[116] Schippers M, Adam PCG, Smolenski DJ, Wong HTH, de Wit JBF (2017). A meta-analysis of overall effects of weight loss interventions delivered via mobile phones and effect size differences according to delivery mode, personal contact, and intervention intensity and duration. Obes Rev.

[117] Patel ML, Wakayama LN, Bennett GG (2021). Self-monitoring via digital health in weight loss interventions: a systematic review among adults with overweight or obesity. Obesity (Silver Spring).

[118] Jo A, Coronel BD, Coakes CE, Mainous AG (2019). Is there a benefit to patients using wearable devices such as Fitbit or health apps on mobiles? a systematic review. Am J Med.

[119] Busetto L, Bettini S, Makaronidis J, Roberts CA, Halford JC, Batterham RL (2021). Mechanisms of weight regain. Eur J Intern Med.

[120] Wing RR, Hill JO (2001). Successful weight loss maintenance. Annu Rev Nutr.

[121] Wing RR, Phelan S (2005). Long-term weight loss maintenance. Am J Clin Nutr.

