# Supplementary material for: Evaluation of the Aspects of Digital Interventions That Successfully Support Weight Loss: Systematic Review With Component Network Meta-Analysis
Source: J Med Internet Res. 2025 May 22;27:e65443. doi: 10.2196/65443 (PMC12141966; doi:10.2196/65443)
Supplement: Multimedia Appendix 1 [file jmir_v27i1e65443_app1.docx]

### **Multimedia Appendix 1.** Search strategies for all databases.

**MEDLINE(R) Host: Ovid**

Ovid MEDLINE(R) ALL 1946 to November 19, 2023

Date searched November 20, 2023

1 exp Digital Technology/

2 Mobile Applications/

3 Wearable Electronic Devices/

4 exp Cell Phone/

5 (web or mobile or online or app or apps or internet or browser or virtual*).ti.

6 (smartphone* or (smart adj phone*)).tw.

7 ((web* or online or internet or virtual*) adj2 support*).ab.

8 (mobile adj (device* or health or phone*)).tw.

9 text messag*.tw.

10 (e-health or ehealth or m-health or mhealth).tw.

11 (SMS or MMS).tw.

12 wearable*.tw.

13 (facebook or twitter or snapchat or facetime or Tiktok or youtube or whatsapp or instagram).tw.

14 fitbit.tw.

15 web-based.tw.

16 (smart adj (device* or tech*)).tw.

17 (social adj (media or network*)).tw.

18 ((mobile or online or tablet or computer or phone) adj2 (app or apps)).ab.

19 (activity adj2 (tracker* or monitor*)).tw.

20 (Accelerometer* or Pedometer*).tw.

21 (Zoom or skype or "video conferenc*").tw.

22 (support adj2 email*).ab.

23 cell phone*.tw.

24 (electronic adj2 device*).tw.

25 (reddit or blog or blogs or blogging or webinar* or podcast*).tw.

26 virtual reality/

27 (virtual adj2 (realit* or world* or environment*)).tw.

28 3d vision.tw.

29 1 or 2 or 3 or 4 or 5 or 6 or 7 or 8 or 9 or 10 or 11 or 12 or 13 or 14 or 15 or 16 or 17 or 18 or 19 or 20 or 21 or 22 or 23 or 24 or 25 or 26 or 27 or 28

30 exp Overweight/

31 weight gain/ or weight loss/

32 obes*.tw.

33 (Weight adj2 (over or loss* or gain* or increas* or decreas* or management or control* or reduc* or change*)).tw.

34 ((body mass index or bmi) adj2 (loss* or gain* or increas* or decreas* or control* or reduc* or change*)).tw.

35 30 or 31 or 32 or 33 or 34

36 randomized controlled trial.pt.

37 controlled clinical trial.pt.

38 randomi?ed.ab.

39 placebo.ab.

40 drug therapy.fs.

41 randomly.ab.

42 trial.ab.

43 groups.ab.

44 36 or 37 or 38 or 39 or 40 or 41 or 42 or 43

45 exp animals/ not humans.sh.

46 44 not 45

47 35 and 46

48 29 and 47

**Embase search history**

**Embase <1974 to 2023 November 19>**

1 exp Digital Technology/

2 Mobile Application/

3 Wearable Electronic Devices/

4 exp Cell Phone/

5 (web or mobile or online or app or apps or internet or browser or virtual*).ti.

6 (smartphone* or (smart adj phone*)).tw.

7 ((web* or online or internet or virtual*) adj2 support*).ab.

8 (mobile adj (device* or health or phone*)).tw.

9 text messag*.tw.

10 (e-health or ehealth or m-health or mhealth).tw.

11 (SMS or MMS).tw.

12 wearable*.tw.

13 (facebook or twitter or snapchat or facetime or Tiktok or youtube or whatsapp or instagram).tw.

14 fitbit.tw.

15 web-based.tw.

16 (smart adj (device* or tech*)).tw.

17 (social adj (media or network*)).tw.

18 ((mobile or online or tablet or computer or phone) adj2 (app or apps)).ab.

19 (activity adj2 (tracker* or monitor*)).tw.

20 (Accelerometer* or Pedometer*).tw.

21 (Zoom or skype or "video conferenc*").tw.

22 (support adj2 email*).ab.

23 cell phone*.tw.

24 (electronic adj2 device*).tw.

25 (reddit or blog or blogs or blogging or webinar* or podcast*).tw.

26 virtual reality/

27 (virtual adj2 (realit* or world* or environment*)).tw.

28 3d vision.tw.

29 1 or 2 or 3 or 4 or 5 or 6 or 7 or 8 or 9 or 10 or 11 or 12 or 13 or 14 or 15 or 16 or 17 or 18 or 19 or 20 or 21 or 22 or 23 or 24 or 25 or 26 or 27 or 28

30 exp Obesity/

31 weight gain/ or weight loss/

32 obes*.tw.

33 (Weight adj2 (over or loss* or gain* or increas* or decreas* or management or control* or reduc* or change*)).tw.

34 ((body mass index or bmi) adj2 (loss* or gain* or increas* or decreas* or control* or reduc* or change*)).tw.

35 30 or 31 or 32 or 33 or 34

36 exp randomized controlled trial/

37 Controlled clinical trial/

38 random$.ti,ab.

39 randomization/

40 intermethod comparison/

41 placebo.ti,ab.

42 (compare or compared or comparison).ti.

43 ((evaluated or evaluate or evaluating or assessed or assess) and (compare or compared or comparing or comparison)).ab.

44 (open adj label).ti,ab.

45 ((double or single or doubly or singly) adj (blind or blinded or blindly)).ti,ab.

46 double blind procedure/

47 parallel group$1.ti,ab.

48 (crossover or cross over).ti,ab.

49 ((assign$ or match or matched or allocation) adj5 (alternate or group$1 or intervention$1 or patient$1 or subject$1 or participant$1)).ti,ab.

50 (assigned or allocated).ti,ab.

51 (controlled adj7 (study or design or trial)).ti,ab.

52 (volunteer or volunteers).ti,ab.

53 human experiment/

54 trial.ti.

55 or/36-54

56 (random$ adj sampl$ adj7 ("cross section$" or questionnaire$1 or survey$ or database$1)).ti,ab. not (comparative study/ or controlled study/ or randomi?ed controlled.ti,ab. or randomly assigned.ti,ab.)

57 Cross-sectional study/ not (exp randomized controlled trial/ or controlled clinical study/ or controlled study/ or randomi?ed controlled.ti,ab. or control group$1.ti,ab.)

58 (((case adj control$) and random$) not randomi?ed controlled).ti,ab.

59 Systematic review.ti,ab. not (trial or study).ti.

60 (nonrandom$ not random$).ti,ab.

61 "random field$".ti,ab.

62 (random cluster adj3 sampl$).ti,ab.

63 (review.ab. and review.pt.) not trial.ti.

64 "we searched".ab. and (review.ti. or review.pt.)

65 "update review".ab.

66 (databases adj4 searched).ab.

67 (rat or rats or mouse or mice or swine or porcine or murine or sheep or lambs or pigs or piglets or rabbit or rabbits or cat or cats or dog or dogs or cattle or bovine or monkey or monkeys or trout or marmoset$1).ti. and animal experiment/

68 Animal experiment/ not (human experiment/ or human/)

69 or/56-68

70 55 not 69

71 29 and 35 and 70

**APA Psycinfo search strategy**

APA PsycInfo <1806 to November Week 4 2023>

1 exp Digital Technology/

2 Mobile Applications/

3 exp wearable devices/

4 exp Cell Phone/

5 (web or mobile or online or app or apps or internet or browser or virtual*).ti.

6 (smartphone* or (smart adj phone*)).tw.

7 ((web* or online or internet or virtual*) adj2 support*).ab.

8 (mobile adj (device* or health or phone*)).tw.

9 text messag*.tw.

10 (e-health or ehealth or m-health or mhealth).tw.

11 (SMS or MMS).tw.

12 wearable*.tw.

13 (facebook or twitter or snapchat or facetime or Tiktok or youtube or whatsapp or instagram).tw.

14 fitbit.tw.

15 web-based.tw.

16 (smart adj (device* or tech*)).tw.

17 (social adj (media or network*)).tw.

18 ((mobile or online or tablet or computer or phone) adj2 (app or apps)).ab.

19 (activity adj2 (tracker* or monitor*)).tw.

20 (Accelerometer* or Pedometer*).tw.

21 (Zoom or skype or "video conferenc*").tw.

22 (support adj2 email*).ab.

23 cell phone*.tw.

24 (electronic adj2 device*).tw.

25 (reddit or blog or blogs or blogging or webinar* or podcast*).tw.

26 virtual reality/

27 (virtual adj2 (realit* or world* or environment*)).tw.

28 3d vision.tw.

29 1 or 2 or 3 or 4 or 5 or 6 or 7 or 8 or 9 or 10 or 11 or 12 or 13 or 14 or 15 or 16 or 17 or 18 or 19 or 20 or 21 or 22 or 23 or 24 or 25 or 26 or 27 or 28

30 exp Overweight/

31 weight gain/ or weight loss/

32 obes*.tw.

33 (Weight adj2 (over or loss* or gain* or increas* or decreas* or management or control* or reduc* or change*)).tw.

34 ((body mass index or bmi) adj2 (loss* or gain* or increas* or decreas* or control* or reduc* or change*)).tw.

35 30 or 31 or 32 or 33 or 34

36 exp Randomized Controlled Trials/ or exp Treatment Effectiveness Evaluation/

37 exp Placebo/

38 randomi?ed.ab.

39 placebo.ab.

40 exp Treatment Outcomes/

41 randomly.ab.

42 trial.ab.

43 groups.ab.

44 36 or 37 or 38 or 39 or 40 or 41 or 42 or 43

45 exp animals/ not humans.sh.

46 44 not 45

47 35 and 46

48 29 and 47

**Cochrane Central search history**

Search Name: WS2 CENTRAL 20 Nov 2023 5699 hits

ID Search Hits

#1 MeSH descriptor: [Overweight] explode all trees

#2 MeSH descriptor: [Weight Loss] explode all trees

#3 overweight:ti,ab

#4 obes*:ti,ab

#5 (weight:ti,ab NEAR/2 (over:ti,ab OR loss*:ti,ab OR control*:ti,ab OR reduc*:ti,ab OR change*:ti,ab OR increas*:ti,ab OR decrease*:ti,ab OR management:ti,ab))

#6 (("body mass index":ti,ab OR bmi:ti,ab) NEAR/2 (over:ti,ab OR loss*:ti,ab OR control*:ti,ab OR reduc*:ti,ab OR change*:ti,ab OR increas*:ti,ab OR decrease*:ti,ab OR management:ti,ab))

#7 #1 or #2 or #3 or #4 or #5 or #6

#8 (digital OR digitally):ti,ab

#9 MeSH descriptor: [Digital Technology] explode all trees

#10 (web OR mobile OR online OR app OR apps OR internet OR browser OR virtual):ti

#11 MeSH descriptor: [Mobile Applications] explode all trees

#12 ((mobile OR online OR tablet OR computer OR phone) NEAR/2 (app OR apps)):ab

#13 ((mobile OR online OR tablet OR computer OR phone) NEAR/2 (application)):ti,ab

#14 ((smartphone*) OR ("smart" NEXT phone*)):ti,ab

#15 ((web OR online OR internet OR virtual*) NEAR/2 (support*)):ab

#16 ((mobile) NEXT (device* OR health OR phone*)):ab

#17 MeSH descriptor: [Text Messaging] explode all trees

#18 ("text" NEXT message*):ti,ab

#19 (ehealth OR e-health OR mhealth OR m-health):ti,ab

#20 (sms OR mms):ti,ab

#21 wearable*:ti,ab

#22 (facebook OR twitter OR snapchat OR facetime OR youtube OR "whats app" OR instagram OR tiktok):ti,ab

#23 fitbit:ti,ab

#24 web-based:ti,ab

#25 MeSH descriptor: [Wearable Electronic Devices] explode all trees

#26 ((smart) NEXT (device* OR tech*)):ti,ab

#27 ((social) NEXT (media* OR network*)):ti,ab

#28 #8 or #9 or #10 or #11 or #12 or #13 or #14 or #15 or #16 or #17 or #18 or #19 or #20 or #21 or #22 or #23 or #24 or #25 or #26 or #27

#29 #7 AND #28

#30 (activity NEAR/2 (tracker* or monitor*)):ti,ab

#31 (Accelerometer* or Pedometer*):ab

#32 (zoom or skype):ti,ab

#33 (video conferenc*):ti,ab

#34 (support NEAR/2 email):ab

#35 MeSH descriptor: [Cell Phone] explode all trees

#36 MeSH descriptor: [Smartphone] explode all trees

#37 cell phone*:ab

#38 (reddit or blog or blogs or blogging or webinar* or podcast*):ti,ab

#39 #30 or #31 or #32 or #33 or #34 or #35 or #36 or #37 or #38

#40 #7 and #39

#41 #40 not #29

#42 ((web or online or internet or virtual*) NEAR/2 (support*)):ab

#43 ((mobile or online or tablet or computer or phone) NEAR/2 (app or apps)):ab

#44 #42 or #43

#45 #28 OR #39 OR #44

#46 #7 AND #45

We selected Trials with source Embase, Pubmed or Cinahl
