# Supplementary material for: Evaluation of the Aspects of Digital Interventions That Successfully Support Weight Loss: Systematic Review With Component Network Meta-Analysis
Source: J Med Internet Res. 2025 May 22;27:e65443. doi: 10.2196/65443 (PMC12141966; doi:10.2196/65443)
Supplement: Multimedia Appendix 3 [file jmir_v27i1e65443_app3.docx]

**Multimedia Appendix 3.** Sample characteristics.

| **First author, date** | **Country** | **Stated aim** | **Funding** | **Conflict of interest** | **N** | **Baseline characteristics** | | | | | | **Outcomes** |
| --- | --- | --- | --- | --- | --- | --- | --- | --- | --- | --- | --- | --- |
|  |  |  |  |  |  | **BMI (kgm^2^) (mean/ median, SD, range)** | **Age (yrs) (mean, SD)** | **% female** | **Ethnicity/ race (%)** | **SES (%)** | **Co-morbidities** |  |
| Apiñaniz 2019[1] | Spain | WL | NA | None to disclose | 100 | 32.7 (4.9) | 38.5 (5.0) | 72.0% | NR | NR | Hypothyroidism 10.1%, Baseline glucose lowering treatment 0.1%, Baseline cholesterol lowering treatment 1.9%, Baseline hypertension treatment 5.5% | Anth, clin, other |
| Backman 2023[2] | USA | WL | California Department of Social Services and Forestry and Fire Protection | NR | 70 | 32.5 (4.2) | 34 (6.4) | 100.0% | Hispanic/ Latino ~45.0%,American Indian/Alaska Native ~ 6.0%, Asian ~10.0%, Black/African American ~ 37.0%, White ~30.0%, Pacific Islander~ 1.0%, Other ~ 29.0% | Some secondary school ~6%; secondary school graduate ~ 21%; some college or higher ~ 73% | Diabetes ~7.0% | Anth, diet, PA, WB, other |
| Beleigoli 2020[3] | Brazil | WL | National Institute of Science and Technology for Health Technology Assessment (CNPq) of Brazil | AQA owner of software company providing web platform | 1298 | 29.89 (29.66-30.13) | 33.6 (33.0-34.2) | 77.0% | NR | NR, | NR | Anth, diet, PA, other |
| Berli 2021[4] | Switzerland | WL, PA | Swiss National Science Foundation | None to disclose | 121 | 31.0 (5.5) | 46.1 (13.5) | 51.3% | NR | 65.3% employed | NR | Anth, PA |
| Bennett 2013,[5]  Lanpher 2016[6] | USA | WM | NIDDK | None to disclose | 185 | 30.2 (2.5) | 35.4 (5.5) | 100% | Black 100% | Education: < High school 10.4%, High school 24.2%, Vocational or trade school after high school 8.8%, Some college 36.3%, College or above 20.3%; Employment: Employed 71.4%; Income: <$10 000 20.8%, $10 000–19 999 28.4%, $20 000–29 999 25.1%, >$30 000 25.7%; Poverty: Yes 33.0%, Borderline 29.1%, No 37.0%** | Hypertension 36.4%, Diabetes 6.5%, Metabolic syndrome 30.8%, Depression 21.5% | Anth, clin |
| Braun 2022[7] | USA | WL | National Institute on Minority Health and Health Disparities; National Heart, Lung, and Blood Institute; National Center for Complementary and Integrative Health; NIDDK | None to disclose | 28 | 46.6 (3.7) | 48.8 (10.4) | 100.0% | NR | NR | NR | Anth, MR, WB, other |
| Burke 2022[8, 9] | USA | WL | NIH | None to disclose | 502 | 33.7 (4.0) | 45 (14.4) | 79.5% | White 84.3%, Black or African American 12.4%, American Indian 0.2%, Asian 3.1%, | Education (years): 16.4 (2.8) Employed: Full time/part time 82.1%, Unemployed 17.9%. Household income: < $60,000 28.5%, ≥ $60,000 62.4% | High BP 21.1%, High cholesterol 17.5%, High triglycerides 11.2% | Anth, clin |
| Burke 2011,[10]2012,[11] Turk2013[12]  Conroy 2011[13] | USA | WL | Weight Watchers International Inc | None to disclose | 210 | 34.1 (4.5) | 46.8 (9.0) | 84.8% | White 78.6% | Full time employment 82.9%, Education (years) 15.7 (3.0) | NR | Anth, diet |
| Carter 2013[14] | UK | WL | National Prevention Research Initiative grant | Authors developed the My Meal Mate (MMM) app for this trial with a software company (Blueberry Consultants) but University of Leeds owns full intellectual property and the researchers have no commercial intent with the app | 128 | 34.2 (5.2) | 41.9 (9.1) | 77.0% | White 91% | 58.0% managerial or professional occupation, 60.0% University degree | NR | Anth, other |
| Collins 2013[15] | Australia | WL | Australian Research Council Linkage Project grant with SP Health as the Industry Partner Organization | CEC has been a nutrition consultant to SP Health Co. MJH (nee Neve) and was funded by a Penn Health postdoctoral fellowship | 301 | 32.2 (3.9) | 41.9 (10.2) | 58.5% | NR | Highest level of education: High school 29.9%, Trade/ diploma 34.6%, University degree 2.6%, Higher university degree 12.6%; Weekly household income: <$700 8%, $700 to <$1000 5.6%, $1000 to <$1400 11.6%, ≥$1500 66.4% | NR | Anth, clin, MH, PA, WB, other |
| Collins 2017[16] | Australia | WM | Australian Research Council Linkage Project grant with SP Health as the Industry Partner Organization | None to disclose | 227 | 30.4 (4.1) | 42.3 (10.1) | 66.0% | NR | Highest level of education: School 29.0%, Trade/ diploma 35.0%, University degree 23.0%, Higher university degree 13%; Weekly household income: <$700 8.8%, $700 to <$1000 5.1%, $1000 to <$1400 11.0%, $1500 or more 72.0% | NR | Anth, clinical |
| Conroy 2019[17] | USA | WM | Agency for Healthcare Research and Quality and National Institutes of Health | KMM reports a patent with royalties paid. | 194 | 30.4 (5.9) | 53.4 (12.2) | 74.0% | White 88%, Hispanic/ Latino 1.5% | 95.3% post-secondary education | High cholesterol 42.8%, Hypertension 49.0 %,Knee and/or hip arthritis 30.0%, Diabetes/ prediabetes 21.6%, Anxiety/depression 27.3% | Anth, clin, diet, MH, PA, WB |
| Dombrowski 2020[18] | UK (Scotland) | WL | NIHR | MG is director of Eos Digital Health Ltd, which holds all rights to DNA, The Digital Narrative Approach, deployed to design and write the narrative text intervention. | 105 | 35.7 (5.9) | 52.2 (13.1) | 0.0% | White 91.4%, Asian 3.8%, Black 3.8%, Prefer not to say 1% | SIMD deprivation category 1(most disadvantaged): 36.5%, category 2: 23.1%, category 3: 11.5%, category 4: 13.5%, category 5: 15.4%; Bachelor degree 23.3%, HNC/HND 11.6%, Higher grade/ advanced higher/A-level or equivalent 8.7%, Masters/PhD or equivalent 4.8%, No formal qualifications 19.4%, Standard grade/GCSE/ intermediate 1 or 2 16.5%, still studying 5.8%, Vocational qualifications 4.8%, Other 1%, Prefer not to say 3.9%; Full time student 6.7%, Employed full-time 48.1%, Employed part-time 5.8%, Self-employed 6.7%, Not in paid work 15.4%, Retired 17.3% | Arthritis 21.0%, Cancer 1.0%, Diabetes 14.3%, Myocardial infarction 2.9%, Hypertension 21.0%, Stroke (inc TIA) 1.9% | Anth, other |
| Duncan 2020[19] | Australia | WL | Fellowship support funding from the National Health and Medical Research Council and University of Newcastle, Faculty of Health, and Medicine. Project partly supported by Diabetes Australia General Grant | None to disclose | 116 | 31.7 (3.9) | 44.5 (10.4) | 70.7% | NR | Education (mean, y) 16.4 (3.0), Employed: Full-time 53.4%, Part-time/casual 33.6%, Retired 5.2%, Other 7.8%. | NR | Anth, clin, diet, MH, PA, WB, other |
| Dunn 2019[20] | USA | WL | Academy of Nutrition and Dietetics Foundation Amy Joye Memorial Research Award | None to disclose | 43 | 34.5 (5.7) | 42.4 (12.4) | 90.7% | White 81.4%, Black 16.3%, Other 2.3% | Education: High school 2.3%, Some college 18.6%, College graduate 30.3%, Advanced degree 48.8%; Occupation: No current employment 4.7%, Service occupation 2.3%, Technical, sales, administrative 11.6%, Executive, managerial 11.6%, Professional speciality 39.5%, Retired 2.3%, Other 30.0% | NR | Anth, other |
| Eisenhauer 2021[21] | USA | WL | National Institute of Nursing Research of the National Institute of Health | None to disclose | 80 | 35.6 (6.9) | 54.2 (8.6) | 0.0% | White 97.5%, Black or African American 1.3%, Asian 1.3% | Highest education: High school graduate 7.5%, College 16.3%, Associate degree 17.5%, Bachelor's degree 30%, Master's degree 22.5%, Doctoral degree 6.3%; Household income: <$20,000 1.3%, $20,000–$39,000 7.7%, $40,000–$59,000 9%, $60,000–$79,000 20.5%, $80,000–$99,000 24.4%, ≥$100,000 37.2%; employed 90% | NR | Anth, clin, diet |
| Falkenhain 2021[22] | USA | WL, RR | Canadian Institutes of Health Research, Michael Smith Foundation for Health Research | JPL is chief scientific officer for the Institute for Personalized Therapeutic Nutrition and holds shares in Metabolic Insights Inc. EJW is an equity holder at Keyto and Virta Health. DAL is employed as a consultant for Keyto | 155 | 33.5 (4.7) | 41.0 (11) | 71.0% | NR | NR | NR | Anth, clin |
| Gemesi 2024[23] | Germany | WL | Oviva AG, Potsdam, Germany | HH is a member of the scientific advisory board of Oviva AG. CH is a member of the scientific advisory board of the 4sigma GmbH. HH and CH received speaker´s honoraria from Novo Nordisk | 181 | 34.2 (2.8) | 46.9 (11.1) | 64.3% | NR | Education (yrs): 8/9 7.8%, 10 21.5%, 12/13 16.7%, University/ college 53.6%, Other 0.6%; Vocational Education: y 66.1%,n 32.7%, NA 1.2%; Working: Full time 62.5%, Part time 23.8%, Other 13.7% | Thyroid disease 17.3%, Hypertension 14.9%, | Anth, WB |
| Gold 2007[24] | USA | WL | U.S. Department of Agriculture Hatch Act Funds | SB was employee of eDiets.com for study duration | 124 | 32.4 (4.1) | 47.7 (10.3) | 82.0% | White 98% | Education: high school 5%, some college 26%, college degree 38%, graduate/professional degree 31.5% | NR | Anth, diet, PA |
| Haapala 2009[25] | Finland | WL | Partly funded by GeraCap Invia Ltd | I.H. received consultation fee from GeraCap Invia Ltd. and has served on the executive board of the Weight BalanceR programme (since 2008) | 125 | 30.5 (2.8) | 38.1 (4.7) | 78.0% | NR | Education: Vocational school 17.0%, College/ baccalaureate 60.5%, Graduate degree 15.5% | NR | Anth, diet |
| Hageman 2017[26] | USA | WL | National Institute of Nursing Research, National Institutes of Health Grant, Research Support Fund Grant from The Nebraska Medical Center and the University of Nebraska Medical Center for lipid and glucose testing and from The Hygenic Corporation Thera-Band Academy for equipment support | PAH and CHP declare a patent copyright on the “Banding Together for Strength” DVD used in intervention which participant baseline data analysed | 301 | Overweight (25-29.9) 10.7%, Obese I (30-34.9) 47.7%, Obese II (35-39.9) 28.3%, Obese III (≥40 to 45) 13.7% | 53.9 (6.9) | 100.0% | White 97.3%, Hispanic 1%, Other 1.3%, NR 0.3% | Education: High school or lower 15.7%, some college 43.7%, College grad or above 40.7%; Employment: Full-time 68.7%, Part-time 17.7%.House- hold income <$20,000 2.7%, $20,000 to $39,999 14.3%, $40,000 to $59,999 26%, $60,000 or higher 50.7%; Rural residency: large rural 62.7%, small rural 8.0%, isolated 28.7% | Diabetes 4.7%, Respiratory 6.0%, Arthritis 26.7%, Other muscular conditions 10.7%, Cancer 9%, Thyroid 23.3%, Cardiovascular 2.7%, General 35.0% | Anth, clin, diet, PA |
| Hesseldal 2022,[27]  Christensen 2022[28] | Denmark | WL | No external funding | DHL was financially supported by and is employed at LIVA Healthcare A/S. DHL primarily contributed information about the use of the LIVA app in clinical practice. CJB works as a consultant, owns stocks in and is an original cofounder of LIVA Healthcare A/S. A formal research agreement made between the University of Southern Denmark and LIVA Healthcare A/S to guarantee that LIVA Healthcare A/S cannot influence any results of study. | 340 | 35.3 (3.8) | 52.3 (11) | 64.0% | NR | Education: None 17.0%, Short 26.0%, Medium 46.0%, Long 11.0%, DK 1.0%; Employment: Employed 72.0%, Out of work (maternity, un-employment, or cash benefits) 8.0%, Retired 19.0%, Student 2.0% | Diabetes 49% | Anth, clin |
| Hutchesson 2018[29] | Australia | WL | University of Newcastle New Staff Grant | None to disclose | 57 | 29.4 (2.5) | 27.1 (4.7) | 100.0% | NR | Highest level of education: Higher university (Masters, PhD) 7.0%, University degree 25.1%, Certificate/ diploma 26.3%, Trade/apprenticeship 3.5%, currently studying for first degree 26.3%, None 1.8%; Individual income: Lower ($0–$299/week) 22.8%, Middle ($300–$999/week) 43.9%, Higher ($1000 or more/week 33.3% | NR | Anth, clin, diet, PA, other |
| Jane 2017,[30]2018[31] | Australis | WL, RR | No external funding | None to disclose | 137 | 32.9*(1.2) | 50.4 (2.3) | 85.1% | NR | NR | NR | Anth, clin, diet, MH, PA, WB |
| Joseph 2023[32] | USA | Improved PA, RR | The National Institutes of Health/National Heart, Lung, and Blood Institute | None to disclose | 60 | 40.6 (7.0) | 38.4 (6.9) | 100.0% | African American 100.0% | Education: Some high school 1.5%, High school diploma or GED 12.0%, Some college or technical school 40.0%, Bachelor’s degree 20%, Master’s degree 25.0%, Doctoral degree 1.5%; Annual income: <USD 25K 15.0%, USD 25,001–USD 50K 38.5%, USD 50,001–USD 75K 36.5%, USD 75,001–USD 100K 8.5%, >USD 100K 1.5%; Employment status: Employed, Part-time 17.0%, Employed, Full-time 73.0%, Currently unemployed 10.0% | NR | Anth, clin, PA, other |
| Kempf 2019[33] | Germany | WL | Boehringer Ingelheim International GmbH  and Gesellschaft von Freunden und Förderern der Heinrich-Heine-Universität  Düsseldorf | KK and SM received research support from Boehringer  Ingelheim International GmbH & Co. KG. MS is an employee and SM is a member of the advisory board. | 104 | 31.0 (5.0) | 50.1 (5.3) | 16.0% | NR | NR | NR | Anth, clin, other |
| Kempf 2018[34] | Germany | WL | NR | KK and SM received research support from HMM Holding AG | 180 | 33.2 (4.7) | 45.4 (10) | 55.0% | NR | NR | Overweight (BMI <30 kg/m2) 27.9%, Moderately obese (BMI 30-34,9 kg/m2) 39.4%, Severely obese (BMI 35-39,9 kg/m2) 23.4%, Very severely obese (BMI >40 kg/m2) 9.4% | Anth, clin |
| Keshavarz 2023[35] | Canada | Improved PA | Heart and Stroke Foundation of New Brunswick | None to disclose | 60 | 28.9 (3.4) | 41.7 (11.4) | 0.0% | NR | Annual household income (≤$100,000): 25.2%; Education (college or higher): 92.7%. | Overweight (25.0–29.9 kg·m2) 63.3%, Obesity level 1 (30.0–34.9 kg·m2) 20.0% | Anth, clin |
| Kharmats 2022[36] | USA | WL | Lerner Center for Public Health Promotion | AYK received doctoral training funding from the Johns Hopkins CLF Lerner Fellowship, and a postdoctoral training grant from the NIH. LH received funding from NIH and the New York Regional Center for Diabetes Translation Research | 155 | 35.5 (7.0) | 44.6 (13.4) | 87.0% | Race: Black/African American 53.5%, White/Caucasian 33.5%, Asian 6.5%, Other 5.8%, NR 0.6%; Ethnicity: Non-Hispanic 93.5%, Hispanic 3.9%, NR 2.6% | Education: < high school 5.8%, high school/GED 24.5%, Associates/trade/vocational 7.7%, bachelor’s degree 31.6%, master’s degree 22.6%, Professional or doctoral 7.1%, Not reported 0.6%; Employed: 78.7%; Benefits (receiving SNAP): 18.1% | Overweight (30> BMI ≥25) 22.6%, Class I obesity (35> BMI ≥30) 31.6%, Class II obesity (40> BMI ≥35) 22.6%, Class III obesity (BMI ≥40) 23.2% | Anth, diet, PA, other |
| Kim 2020[37] | Republic of Korea | WL | Funded by Noom. Study supported by a grant from National Research Foundation of Korea, funded by the Korean Government, Ministry of Science and ICT. | YK is an employee of Noom | 70 | 28.0 (3.2) | 21.8 (3.3) | 100.0% | NR | NR | NR | Anth, diet, MH, WB, other |
| Kohl 2023[38] | Germany | WL, RR | Techniker Krankenkasse (German Health Insurance Company) | The Department of Sport and Sport Science (JK, JB, RW, DK, and RF) and Section for Health Services Research and Rehabilitation Research (SEVERA) (MS, IT, EFG, CA, UAF, and PM) were commissioned by Techniker Krankenkasse for clinical trial design, implementation, and scientific evaluation of the online trial and web-based program | 153 | 30.7 (2.1) | 48.9 (11.2) | 71.2% | NR | NR | NR | Anth, diet, clin, PA |
| Kurtzman 2018[39] | USA | WL | University of Pennsylvania CTER and the McCabe Fund | MSP is principal at Catalyst Health. MSP supported by career development awards from Department of Veterans Affairs HSR&D and also received research funding from Deloitte, which is not related to work described in manuscript | 196 (98 teams) | 36.2 (5.3) | 41.4 (11.7) | 85.7% | White, non-Hispanic 61.3%, African American non-Hispanic 30.1%, Other non-Hispanic 5.6%, Hispanic 2.5% | Education: Less than college graduate 30.1%, College graduate 69.9%. Annual household income:<$50,000 20.9%, $50,000 to $100,000 32.6%, > $100,000 37.7%, Unknown 8.7% | NR | Anth, PA |
| Laing 2014[40] | USA | WL | Robert Wood Johnson Foundation Clinical Scholars Program | None to disclose | 212 | 33.3 (7.0) | 43.2 (14.5) | 73.0% | Hispanic 32.5%, White 47.5%, Black 19%, Asian 8.5%, Native American or Pacific Islander 0.75% | Education: High school or less 18.5%, Some college or college graduate 59.0%, < 4yr college 22.0%. Annual income: < $30 000 26.5%, $30 000–$49 000 17.5%, $50 000–$74 999 20.0%, ≥$75 000 37.0% | NR | Anth, clin, PA, WB, other |
| La Rose 2022[41] | USA | WL | National Institute of Diabetes and NIDDK and Virginia Commonwealth University Clinical and Translational Science Award | JGL and EPW received grant funding from, JGL and AL served as consultants and DFT served on the Scientific Advisory Board for WW International. All outside the submitted work. JLF received grants from Lifespan/The Miriam Hospital during the conduct of the study | 382 | 33.5 (4.9) | 21.9 (2.2) | 82.7% | Ethnicity: Hispanic/ Latino 12.8%, Non-Hispanic 87.2%; Race: American Indian or Alaska Native only 0.8%, Asian only 5.8%, Black only 33.8%, Multi-racial 7.9%, White only 47.1%, Another race not listed 4.5%, NR 0.3% | Education: High school (10-12yr) 9.2%, Vocational training 1.0%, Some college (<4 y) 48.2%, College degree 35.1%, Graduate or professional degree 6.5%; Work or school: Neither 2.1%, Work only 30.9%, School only 22.5%, School & work 44.5%; Income mean: $21 450 (15 631) | NR | Anth, clin |
| Leahey 2015[42] | USA | WL | NIDDK | TML reported received compensation for consulting services provided to Weight Watchers, Inc. RK is the CEO and is a board member of the Shape Up Rhode Island parent company, ShapeUp, Inc. BW is shareholder of ShapeUp, Inc | 268 | 33.6 (6.3) | 46.3 (10.5) | 82.5% | Non-Hispanic White 88.7%, Non-white 11.3% | Education: Vocational/ high school 7.9%, Some college 19.1%, College 42.7%, Post-graduate 30.3% | NR | Anth, other |
| Leahey 2016[43] | USA | WM | NIH | TML is currently Chief Scientist and a paid consultant at WayBetter, Inc.; however, the work presented herein is not at all related to her relationship with WayBetter | 75 | 31.5 (5.8) | 48.5 (10.7) | 85.3% | Race: White 88.0%, Non-white 12.0%; Ethnicity: Non-Hispanic/ Latino 97.3%, Hispanic /Latino 1.3%, NR 1.3% | Education: High school (10-12 y) 4.0%, Vocational 5.3%, Some college (<4 y) 17.3%, College/univ grad 38.7%, Grad/prof school 34.7%; Income: 0–$25,000 2.7%, $25,001–$50,000 13.3%, $50,001–$75,000 20.0%, $75,001–$100,000 18.7%, $100,001–$125,000 21.3%, $125,000+ 24.0% | NR | Anth |
| Little 2016,[44]2017[45] | UK (England) | WL | HTA programme of the NIHR | None to disclose | 826 | 36.7 (5.7) | 53.6 (13.1) | 63.7% | NR | Deprivation score (IMD 2010): 13.8 (10.3) | Comorbidity (not specified) 19.0% | Anth, other |
| Markkanen 2024[46] | Finland | WL | Business Finland, Novo Nordisk Foundation, Suorsa Healthcare Foundation and the Finnish Foundation for Cardiovascular Research | HOK, TV, MJS (founders) and VN are shareholders of Onnikka Health Oy that owns the intellectual property rights of the mHBCSS described in this paper | 200 | 34.3 (2.8) | 46.5 (9.5) | 89.0% | NR | Education: High school graduate 71.0%, College or university graduate 73.0% | Medications: Antihypertensives 34.0%, Diabetes 8.0%, Cholesterol 9.0% | Anth, clin |
| McConnon 2007[47] | UK (England) | WL, CE | The Health Foundation | None to disclose | 221 | Median (IQR) BMI 34.4 (31.9–38.7) | 45.8 (10.6) | 77.0% | White 95% | NR | NR | Anth, PA, other |
| Morgan 2009,[48] 2011[49] | Australia | WL | University of Newcastle Strategic Pilot Grant. | None to disclose | 65 | 30.6 (2.8) | 35.9 (11.1) | 0.0% | NR | SES status: 1–2 (lowest) 1.9%, 3-4 23.1%, 5-6 23.1%, 7-8 42.3%, 9-10 (highest) 5 9.6%; Occupation: Student 43.0%, Non-academic staff 41,5%, Academic staff 15.4% | BMI: Overweight 47.7%,Obese 52.3% | Anth, clin, diet, PA, other |
| Morgan 2013,[50] Blomfield 2014[51] | Australia | WL | National Heart Foundation Grant-in-Aid | None to disclose | 159 | 32.7 (3.5) | 47.5 (11.0) | 0.0% | NR | SES: 1–2 (lowest) 5.7%, 3-4 15.7%, 5-6 36.5%, 7-8 29.6%, 9-10 (highest) 12.6%.; Highest education: School 26.4%, Trade/diploma 49.1%, University 24.5%. Weekly household income <$1,000 12.7%, $1,000 to<$1,500 17.7%, $1,500 or more 66.5%, Unknown 3.2% | NR | Anth, clin, diet, PA, other |
| Mueller2022,[52] 2023[53] | UK | WL | Medical Research Council, NIHR, EASO | A.J.H. has consulted for Slimming World. C.A.H. reports payment or honoraria from Ethicon, Novo Nordisk, and International Medical Press for lectures, presentations, speakers’ bureaus, manuscript writing, or educational events. J.M. and R.R. are Trustees for the Association of the Study of Obesity (unpaid roles). A.L.A. and S.J.G. are the chief investigators on two publicly funded (MRC, NIHR) trials where the intervention is provided by WW (formerly Weight Watchers) at no cost outside the submitted work. | 388 | 34.8 (7.7) | 50.3 (13.8) (range 18-86) | 78.2% | White 93.8%, non-white 5.2%, none of these 0.3%, prefer not to say 0.8% | Education: below post-secondary (up to and including A-levels) 25.8%, post-secondary 72.7%, other 1.3%, prefer not to say 0.3% | BMI 25-<30 19.9%, 30-<40 50.4%, 40+ 19.9% | Anth, MH, PA, WB, other |
| Olson 2016,[54] Wipfli 2019[55] | USA | WL | National Heart, Lung, and Blood Institute | Oregon Health & Science University and WKA have a significant financial interest in Northwest Education Training and Assessment, a company that may have a commercial interest in the results of this research and technology. This was reviewed and managed by OHSU Conflict of Interest in Research Committee | 472 | 35.6 (0.6) | 47.8 (11.4) | 13.6% | Race: American Indian/ Alaskan Native 1.4%, Asian 0.2%, Native Hawaiian/ Pacific Islander 0.7%, Black/African American 7.4%, White 78.7%, >1 Race 6.1%, Other 5.7%; Ethnicity: Hispanic 10.1% | Education: High-school diploma or GED 51.9%, Vocational/ technical certificate 27.6%, Associate degree 11.8%, Bachelor’s degree 6.9%, Graduate degree 1.9% | Diabetes 12.5%, High blood pressure 34.2%, Obstructive sleep apnoea 18.1% | Anth, diet, other |
| Patel 2019[56] | USA | WL | Grant from American Psychological Association, the Duke Interdisciplinary Behavioral Research Center, and the Aleane Webb Dissertation Research Award provided by The Graduate School at Duke University | GGB serves on the scientific advisory board of Nutrisystem and Interactive Health; he holds equity in Coeus Health. | 100 | 31.9 (4.5) | 42.7 (11.7) | 84.0% | Non-hispanic white 67.0%, Non-hispanic black 22.0%, Hispanic (all races) 3.0%, Non-hispanic Other 8.0% | Education: <College graduate 17.0%, College graduate or above 83.0%; Employment status: Employed full-time 67.0%, Employed part-time 11.0%, Not employed 22.0%. Annual household income: $0-$49,999 26.0%, $50,000-$99,999 36.0%, $100,000 or greater 34.0%, Unknown/NR 4.0% | Overweight 25-29.9 kg/m2 40.0%, class 1 obesity, 30-34.9 kg/m2 38.0%, class 2 obesity, 35-39.9 kg/m2 17.0%, class 3 obesity, 40+ kg/m2 5.0% | Anth |
| Patrick 2011[57] | USA | WL | NIH/NCI | KP, KJC and JFS are co-owners of Santech, Inc. (San Diego, CA) which is developing products related to the research described in this paper. San Diego State University and the University of California, San Diego, have approved terms in accordance with their respective conflict of interest policies | 441 | 34.2 (4.1) | 43.9 (8.0) | 0.0%` | White non-hispanic 71.0%, Black non-hispanic 5.2%, Hispanic 18.1%, Asian/Pacific Islander 1.6%, Native American 0.5%, Multi-ethnic 2.0%, NR 1.6%. | Education: Some HS/HS graduate 8.4%, Some college 28.6%, College graduate 29.9%, Postgraduate training 33.1% | Overweight (25–29.9) 15.6%, Obesity I (30–34.9) 41.7%, Obesity II (35–39.9) 33.8%, Obesity III (>40) 8.8% | Anth, diet, PA |
| Rogers 2016[58] | USA | WL | Supported by an unrestricted gift from Google, Inc. | RJR was PI on a research grant from Weight Watchers International; BBG was PI on a research grant from Human Scale; JMJ received an honorarium for serving on the 2015 Scientific Advisory Board for Weight Watchers International, was the PI on a grant by Jawbon Inc., and was a co-investigator on research grants received from Weight Watchers International, Human Scale and Ethicon/Covidien, all awarded to the University of Pittsburgh | 39 | 39.5 (2.8) | 39.9 (11.5) | 79.5% | White/ Caucasian 71.8% | NR | NR | Anth, diet, PA |
| Ross 2016[59] | USA | WL | NIDDK | None to disclose | 80 | 33.0 (3.4) | 51.1 (11.1) | 86.2% | African American 3.8%, Asian 0%, Caucasian 83.7%, Hispanic 6.2%, Other 6.3% | NR | NR | Anth |
| Roth 2023[60] | Germany | WL | aidhere GmbH | KF and LR are employees of aidhere GmbH. NM is an employee and founder of aidhere GmbH. | 149 | 35.8 (3.2) | 43.4 (10.9) | 91.3% | NR | Education: Lower secondary 4.0%, Secondary 30.9%,Higher secondary 20.8%, Degree course 33.5%, Other 10.7% | NR | Anth, WB |
| Shapiro 2012[61] | USA | WL | NIH/NCI | JRS is the Scientific Director and TK is Product Manager (both receiving income from) of Santech, Inc., which is developing products related to the research described in this paper. ND has received consulting income from Santech for the analyses reported here. JFS, KC and KP are the co-founders and stockholders of Santech, Inc. | 170 | 32.2 (4.1) | 41.9 (11.8) | 65.0% | White 64.0% | Education 59.0% college | NR | Anth, PA, other |
| Shuger 2011[62] | USA | WL | Unrestricted research grant from BodyMedia, Inc | SNB receives book royalties (<$5,000/y) from Human Kinetics; honoraria for service on the Scientific/Medical Advisory Boards for Alere, Technogym, Santech, and Jenny Craig; and honoraria for lectures and consultations from scientific, educational, and lay groups | 197 | 33.3 (5.2) | 46.9 (10.8) | 81.7%% | White 66.8%, Black 32.1%, Other 1.0% | College degree 4yrs 77.2% | NR | Anth |
| Silina 2017[63] | Latvia | WL, RR | Latvian National Programme, and by the Doctoral Studies Grant of Riga Stradins University. | None to disclose | 123 | 32.0 (4.3) | 36.8 (4.2) | 52.5% | NR | NR | Hepatic steatosis on CT 38.5% | Anth, clin |
| Simpson 2020[64] | UK | WL | NIHR | SAS member of NIHR HTA programme, Clinical Evaluation and Trials funding committee from January 2017 to present, member of the NIHR PRP committee and the Chief Scientist Office Health Improvement, Protection and Services funding committee. EM member of the NIHR Public Health Research funding board from January 2016 to present | 109 | 37.6 (5.9) | 47.3 (10.7) | 69.7% | White British/Irish 84.3%, White other 5.6%, Indian 1.9%, Pakistani 1.9%, Chinese 0.9%, Other 5.6% | Education; Higher education 61.5%, Other 38.5%; Employment: Employee 78.9%, Self-employed 14.7%, Not employed 6.4%; SIMD quintile 1: most deprived 36.4%, 2 21.2%, 3 13.1%, 4 16.2%, 5 least deprived 13.1%. | Health problems within last 12 months (% yes): Heart disease 1.8%, Diabetes 5.5%, Depression 15.6%, Stroke 0.9%, Arthritis 11.0%, Hypertension 11.0%,High cholesterol 6.4%, Asthma 8.3%, COPD 1.8%, Back pain 16.5%, Other 11.0% | Anth, diet, PA, WB |
| Sniehotta 2019[65] | UK | WM | NPRI - Phase 4 | AB declares research funding in the last 5 years from EU Horizon 2020 COFUND; Medical Research Council. AB is statistical advisor to Cambwick Healthcare for a planned proof-of-concept medical device study | 288 | 30.9 (5.4) | 41.8 (11.5) | 77.5% | NR | Highest level of education: Bachelor’s degree or higher 53.1%, Post-16 qualification (e.g., HND/A-level) 22.6%, GCSE/O-level or below 26.4%. Employment: Full time employed 60.8%, Part-time employed 17.4%, Retired 9.4%, No paid employment 12.6%. Household income: <£10,000 3.9%, £10,001–£40,000 47.3%, £40,001–£70,000 35.4%, >£70,000 13.6% | NR | Anth, diet, MH, PA, WB, other |
| Steinberg 2013[66] | USA | WL | Duke University | None to disclose | 50 | 35.8 (6.1) | 38.3 (8.2) | 100.0% | Non-hispanic black 82.0%, Non-hispanic Other 18.0% | Employment: Employed 82%.Education: 4yr college degree or higher 64.0%, Less than a 4-year college degree 36.0%; Household income (US $) <40,000 32.0%, 40,000-69,999 36.0%, ≥ 70,000 32.0% | NR | Anth |
| Tate 2001[67] | USA | WL | Weight Risk Investigators Study Council, a research division of Knoll Pharmaceutical | NR | 91 | 29.0 (3.0) | 40.9 (10.7) | 89.0% | White 83.4% | Education: High School 9.0%, Some college 32.0%, College degree 32.0%, Graduate degree 27.0% | NR | Anth, diet, PA, other |
| Tate 2006[68] | USA | WL | SlimFast Nutrition Institute | None to disclose | 192 | 32.6 (3.5) | 49.2 (9.8) | 84.3% | Minority ethnic 10.7% | College graduate 54.7% | NR | Anth, diet, PA |
| Tate 2022[69] | USA | WL | NIDDK | DFT is a member of the Scientific Advisory Board for WW and Wondr Health | 31 | 35.1 (5.5) | 51.4 (11.2) | 72.0% | White 85.4%, Black 9.3%, Other 5.3% | Education: High School or less 25.2%, College 1–3 yrs 33.6%, College 4+ yrs 41.2%.Employed 75.8% | Diabetes 11.2%; Overweight (25.0 – 29.9) 19.1%, Obese Class I (30.0 – 34.9) 35.1%, Obese Class II (35.0 – 39.9) 25.6%, Obese Class III (40.0 – 50.0) 20.2% | Anth, diet, clin, PA, other |
| Teeriniemi 2018[70] | Finland | WL, RR | Grants from the Ministry of Social Affairs and Health, Finland, the Academy of Finland, the Sigrid Juselius Foundation, the Juho Vainio Foundation, the Finnish Foundation for Cardiovascular Research, and the Diabetes Research Foundation | None to disclose | 532 | 30.5 (2.1) | 46.0 (9.9) | 49.3% | NR | NR | Diabetes 2.4% | Anth, clin |
| Thomas 2017[71] | USA | WL | Weight Watchers International, Inc. | JGT, HAR, RRW, CCC, AKL, DSB report grants from Weight Watchers International during the conduct of the study. JGT reports personal fees from Applied VR, Covidien, LC, and Ketothrive Corp., all outside the submitted work. DSB reports personal fees from Covidien, LC, outside the submitted work. GDF reports salary support from Weight Watchers International during the conduct of the study | 271 | 33.9 (3.7) | 55.0 (11.6) | 77.5% | Race: American Indian 0.4%, Asian 1.2%, Black 5.9%, White 91.5%, Other 1.1%; Ethnicity: Hispanic or Latino 4.1%, Not Hispanic or Latino 95.9% | Education: High school or less 16.3%, Some college 28.9%, College or university degree 26.0%, Graduate degree 26.5% | NR | Anth, diet, PA |
| Thomas 2020[72] | USA | WL | National Heart, Lung, and Blood Institute and NIDDK, Grant | PWT received research consultant fees from Virtually Better, Inc. JGT and DSB received research support from Weight Watchers International, Inc. for other research projects during the completion of this study. Weight Watchers International, Inc. was not involved in the funding or execution of this study | 146 | 33.1 (4.9) | 58.3 (10.3) | 78.1% | Race: Asian 0.7%, Black 2.1%, White 94.5%, Other 2.7%; Ethnicity: Hispanic or Latino 1.4% | Education: High school or < 6.8%, Some college 29.5%, College or university degree 28.1%, Graduate degree 35.6% | NR | Anth, diet, MH, PA, WB |
| Turner-McGrievy 2011[73] | USA | WL | UNC Lineberger Comprehensive Cancer Center Population Sciences Award and the UNC Interdisciplinary Obesity Center | None to disclose | 96 | 32.5 (4.6) | 42.9 (11.2) | 75.0% | Race:Black 19.5%, White 76.5%, Other A4.0%; Ethnicity: Non-hispanic 100% | Education: College or less 45.0%, Graduate degree 55.0% | NR | Anth, diet, MH, PA, WB, other |
| Turner-McGrievy 2017[74] | USA | WL | National Cancer Institute of the NIH | AH and EM have formed a company, Bite Technologies, to market and sell a bite counting device. Clemson University owns a US patent for intellectual property known as “The Weight Watch”, USA, Patent No. 8310368, filed January 2009, granted November 13, 2012. Bite Technologies has licensed the method from Clemson University. Adam Hoover and Eric Muth receive royalty payments from bite counting device sales. The remaining authors do not have any conflicts of interest to declare. | 81 | 33.4 (5.2) | 48.1 (12.0) | 82.7% | Race: Black 16.0%, white 81.5%, other 2.5% | Education: high school or some college: 14.8%, college graduate, 45.7%, advanced degree, 39.5%; Occupation: no current employment 3.7%, service occupation 11.1%, technical, sales, administrative 16.0%, executive, managerial 6.2%, professional speciality 29.6%, retired 7.4%, other 25.9% | NR | Anth, diet, PA, other |
| van Genugten 2012[75] | NL | WM | The Netherlands Organisation for Health Research and Development | NR | 539 | 28.04 (1.94) | 47.8 (9.4) | 69.1% | NR | Education: Low 10.7%, Medium 50.2%, High 39,1% | BMI: % Normal weight (<25) 4.2%, % Overweight (25-30) 78.5%, % Obese (>30) 17.3% | Anth, diet, PA, other |
| Van Wier 2009,[76] 2011,[77]2012[78] | NL | WL | The Netherlands Organization for Health Research and Development | NR | 1386 | 29.6 (3.5) | 43.0 (8.6) | 33.0% | NR | Highly educated 60.4%. | Medication for comorbidities 17.7% | Anth, diet, PA |
| West 2016[79] | USA | WL | Partly by NIDDK | NR | 398 | 36.0 (6.0) | 48.4 (10.1) | 89.7%% | African American 24.1% | Education: High school/ Vocational Training 7.8%, Some college 18.3%, College degree 38.9%, Graduate/ Professional 34.9% | Obese (≥ 30 BMI) 81.9% | Anth, diet, PA, other |
| West 2019[80] | USA | WL | NIDDK | NR | 32 | 34.1 (5.5) | 47.2 (12.4) | 100% | White 78.1%, African American 18.8%, Other 3.1% | Education: Some college <4 years 9.0%, Vocational training post high school 3.0%, College degree 44.0%, Graduate/ professional 44.0% |  | Anth, other |
| Womble 2004[81] | USA | WL | North American Association for the Study of Obesity and NIH | NR | 47 | 33.4 (3.1) | 43.7 (10.2) | 100.0% | NR | NR | 1 participant in manual arm impaired glucose at baseline and developed T2DM during trial. They were excluded | Anth, clin, WB, other |
| Young 2017[82] | Australia | WM | Hunter Medical Research Institute’s Healthy Lifestyles Grant | NR | 92 | 33.0 (3.4) | 49.2 (10.1) | 0%% | NR | Achieved post-school qualification 80.0%. Currently employed 86.0%. Personal annual income  $0–$18,200 5.0%, $18,201–$37,000 8.0%, $37,001–$80,000 36.0%, $80,001–$180,000 37.0%, >$180,000 9.0%, don’t know/want to answer 5%. Socioeconomic status 1–2 (lowest) 3.0%, 3-4 13.0%, 5-6 41.0%, 7-8 30.0%, 9-10(highest) 12.0% | BMI category Healthy weight 1.0%, overweight 42.0%, obesity I 46.0%, obesity II 11.0% | Anth, clin, diet, PA |
| *Based on sample of 89** Poverty thresholds based on the US Census Bureau 2009 poverty thresholds | | | | | | | | | | | | |
| Anth, anthropometric; BMI, body mass index; CE, cost-effectiveness; COPD chronic obstructive pulmonary disease; CTER, Center for Therapeutic Effectiveness Research; DK don’t know; GED, General Education Development; GmbH, Gessellshaft mit beschränkter Haftung (German phrase for ‘company with limited ability’; HTA, Health Technology Assessment; IQR, inter quartile range; IMD, index of multiple deprivation; clin, clinical (i.e. HbA1c, blood pressure); MH, mental health; mHBCSS, mobile health behaviour change support system; N, number; NCI, National Cancer Institute; NIDDK, National Institute of Diabetes and Digestive and Kidney diseases; NIH, National Institutes of Health; NIHR, National Institute of Health Research, NL, Netherlands; NR, not reported; PA=Physical activity, PI, principal investigator; PRP, Policy Research Programme; RR, disease risk reduction; SES, socioeconomic status; SIMD, Scottish index of multiple deprivation; PA, physical activity; SNAP, supplemental nutrition assistance program; Scottish index of multiple deprivation; TIA, transient ischaemic attack; T2DM, Type 2 diabetes mellitus; WL, weight loss; WB, well-being; WM, weight maintenance; EASO, European Association for the study of obesity | | | | | | | | | | | | |

Bibliography

1. Apinaniz A, Cobos-Campos R, Saez de Lafuente-Morinigo A, Parraza N, Aizpuru F, Perez I, et al. Effectiveness of randomized controlled trial of a mobile app to promote healthy lifestyle in obese and overweight patients. Fam Pract. 2019 Nov 18;36(6):699-705. PMID: 31093681. doi: 10.1093/fampra/cmz020.

2. Backman DR, Kohatsu ND, Padovani AJ, Dao C, Ritley D, Fleuret JE, et al. Achieving weight loss through a community-based, telewellness programme: A randomised controlled trial. Health Educ J. 2022;82(1):82-94. PMID: 2020396037. doi: 10.1177/00178969221139234.

3. Beleigoli A, Andrade AQ, Diniz MF, Ribeiro AL. Personalized Web-Based Weight Loss Behavior Change Program With and Without Dietitian Online Coaching for Adults With Overweight and Obesity: Randomized Controlled Trial. J Med Internet Res. 2020 Nov 5;22(11):e17494. PMID: 33151151. doi: 10.2196/17494.

4. Berli C, Scholz U. Long-Term and Transfer Effects of an Action Control Intervention in Overweight Couples: A Randomized Controlled Trial Using Text Messages. Front Psychol. 2021;12:754488. PMID: 34899496. doi: 10.3389/fpsyg.2021.754488.

5. Bennett GG, Foley P, Levine E, Whiteley J, Askew S, Steinberg DM, et al. Behavioral treatment for weight gain prevention among black women in primary care practice: a randomized clinical trial. JAMA Intern Med. 2013 Oct 28;173(19):1770-7. PMID: 23979005. doi: 10.1001/jamainternmed.2013.9263.

6. Lanpher MG, Askew S, Bennett GG. Health Literacy and Weight Change in a Digital Health Intervention for Women: A Randomized Controlled Trial in Primary Care Practice. J Health Commun. 2016;21 Suppl 1(Suppl):34-42. PMID: 27043756. doi: 10.1080/10810730.2015.1131773.

7. Braun TD, Olson K, Panza E, Lillis J, Schumacher L, Abrantes AM, et al. Internalized weight stigma in women with class III obesity: A randomized controlled trial of a virtual lifestyle modification intervention followed by a mindful self-compassion intervention. Obes Sci Pract. 2022 Dec;8(6):816-27. PMID: 36483124. doi: 10.1002/osp4.616.

8. Burke LE, Sereika SM, Parmanto B, Bizhanova Z, Kariuki JK, Cheng J, et al. Effect of tailored, daily feedback with lifestyle self-monitoring on weight loss: The SMARTER randomized clinical trial. Obesity (Silver Spring). 2022 Jan;30(1):75-84. PMID: 34898011. doi: 10.1002/oby.23321.

9. Burke LE, Sereika SM, Bizhanova Z, Parmanto B, Kariuki J, Cheng J, et al. The Effect of Tailored, Daily, Smartphone Feedback to Lifestyle Self-Monitoring on Weight Loss at 12 Months: the SMARTER Randomized Clinical Trial. J Med Internet Res. 2022 Jul 5;24(7):e38243. PMID: 35787516. doi: 10.2196/38243.

10. Burke LE, Conroy MB, Sereika SM, Elci OU, Styn MA, Acharya SD, et al. The effect of electronic self-monitoring on weight loss and dietary intake: a randomized behavioral weight loss trial. Obesity (Silver Spring). 2011 Feb;19(2):338-44. PMID: 20847736. doi: 10.1038/oby.2010.208.

11. Burke LE, Styn MA, Sereika SM, Conroy MB, Ye L, Glanz K, et al. Using mHealth technology to enhance self-monitoring for weight loss: a randomized trial. Am J Prev Med. 2012 Jul;43(1):20-6. PMID: 22704741. doi: 10.1016/j.amepre.2012.03.016.

12. Turk MW, Elci OU, Wang J, Sereika SM, Ewing LJ, Acharya SD, et al. Self-monitoring as a mediator of weight loss in the SMART randomized clinical trial. Int J Behav Med. 2013 Dec;20(4):556-61. PMID: 22936524. doi: 10.1007/s12529-012-9259-9.

13. Conroy MB, Yang K, Elci OU, Gabriel KP, Styn MA, Wang J, et al. Physical activity self-monitoring and weight loss: 6-month results of the SMART trial. Med Sci Sports Exerc. 2011 Aug;43(8):1568-74. PMID: 21200337. doi: 10.1249/MSS.0b013e31820b9395.

14. Carter MC, Burley VJ, Nykjaer C, Cade JE. Adherence to a smartphone application for weight loss compared to website and paper diary: pilot randomized controlled trial. J Med Internet Res. 2013 Apr 15;15(4):e32. PMID: 23587561. doi: 10.2196/jmir.2283.

15. Collins CE, Morgan PJ, Hutchesson MJ, Callister R. Efficacy of standard versus enhanced features in a Web-based commercial weight-loss program for obese adults, part 2: randomized controlled trial. J Med Internet Res. 2013 Jul 22;15(7):e140. PMID: 23876832. doi: 10.2196/jmir.2626.

16. Collins CE, Morgan PJ, Hutchesson MJ, Oldmeadow C, Barker D, Callister R. Efficacy of Web-Based Weight Loss Maintenance Programs: A Randomized Controlled Trial Comparing Standard Features Versus the Addition of Enhanced Personalized Feedback over 12 Months. Behav Sci (Basel). 2017 Nov 8;7(4). PMID: 29117105. doi: 10.3390/bs7040076.

17. Conroy MB, McTigue KM, Bryce CL, Tudorascu D, Gibbs BB, Arnold J, et al. Effect of Electronic Health Record-Based Coaching on Weight Maintenance: A Randomized Trial. Ann Intern Med. 2019 Dec 3;171(11):777-84. PMID: 31711168. doi: 10.7326/M18-3337.

18. Dombrowski SU, McDonald M, van der Pol M, Grindle M, Avenell A, Carroll P, et al. Game of Stones: feasibility randomised controlled trial of how to engage men with obesity in text message and incentive interventions for weight loss. BMJ Open. 2020 Feb 25;10(2):e032653. PMID: 32102807. doi: 10.1136/bmjopen-2019-032653.

19. Duncan MJ, Fenton S, Brown WJ, Collins CE, Glozier N, Kolt GS, et al. Efficacy of a Multi-component m-Health Weight-loss Intervention in Overweight and Obese Adults: A Randomised Controlled Trial. Int J Environ Res Public Health. 2020 Aug 26;17(17). PMID: 32859100. doi: 10.3390/ijerph17176200.

20. Dunn CG, Turner-McGrievy GM, Wilcox S, Hutto B. Dietary Self-Monitoring Through Calorie Tracking but Not Through a Digital Photography App Is Associated with Significant Weight Loss: The 2SMART Pilot Study-A 6-Month Randomized Trial. J Acad Nutr Diet. 2019 Sep;119(9):1525-32. PMID: 31155474. doi: 10.1016/j.jand.2019.03.013.

21. Eisenhauer CM, Brito F, Kupzyk K, Yoder A, Almeida F, Beller RJ, et al. Mobile health assisted self-monitoring is acceptable for supporting weight loss in rural men: a pragmatic randomized controlled feasibility trial. BMC Public Health. 2021 Aug 18;21(1):1568. PMID: 34407782. doi: 10.1186/s12889-021-11618-7.

22. Falkenhain K, Locke SR, Lowe DA, Reitsma NJ, Lee T, Singer J, et al. Keyto app and device versus WW app on weight loss and metabolic risk in adults with overweight or obesity: A randomized trial. Obesity (Silver Spring). 2021 Oct;29(10):1606-14. PMID: 34124856. doi: 10.1002/oby.23242.

23. Gemesi K, Winkler S, Schmidt-Tesch S, Schederecker F, Hauner H, Holzapfel C. Efficacy of an app-based multimodal lifestyle intervention on body weight in persons with obesity: results from a randomized controlled trial. Int J Obes (Lond). 2024 Jan;48(1):118-26. PMID: 38017117. doi: 10.1038/s41366-023-01415-0.

24. Gold BC, Burke S, Pintauro S, Buzzell P, Harvey-Berino J. Weight loss on the web: A pilot study comparing a structured behavioral intervention to a commercial program. Obesity (Silver Spring). 2007 Jan;15(1):155-64. PMID: 17228043. doi: 10.1038/oby.2007.520.

25. Haapala I, Barengo NC, Biggs S, Surakka L, Manninen P. Weight loss by mobile phone: a 1-year effectiveness study. Public Health Nutr. 2009 Dec;12(12):2382-91. PMID: 19323865. doi: 10.1017/S1368980009005230.

26. Hageman PA, Pullen CH, Hertzog M, Pozehl B, Eisenhauer C, Boeckner LS. Web-Based Interventions Alone or Supplemented with Peer-Led Support or Professional Email Counseling for Weight Loss and Weight Maintenance in Women from Rural Communities: Results of a Clinical Trial. J Obes. 2017;2017:1602627. PMID: 28480078. doi: 10.1155/2017/1602627.

27. Adachi Y, Sato C, Yamatsu K, Ito S, Adachi K, Yamagami T. A randomized controlled trial on the long-term effects of a 1-month behavioral weight control program assisted by computer tailored advice. Behav Res Ther. 2007 Mar;45(3):459-70. PMID: 16713991. doi: 10.1016/j.brat.2006.03.017.

28. Christensen JR, Hesseldal L, Olesen TB, Olsen MH, Jakobsen PR, Laursen DH, et al. Long-term weight loss in a 24-month primary care-anchored telehealth lifestyle coaching program: Randomized controlled trial. J Telemed Telecare. 2022 Dec;28(10):764-70. PMID: 36346936. doi: 10.1177/1357633X221123411.

29. Hutchesson MJ, Callister R, Morgan PJ, Pranata I, Clarke ED, Skinner G, et al. A Targeted and Tailored eHealth Weight Loss Program for Young Women: The Be Positive Be Healthe Randomized Controlled Trial. Healthcare (Basel). 2018 May 2;6(2). PMID: 29724054. doi: 10.3390/healthcare6020039.

30. Jane M, Hagger M, Foster J, Ho S, Kane R, Pal S. Effects of a weight management program delivered by social media on weight and metabolic syndrome risk factors in overweight and obese adults: A randomised controlled trial. PLoS One. 2017;12(6):e0178326. PMID: 28575048. doi: 10.1371/journal.pone.0178326.

31. Jane M, Foster J, Hagger M, Ho S, Kane R, Pal S. Psychological effects of belonging to a Facebook weight management group in overweight and obese adults: Results of a randomised controlled trial. Health Soc Care Community. 2018 May 18. PMID: 29774616. doi: 10.1111/hsc.12584.

32. Joseph RP, Todd M, Ainsworth BE, Vega-Lopez S, Adams MA, Hollingshead K, et al. Smart Walk: A Culturally Tailored Smartphone-Delivered Physical Activity Intervention for Cardiometabolic Risk Reduction among African American Women. Int J Environ Res Public Health. 2023 Jan 5;20(2). PMID: 36673756. doi: 10.3390/ijerph20021000.

33. Kempf K, Rohling M, Martin S, Schneider M. Telemedical coaching for weight loss in overweight employees: a three-armed randomised controlled trial. BMJ Open. 2019 Apr 11;9(4):e022242. PMID: 30975666. doi: 10.1136/bmjopen-2018-022242.

34. Kempf K, Rohling M, Stichert M, Fischer G, Boschem E, Konner J, et al. Telemedical Coaching Improves Long-Term Weight Loss in Overweight Persons: A Randomized Controlled Trial. Int J Telemed Appl. 2018;2018:7530602. PMID: 30271433. doi: 10.1155/2018/7530602.

35. Keshavarz M, Senechal M, Bouchard DR. Online Circuit Training Increases Adherence to Physical Activity: A Randomized Controlled Trial of Men with Obesity. Med Sci Sports Exerc. 2023 Dec 1;55(12):2308-15. PMID: 37535330. doi: 10.1249/MSS.0000000000003270.

36. Kharmats AY, Wang C, Fuentes L, Hu L, Kline T, Welding K, et al. Monday-focused tailored rapid interactive mobile messaging for weight management 2 (MTRIMM2): results from a randomized controlled trial. Mhealth. 2022;8:1. PMID: 35178432. doi: 10.21037/mhealth-21-3.

37. Kim M, Kim Y, Go Y, Lee S, Na M, Lee Y, et al. Multidimensional Cognitive Behavioral Therapy for Obesity Applied by Psychologists Using a Digital Platform: Open-Label Randomized Controlled Trial. JMIR Mhealth Uhealth. 2020 Apr 30;8(4):e14817. PMID: 32352391. doi: 10.2196/14817.

38. Kohl J, Brame J, Centner C, Wurst R, Fuchs R, Sehlbrede M, et al. Effects of a Web-Based Lifestyle Intervention on Weight Loss and Cardiometabolic Risk Factors in Adults With Overweight and Obesity: Randomized Controlled Clinical Trial. J Med Internet Res. 2023 Jun 27;25:e43426. PMID: 37368484. doi: 10.2196/43426.

39. Kurtzman GW, Day SC, Small DS, Lynch M, Zhu J, Wang W, et al. Social Incentives and Gamification to Promote Weight Loss: The LOSE IT Randomized, Controlled Trial. J Gen Intern Med. 2018 Oct;33(10):1669-75. PMID: 30003481. doi: 10.1007/s11606-018-4552-1.

40. Laing BY, Mangione CM, Tseng CH, Leng M, Vaisberg E, Mahida M, et al. Effectiveness of a smartphone application for weight loss compared with usual care in overweight primary care patients: a randomized, controlled trial. Ann Intern Med. 2014 Nov 18;161(10 Suppl):S5-12. PMID: 25402403. doi: 10.7326/M13-3005.

41. LaRose JG, Leahey TM, Lanoye A, Bean MK, Fava JL, Tate DF, et al. Effect of a Lifestyle Intervention on Cardiometabolic Health Among Emerging Adults: A Randomized Clinical Trial. JAMA Netw Open. 2022 Sep 1;5(9):e2231903. PMID: 36121656. doi: 10.1001/jamanetworkopen.2022.31903.

42. Leahey TM, Subak LL, Fava J, Schembri M, Thomas G, Xu X, et al. Benefits of adding small financial incentives or optional group meetings to a web-based statewide obesity initiative. Obesity (Silver Spring). 2015 Jan;23(1):70-6. PMID: 25384463. doi: 10.1002/oby.20937.

43. Leahey TM, Fava JL, Seiden A, Fernandes D, Doyle C, Kent K, et al. A randomized controlled trial testing an Internet delivered cost-benefit approach to weight loss maintenance. Prev Med. 2016 Nov;92:51-7. PMID: 27095323. doi: 10.1016/j.ypmed.2016.04.013.

44. Little P, Stuart B, Hobbs FR, Kelly J, Smith ER, Bradbury KJ, et al. An internet-based intervention with brief nurse support to manage obesity in primary care (POWeR+): a pragmatic, parallel-group, randomised controlled trial. Lancet Diabetes Endocrinol. 2016 Oct;4(10):821-8. PMID: 27474214. doi: 10.1016/S2213-8587(16)30099-7.

45. Little P, Stuart B, Hobbs FR, Kelly J, Smith ER, Bradbury KJ, et al. Randomised controlled trial and economic analysis of an internet-based weight management programme: POWeR+ (Positive Online Weight Reduction). Health Technol Assess. 2017 Jan;21(4):1-62. PMID: 28122658. doi: 10.3310/hta21040.

46. Markkanen JO, Oikarinen N, Savolainen MJ, Merikallio H, Nyman V, Salminen V, et al. Mobile health behaviour change support system as independent treatment tool for obesity: a randomized controlled trial. Int J Obes (Lond). 2024 Mar;48(3):376-83. PMID: 38062218. doi: 10.1038/s41366-023-01426-x.

47. McConnon A, Kirk SF, Cockroft JE, Harvey EL, Greenwood DC, Thomas JD, et al. The Internet for weight control in an obese sample: results of a randomised controlled trial. BMC Health Serv Res. 2007 Dec 19;7:206. PMID: 18093289. doi: 10.1186/1472-6963-7-206.

48. Morgan PJ, Lubans DR, Collins CE, Warren JM, Callister R. The SHED-IT randomized controlled trial: evaluation of an Internet-based weight-loss program for men. Obesity (Silver Spring). 2009 Nov;17(11):2025-32. PMID: 19343018. doi: 10.1038/oby.2009.85.

49. Morgan PJ, Lubans DR, Collins CE, Warren JM, Callister R. 12-month outcomes and process evaluation of the SHED-IT RCT: an internet-based weight loss program targeting men. Obesity (Silver Spring). 2011 Jan;19(1):142-51. PMID: 20523304. doi: 10.1038/oby.2010.119.

50. Morgan PJ, Callister R, Collins CE, Plotnikoff RC, Young MD, Berry N, et al. The SHED-IT community trial: a randomized controlled trial of internet- and paper-based weight loss programs tailored for overweight and obese men. Ann Behav Med. 2013 Apr;45(2):139-52. PMID: 23129021. doi: 10.1007/s12160-012-9424-z.

51. Blomfield RL, Collins CE, Hutchesson MJ, Young MD, Jensen ME, Callister R, et al. Impact of self-help weight loss resources with or without online support on the dietary intake of overweight and obese men: the SHED-IT randomised controlled trial. Obes Res Clin Pract. 2014 Sep-Oct;8(5):e476-87. PMID: 25263837. doi: 10.1016/j.orcp.2013.09.004.

52. Mueller J, Richards R, Jones RA, Whittle F, Woolston J, Stubbings M, et al. Supporting Weight Management during COVID-19: A Randomized Controlled Trial of a Web-Based, ACT-Based, Guided Self-Help Intervention. Obes Facts. 2022;15(4):550-9. PMID: 35417910. doi: 10.1159/000524031.

53. Mueller J, Richards R, Jones RA, Whittle F, Woolston J, Stubbings M, et al. Supporting Weight Management during COVID-19 (SWiM-C): twelve-month follow-up of a randomised controlled trial of a web-based, ACT-based, guided self-help intervention. Int J Obes (Lond). 2023 Jan;47(1):51-9. PMID: 36369513. doi: 10.1038/s41366-022-01232-x.

54. Olson R, Wipfli B, Thompson SV, Elliot DL, Anger WK, Bodner T, et al. Weight Control Intervention for Truck Drivers: The SHIFT Randomized Controlled Trial, United States. Am J Public Health. 2016 Sep;106(9):1698-706. PMID: 27463067. doi: 10.2105/AJPH.2016.303262.

55. Wipfli B, Hanson G, Anger K, Elliot DL, Bodner T, Stevens V, et al. Process Evaluation of a Mobile Weight Loss Intervention for Truck Drivers. Saf Health Work. 2019 Mar;10(1):95-102. PMID: 30949387. doi: 10.1016/j.shaw.2018.08.002.

56. Patel ML, Hopkins CM, Brooks TL, Bennett GG. Comparing Self-Monitoring Strategies for Weight Loss in a Smartphone App: Randomized Controlled Trial. JMIR Mhealth Uhealth. 2019 Feb 28;7(2):e12209. PMID: 30816851. doi: 10.2196/12209.

57. Patrick K, Calfas KJ, Norman GJ, Rosenberg D, Zabinski MF, Sallis JF, et al. Outcomes of a 12-month web-based intervention for overweight and obese men. Ann Behav Med. 2011 Dec;42(3):391-401. PMID: 21822750. doi: 10.1007/s12160-011-9296-7.

58. Rogers RJ, Lang W, Barone Gibbs B, Davis KK, Burke LE, Kovacs SJ, et al. Applying a technology-based system for weight loss in adults with obesity. Obes Sci Pract. 2016 Mar;2(1):3-12. PMID: 27812375. doi: 10.1002/osp4.18.

59. Ross KM, Wing RR. Impact of newer self-monitoring technology and brief phone-based intervention on weight loss: A randomized pilot study. Obesity (Silver Spring). 2016 Aug;24(8):1653-9. PMID: 27367614. doi: 10.1002/oby.21536.

60. Roth L, Ordnung M, Forkmann K, Mehl N, Horstmann A. A randomized-controlled trial to evaluate the app-based multimodal weight loss program zanadio for patients with obesity. Obesity (Silver Spring). 2023 May;31(5):1300-10. PMID: 37140392. doi: 10.1002/oby.23744.

61. Shapiro JR, Koro T, Doran N, Thompson S, Sallis JF, Calfas K, et al. Text4Diet: a randomized controlled study using text messaging for weight loss behaviors. Prev Med. 2012 Nov;55(5):412-7. PMID: 22944150. doi: 10.1016/j.ypmed.2012.08.011.

62. Shuger SL, Barry VW, Sui X, McClain A, Hand GA, Wilcox S, et al. Electronic feedback in a diet- and physical activity-based lifestyle intervention for weight loss: a randomized controlled trial. Int J Behav Nutr Phys Act. 2011 May 18;8:41. PMID: 21592351. doi: 10.1186/1479-5868-8-41.

63. Silina V, Tessma MK, Senkane S, Krievina G, Bahs G. Text messaging (SMS) as a tool to facilitate weight loss and prevent metabolic deterioration in clinically healthy overweight and obese subjects: a randomised controlled trial. Scand J Prim Health Care. 2017 Sep;35(3):262-70. PMID: 28812403. doi: 10.1080/02813432.2017.1358435.

64. Simpson SA, Matthews L, Pugmire J, McConnachie A, McIntosh E, Coulman E, et al. An app-, web- and social support-based weight loss intervention for adults with obesity: the HelpMeDoIt! feasibility RCT. Public Health Research. 2020;8(3). doi: <https://dx.doi.org/10.3310/phr08030>.

65. Sniehotta FF, Evans EH, Sainsbury K, Adamson A, Batterham A, Becker F, et al. Behavioural intervention for weight loss maintenance versus standard weight advice in adults with obesity: A randomised controlled trial in the UK (NULevel Trial). PLoS Med. 2019 May;16(5):e1002793. PMID: 31063507. doi: 10.1371/journal.pmed.1002793.

66. Steinberg DM, Levine EL, Askew S, Foley P, Bennett GG. Daily text messaging for weight control among racial and ethnic minority women: randomized controlled pilot study. J Med Internet Res. 2013 Nov 18;15(11):e244. PMID: 24246427. doi: 10.2196/jmir.2844.

67. Tate DF, Wing RR, Winett RA. Using Internet technology to deliver a behavioral weight loss program. JAMA. 2001 Mar 7;285(9):1172-7. PMID: 11231746. doi: 10.1001/jama.285.9.1172.

68. Tate DF, Jackvony EH, Wing RR. A randomized trial comparing human e-mail counseling, computer-automated tailored counseling, and no counseling in an Internet weight loss program. Arch Intern Med. 2006;166(15):1620-5.

69. Tate DF, Lutes LD, Bryant M, Truesdale KP, Hatley KE, Griffiths Z, et al. Efficacy of a Commercial Weight Management Program Compared With a Do-It-Yourself Approach: A Randomized Clinical Trial. JAMA Netw Open. 2022 Aug 1;5(8):e2226561. PMID: 35972742. doi: 10.1001/jamanetworkopen.2022.26561.

70. Teeriniemi AM, Salonurmi T, Jokelainen T, Vahanikkila H, Alahaivala T, Karppinen P, et al. A randomized clinical trial of the effectiveness of a Web-based health behaviour change support system and group lifestyle counselling on body weight loss in overweight and obese subjects: 2-year outcomes. J Intern Med. 2018 Nov;284(5):534-45. PMID: 29974563. doi: 10.1111/joim.12802.

71. Thomas JG, Raynor HA, Bond DS, Luke AK, Cardoso CC, Foster GD, et al. Weight loss in Weight Watchers Online with and without an activity tracking device compared to control: A randomized trial. Obesity (Silver Spring). 2017 Jun;25(6):1014-21. PMID: 28437597. doi: 10.1002/oby.21846.

72. Thomas JG, Goldstein CM, Bond DS, Hadley W, Tuerk PW. Web-based virtual reality to enhance behavioural skills training and weight loss in a commercial online weight management programme: The Experience Success randomized trial. Obes Sci Pract. 2020 Dec;6(6):587-95. PMID: 33354337. doi: 10.1002/osp4.451.

73. Turner-McGrievy G, Tate D. Tweets, Apps, and Pods: Results of the 6-month Mobile Pounds Off Digitally (Mobile POD) randomized weight-loss intervention among adults. J Med Internet Res. 2011 Dec 20;13(4):e120. PMID: 22186428. doi: 10.2196/jmir.1841.

74. Turner-McGrievy GM, Wilcox S, Boutte A, Hutto BE, Singletary C, Muth ER, et al. The Dietary Intervention to Enhance Tracking with Mobile Devices (DIET Mobile) Study: A 6-Month Randomized Weight Loss Trial. Obesity (Silver Spring). 2017 Aug;25(8):1336-42. PMID: 28600833. doi: 10.1002/oby.21889.

75. van Genugten L, van Empelen P, Boon B, Borsboom G, Visscher T, Oenema A. Results from an online computer-tailored weight management intervention for overweight adults: randomized controlled trial. J Med Internet Res. 2012 Mar 14;14(2):e44. PMID: 22417813. doi: 10.2196/jmir.1901.

76. van Wier MF, Ariens GA, Dekkers JC, Hendriksen IJ, Smid T, van Mechelen W. Phone and e-mail counselling are effective for weight management in an overweight working population: a randomized controlled trial. BMC Public Health. 2009 Jan 9;9:6. PMID: 19134171. doi: 10.1186/1471-2458-9-6.

77. van Wier MF, Dekkers JC, Hendriksen IJ, Heymans MW, Ariens GA, Pronk NP, et al. Effectiveness of phone and e-mail lifestyle counseling for long term weight control among overweight employees. J Occup Environ Med. 2011 Jun;53(6):680-6. PMID: 21654441. doi: 10.1097/JOM.0b013e31821f2bbb.

78. van Wier MF, Dekkers JC, Bosmans JE, Heymans MW, Hendriksen IJ, Pronk NP, et al. Economic evaluation of a weight control program with e-mail and telephone counseling among overweight employees: a randomized controlled trial. Int J Behav Nutr Phys Act. 2012 Sep 11;9:112. PMID: 22967224. doi: 10.1186/1479-5868-9-112.

79. West DS, Harvey JR, Krukowski RA, Prewitt TE, Priest J, Ashikaga T. Do individual, online motivational interviewing chat sessions enhance weight loss in a group-based, online weight control program? Obesity (Silver Spring). 2016 Nov;24(11):2334-40. PMID: 27616628. doi: 10.1002/oby.21645.

80. West DS, Stansbury M, Krukowski RA, Harvey J. Enhancing group-based internet obesity treatment: A pilot RCT comparing video and text-based chat. Obes Sci Pract. 2019 Dec;5(6):513-20. PMID: 31890241. doi: 10.1002/osp4.371.

81. Womble LG, Wadden TA, McGuckin BG, Sargent SL, Rothman RA, Krauthamer-Ewing ES. A randomized controlled trial of a commercial internet weight loss program. Obes Res. 2004 Jun;12(6):1011-8. PMID: 15229342. doi: 10.1038/oby.2004.124.

82. Young MD, Callister R, Collins CE, Plotnikoff RC, Aguiar EJ, Morgan PJ. Efficacy of a gender-tailored intervention to prevent weight regain in men over 3 years: A weight loss maintenance RCT. Obesity (Silver Spring). 2017 Jan;25(1):56-65. PMID: 27925437. doi: 10.1002/oby.21696.
