# Supplementary material for: Evaluation of the Aspects of Digital Interventions That Successfully Support Weight Loss: Systematic Review With Component Network Meta-Analysis
Source: J Med Internet Res. 2025 May 22;27:e65443. doi: 10.2196/65443 (PMC12141966; doi:10.2196/65443)
Supplement: Multimedia Appendix 4 [file jmir_v27i1e65443_app4.docx]

**Multimedia Appendix 4.** Trials or trial arms not carried forward to full synthesis.

Trials and trial arms in this table were not carried forward to full synthesis because they were either notionally digital, or compared a predominantly digital intervention with a predominantly face to face comparator.

| **Author, date** | **Trial arm label** | **Overview of trial arm** | **Trial arm category** |
| --- | --- | --- | --- |
| Allen2013[1] | Smartphone | App to monitor food intake, exercise, and weight. Daily calorie budget calculated | DO |
| Allen2013[1] | Less intensive counselling and smartphone | 1-hour face-to-face counselling sessions from the nutritionist twice during the first month and then monthly from two to six months with smartphone app | ND |
| Allen2013[1] | Intensive counselling | 1-hour face-to-face counselling sessions from a nutritionist coach weekly for the first month and biweekly for the second through sixth month | F2F |
| Allen2013[1] | Intensive counselling and smartphone | 1-hour face-to-face counselling sessions from a nutritionist coach weekly for the first month and biweekly for the second through sixth month with smartphone app | F2F |
| Almeida2015[2] | INCENT | Daily tailored emails. Access to website for video explanations of exercises, links to recipes, discussion forums, and links to an electronic fitness advisor and self-monitoring of weight and PA. Monetary incentive based on percentage weight loss | DO |
| Almeida2015[2] | LMW | Four newsletters providing exercise and diet information and quarterly 1 hour group sessions | F2F |
| Barnes2014,2017[3, 4] | MIC | Five sessions (in-person at weeks 6, 12, by phone at weeks 3, 9) incorporating motivational interviewing strategies. Access to website for weekly weight loss goals, information, and self-monitoring and educational LEARN manual | ND |
| Barnes2014,2017[3, 4] | NPC | Five face-to-face psychoeducation sessions. Website access but limited to setting weight loss/calorie goals only. LEARN manual | F2F |
| Barnes2014,2017[3, 4] | Usual Care | Standard contact with PC providers only | Tau |
| Bergman2018[5] | Intervention group | Portable treadmill workstation treadmill, instructed to use at least 1 hour/day. Four emails with information and reminders to exercise | ND |
| Bergman2018[5] | Control group | Work as usual at their desk | TAU |
| Brindal2016,2018[6, 7] | Static app | PMRP (3 main meals, 3 snacks) with support from a trained consultant, had access to a dietitian telephone hotline, and static app – information-based app | ND |
| Brindal2016,2018[6, 7] | Analyst app | PMRP (3 main meals, 3 snacks) with support from a trained consultant, had access to a dietitian telephone hotline and analyst app – interactive self-monitoring tools, motivational messages, and reminders in addition to the program information | ND |
| Burke2011,[8]Burke2012,[9] Turk2013,[10]Conroy2011[11] | PR | Face-to-face group sessions plus standard paper diaries for recording food intake | F2F |
| Butryn2020[12] | LM | Weekly individual 15 min phone calls with counsellor and weekly one-way text messages. Daily self-monitoring of weight, diet, and PA | ND |
| Butryn2020[12] | LM + share | As above plus counsellors have access to self-monitoring device data which were used to set goals and provide feedback during the calls | ND |
| Gerber2013[13] | Video telehealth maintenance | 3 channels of video content providing information, instructions, reminders and stories for healthy eating and PA across internet enabled DVR | DO |
| Gerber2013[13] | Telephone support maintenance | Monthly telephone support calls, emails and Listserv communication | F2F |
| Godino2016[14] | SMART | Delivered across 6 modalities: Facebook, three study-designed mobile app text messaging, emails, a website with blog posts, and technology-mediated communication with a health coach (up to ten brief [5–15 min] interactions. Intervention participants were instructed to use at least one or more modalities a minimum of 5 times per week throughout the 24 months of the intervention | DO |
| Godino2016[14] | Control | Website for information, quarterly newsletters via email | ND |
| Griffith2023[15] | Weight-loss condition | 2 x 45min group face-to-face sessions (PA and Mighty Men curriculum), 3 x SMS/week, educational resources, self-monitoring | DL |
| Griffith2023[15] | Control condition | One group face-to-face PA session, educational resources, self-monitoring | ND |
| Hernandez-Reyes2020[16],Hernandez-Reyes2020[17] | Push notifications | Prescription of PA and diet via face-to-face consultation plus personalized health-related and motivational messages | ND |
| Hernandez-Reyes2020[16], Hernandez-Reyes2020[17] | No push notifications | Prescription of PA and diet via face-to-face consultation without messages | F2F |
| Jakicic2016[18] | SBWI | Weekly group-based sessions first 6 months, monthly 7-24 months. Individual monthly phone calls and weekly text messages months 7-24 plus access to website for education, self-monitoring of PA and diet | ND |
| Jakicic2016[18] | EWLI | Weekly group-based sessions first 6 months, monthly 7-24 months. Access to website only used to access education materials and wearable technology was provided along with a web-based interface to monitor physical activity and diet | F2F |
| Johnston2013[19] | WW | Weekly group face-to-face meetings. Website access and mobile app | ND |
| Johnston2013[19] | Self help | Publicly available printed material and online resources. Free 6-month membership to WW upon completion of the trial | WL |
| Karmali2020[20] | Control | 6 x health education sessions plus nine, 20-minute, one-on-one, telephone-based coaching sessions (3/month for 3 months) | ND |
| Karmali2020[20] | Intervention | 6 x online health education sessions then matched with a CPCC to create 9 one on one telephone based coaching sessions 3/month for 3 months | ND |
| Kim2015[21] | Text message group | Text messages were sent to the intervention group 3 times a week in the morning and consisted of “goal setting and behaviour change,” “education and tips for nutrition,” and “exercise and get more active” themes, plus 6-8 40 min educational group sessions plus printed materials | ND |
| Kim2015[21] | Control group | 6-8 40 min educational group sessions plus printed materials | F2F |
| Kurtzman2018[22] | Gamification with PCP sharing | Weekly gamification intervention with daily contact for 36 weeks that used points and levels to enhance collaborative social incentives plus data sharing with PCP | ND |
| LaRose2019[23] | Web based BWL | 1x 60 min face-to-face group session, access to website, 12x weekly online lessons, email reminders and e-coaching, self-monitoring via app, weekly emails | DO |
| LaRose2019[23] | Hybrid | 1 x 60 min face-to-face plus 12 optional community-based sessions, self-monitoring via app, weekly emails | ND |
| LaRose2019[23] | F2F BWL | 12 weekly face-to-face 60 min group sessions, 5 bi-weekly face-to-face individual check in sessions, self-monitoring using app or paper. Total 820 mins of face-to-face contact | F2F |
| Leahey2015[24] | SIG arm | Weekly multi-media videos, self-monitoring platform where participants submitted their daily weight, calorie, and activity information and received weekly, automated feedback on progress, automated weekly reminders to self-monitor plus 12 optional weekly sessions were led by dietitians or exercise physiologists | ND |
| Lin2014[25] | Intervention | 3 x 90 min face-to-face sessions during weeks 1, 2 and, plus 5 individual 20-30 mins coaching calls at week 3, 9 11, 17 and 20 plus daily text message | ND |
| Lin2014[25] | Control | Brief face-to-face information session plus a similar version of the lifestyle intervention for 4 months after completing the final data collect | WL |
| Little2016,2017[26, 27] | POWER + Face-to-face | 3 scheduled (and 4 optional) face-to-face nurse support sessions | ND |
| Madrona Marcos2019[28] | G1 | Face-to-face meetings with trained nurse | F2F |
| Madrona Marcos2019[28] | G2 | Face-to-face meetings with trained nurse in small groups plus digital platform support for self-monitoring | DL |
| Melchart2017[29] | IHM | In the 1^st^ 3 months, 3 full face-to-face introduction days. 2-hour weekly training sessions plus telephone or email support with trained coach over following 9 months. Access to web-based platform throughout | ND |
| Melchart2017[29] | UC | Usual care information only (written handout) | Tau |
| Micco2007[30] | I | Weekly online meetings in first 6 months then bi-weekly plus VTrim study website | DO |
| Micco2007[30] | IPS | Access to VTrim study website plus 1/month in person group meetings | ND |
| Nicklas2014[31] | SRI + DIET + EX | 5-month weight loss intervention. 2 meals/day provided and supervised exercise program 4 days/wk plus self-monitoring tools | ND |
| Nicklas2014[31] | DIET + EX | 5-month weight loss intervention. 2 meals/day provided and supervised exercise program 4 days/wk | F2F |
| Pellegrini2011[32] | SBWL | 3 x face-to-face meetings/month (2 group and 1 individual), diet and exercise guidance. Self-monitoring via paper diaries | F2F |
| Pellegrini2011[32] | SBWL + TECH | 3 x face-to-face meetings/month (2 group and 1 individual), diet and exercise guidance. Self-monitoring via technology system (BodyMedia Fit) and website | ND |
| Pellegrini2011[32] | TECH only | Same SWBL content as above arms but lessons emailed weekly. Use of technology system and website for self-monitoring. I x hour weight loss information session, plus 1 x phone call/ month from counsellor | DL |
| Pinto2013[33] | BWL | Weekly group 60-min sessions for first 24 weeks and bi-weekly for following 24 weeks. Dietary and PA goals, plus pedometer for self-monitoring | ND |
| Pinto2013[33] | WW | Vouchers to attend 48 weeks of WW plus access to online resources and tracking tools | F2F |
| Pinto2013[33] | Combined | 12 weeks of BWL plus 36 weeks of WW plus tracking tools | ND |
| Shuger2011[34] | GWL | 14 x group face-to-face sessions in first 4 months followed by 6 individual phone calls over next 5 months | F2F |
| Shuger2011[34] | GWL + SWA | 14 x group face-to-face sessions in first 4 months followed by 6 individual phone calls over next 5 months plus SenseWear platform consisting of the armband, a real-time wristwatch display, and access to a personalized Weight Management Solutions web account | ND |
| Spring2017[35] | Standard STND | Weekly 90 min face-to-face group sessions in first 8 weeks, plus 30 min guided walking exercise post session. Plus, calorie counting and paper self-monitoring diaries | F2F |
| Spring2017[35] | TECH | Weekly 90 min face-to-face group sessions in first 8 weeks, plus 30 min guided walking exercise post session. Plus, calorie counting and paper self-monitoring diaries plus smartphone app and pedometer for self-monitoring and communication. 2–4 personalized messages/week for 6 months | ND |
| Spring2017[35] | SELF | 1 x 60 min group session plus calorie counting book and daily self-monitoring paper diaries | F2F |
| Sullivan2013[36] | Second Life only | Second Life virtual clinic and web-based platform for weight loss and maintenance (monthly for 6 months and then bimonthly for 3 months) | DO |
| Sullivan2013[36] | Face-to-face and Second Life | Face-to-face clinics for weight loss (3 months) followed by Second Life (virtual clinic) for maintenance (6 months) using web-based platform | ND |
| Svetkey2015,[37]Lin2018[38] | PC | 6 x weekly group face-to-face sessions followed by monthly phone calls. Self-monitoring via smartphone app | ND |
| Svetkey2015,[37]Lin2018[38] | CP | Intervention delivery and self-monitoring via smartphone app | DO |
| Svetkey2015,[37]Lin2018[38] | Control group | 3 x handouts on healthy eating and physical activity from the Eat Smart Move More NC program | Tau? |
| Tarraga Marcos2017[39] | Group 1 | Obesity motivational intervention group with previously trained nurse. Hour-long motivational group intervention every two weeks from weeks 1 to 12, then monthly from weeks 13 to 32 | F2F |
| Tarraga Marcos2017[39] | Group 2 | Lower intensity consultation (15 days, 1 month, 3 months, 6 months and one year), non-motivational group, with digital platform support | ND |
| Tarraga Marcos2017[39] | Group 3 | Usual care | Tau |
| Tate2022[40] | Commercial weight management programme group | Weekly commercial weight management program workshops in the community, with smartphone app support. | ND |
| Tate2022[40] | Do-it-yourself | Self-directed weight management of participant’s choice | ND |
| Teeriniemi2018[41] | CBT+HBCSS | As CBT arm plus access to HBCSS for weekly tasks, access to information and tools of the system, such as self-recording of weight and physical activity | ND |
| Teeriniemi2018[41] | SHG | Self-help guidance counselling, based on two 90 min group counselling sessions, face to face | F2F |
| Teeriniemi2018[41] | CBT | Eight 90-min sessions in groups of eight to nine participants, fortnightly. | F2F |
| Thomas2019[42] | SMART | Smartphone-based treatment with online lessons, self-monitoring, and feedback and monthly weigh-ins | DL |
| Thomas2019[42] | GROUP | Group-based treatment with meetings weekly for 6 months, bi-weekly for 6 months, and monthly for 6-months and self-monitoring via paper diaries with written feedback | F2F |
| Thomas2019[42] | CONTROL | Monthly weigh-ins with interventionists to evaluate progress towards goals. Self-monitoring with paper diaries. | F2F |
| Thompson2014[43] | Experimental group | Received an accelerometer and counselling using Go4Life educational material for 24 weeks and accelerometer alone for the next 24 weeks | ND |
| Thompson2014[43] | Control group | No intervention for the first 24 weeks, then received an accelerometer and Go4Life based counselling for 24 weeks | ND |
| Unick2012[44] | SBWL+technology | As SBWL with activity tracker and self-monitoring | ND |
| Unick2012[44] | SBWL | Weekly group meetings focusing on behavioural approaches to PA and dietary change. Structured exercise and calorie goals. Self-monitoring with paper diaries, written feedback weekly. | F2F |
| Van Wier2009,2011,2012[45-47] | Phone group | Intervention materials, with fortnightly phone counselling | ND |
| Wyke2015[48] | Football Fans In Training | 12 weekly sessions of gender-sensitised, football-based curriculum. Discussion-based educational sessions, physical activity sessions. | ND |
| Wyke2015[48] | Waitlist | Waitlist | DL |
| MIC, motivational interviewing, and internet condition; PR, paper record; NIC, nutrition psychoeducation and internet condition; LM, standard lifestyle modification; SBWI, standard behaviour weight loss intervention; EWLI, technology enhanced weight loss intervention; WW, weight watchers; LMW, Livin’ My Weigh; PA, physical activity; NPC, Nutrition Psychoeducation and Internet Condition; LEARN, Lifestyle, Exercise, Attitudes, Relationships, Nutrition; PC, primary care; PC, Personal coaching intervention group; PMRP, partial meal replacement program; DVR, digital voice recorder; CPCC, Certified Professional Co-Active Coaches; SBWL, standard behavioural weight loss; CBT, cognitive behavioural therapy; SHG, self-help guidance; HBCSS, health behaviour change support system; I, internet; IPS, In person support; SRI, self-regulatory intervention; EX, aerobic exercise; BWL, behaviour weight loss; GWL, Group-based behavioural weight loss education group; SELF, Self-guided; TECH, technology supported; STND, standard; PCP, primary care provider; CP, Cell phone intervention group | | | |

Bibliography

1. Allen JK, Stephens J, Dennison Himmelfarb CR, Stewart KJ, Hauck S. Randomized controlled pilot study testing use of smartphone technology for obesity treatment. J Obes. 2013;2013:151597. PMID: 24392223. doi: 10.1155/2013/151597.

2. Almeida FA, You W, Harden SM, Blackman KC, Davy BM, Glasgow RE, et al. Effectiveness of a worksite-based weight loss randomized controlled trial: the worksite study. Obesity (Silver Spring). 2015 Apr;23(4):737-45. PMID: 25678325. doi: 10.1002/oby.20899.

3. Barnes RD, White MA, Martino S, Grilo CM. A randomized controlled trial comparing scalable weight loss treatments in primary care. Obesity (Silver Spring). 2014 Dec;22(12):2508-16. PMID: 25298016. doi: 10.1002/oby.20889.

4. Barnes RD, Ivezaj V, Martino S, Pittman BP, Grilo CM. Back to Basics? No Weight Loss from Motivational Interviewing Compared to Nutrition Psychoeducation at One-Year Follow-Up. Obesity (Silver Spring). 2017 Dec;25(12):2074-8. PMID: 29086484. doi: 10.1002/oby.21972.

5. Bergman F, Wahlstrom V, Stomby A, Otten J, Lanthen E, Renklint R, et al. Treadmill workstations in office workers who are overweight or obese: a randomised controlled trial. Lancet Public Health. 2018 Nov;3(11):e523-e35. PMID: 30322782. doi: 10.1016/S2468-2667(18)30163-4.

6. Brindal E, Hendrie GA, Taylor P, Freyne J, Noakes M. Cohort Analysis of a 24-Week Randomized Controlled Trial to Assess the Efficacy of a Novel, Partial Meal Replacement Program Targeting Weight Loss and Risk Factor Reduction in Overweight/Obese Adults. Nutrients. 2016 May 4;8(5). PMID: 27153085. doi: 10.3390/nu8050265.

7. Brindal E, Hendrie GA, Freyne J, Noakes M. Incorporating a Static Versus Supportive Mobile Phone App Into a Partial Meal Replacement Program With Face-to-Face Support: Randomized Controlled Trial. JMIR Mhealth Uhealth. 2018 Apr 18;6(4):e41. PMID: 29669704. doi: 10.2196/mhealth.7796.

8. Burke LE, Conroy MB, Sereika SM, Elci OU, Styn MA, Acharya SD, et al. The effect of electronic self-monitoring on weight loss and dietary intake: a randomized behavioral weight loss trial. Obesity (Silver Spring). 2011 Feb;19(2):338-44. PMID: 20847736. doi: 10.1038/oby.2010.208.

9. Burke LE, Styn MA, Sereika SM, Conroy MB, Ye L, Glanz K, et al. Using mHealth technology to enhance self-monitoring for weight loss: a randomized trial. Am J Prev Med. 2012 Jul;43(1):20-6. PMID: 22704741. doi: 10.1016/j.amepre.2012.03.016.

10. Turk MW, Elci OU, Wang J, Sereika SM, Ewing LJ, Acharya SD, et al. Self-monitoring as a mediator of weight loss in the SMART randomized clinical trial. Int J Behav Med. 2013 Dec;20(4):556-61. PMID: 22936524. doi: 10.1007/s12529-012-9259-9.

11. Conroy MB, Yang K, Elci OU, Gabriel KP, Styn MA, Wang J, et al. Physical activity self-monitoring and weight loss: 6-month results of the SMART trial. Med Sci Sports Exerc. 2011 Aug;43(8):1568-74. PMID: 21200337. doi: 10.1249/MSS.0b013e31820b9395.

12. Butryn ML, Martinelli MK, Crane NT, Godfrey K, Roberts SR, Zhang F, et al. Counselor Surveillance of Digital Self-Monitoring Data: A Pilot Randomized Controlled Trial. Obesity (Silver Spring). 2020 Dec;28(12):2339-46. PMID: 33098278. doi: 10.1002/oby.23015.

13. Gerber BS, Schiffer L, Brown AA, Berbaum ML, Rimmer JH, Braunschweig CL, et al. Video telehealth for weight maintenance of African-American women. J Telemed Telecare. 2013 Jul;19(5):266-72. PMID: 24163236. doi: 10.1177/1357633X13490901.

14. Godino JG, Merchant G, Norman GJ, Donohue MC, Marshall SJ, Fowler JH, et al. Using social and mobile tools for weight loss in overweight and obese young adults (Project SMART): a 2 year, parallel-group, randomised, controlled trial. Lancet Diabetes Endocrinol. 2016 Sep;4(9):747-55. PMID: 27426247. doi: 10.1016/S2213-8587(16)30105-X.

15. Griffith DM, Pennings JS, Jaeger EC. Mighty Men: A Pilot Test of the Feasibility and Acceptability of a Faith-Based, Individually Tailored, Cluster-Randomized Weight Loss Trial for Middle-Aged and Older African American Men. Am J Mens Health. 2023 Jul-Aug;17(4):15579883231193235. PMID: 37608590. doi: 10.1177/15579883231193235.

16. Hernandez-Reyes A, Camara-Martos F, Molina-Luque R, Moreno-Rojas R. Effect of an mHealth Intervention Using a Pedometer App With Full In-Person Counseling on Body Composition of Overweight Adults: Randomized Controlled Weight Loss Trial. JMIR Mhealth Uhealth. 2020 May 27;8(5):e16999. PMID: 32348263. doi: 10.2196/16999.

17. Hernandez-Reyes A, Camara-Martos F, Molina Recio G, Molina-Luque R, Romero-Saldana M, Moreno Rojas R. Push Notifications From a Mobile App to Improve the Body Composition of Overweight or Obese Women: Randomized Controlled Trial. JMIR Mhealth Uhealth. 2020 Feb 12;8(2):e13747. PMID: 32049065. doi: 10.2196/13747.

18. Jakicic JM, Davis KK, Rogers RJ, King WC, Marcus MD, Helsel D, et al. Effect of Wearable Technology Combined With a Lifestyle Intervention on Long-term Weight Loss: The IDEA Randomized Clinical Trial. JAMA. 2016 Sep 20;316(11):1161-71. PMID: 27654602. doi: 10.1001/jama.2016.12858.

19. Johnston CA, Rost S, Miller-Kovach K, Moreno JP, Foreyt JP. A randomized controlled trial of a community-based behavioral counseling program. Am J Med. 2013 Dec;126(12):1143 e19-24. PMID: 24135513. doi: 10.1016/j.amjmed.2013.04.025.

20. Karmali S. A Coaching and/or Education Intervention Targeting Physical Activity and Nutrition Behaviours in Parents with Overweight/Obesity and their Children [Thesis]. Ann Arbor: The University of Western Ontario; 2020.

21. Kim JY, Oh S, Steinhubl S, Kim S, Bae WK, Han JS, et al. Effectiveness of 6 months of tailored text message reminders for obese male participants in a worksite weight loss program: randomized controlled trial. JMIR Mhealth Uhealth. 2015 Feb 3;3(1):e14. PMID: 25648325. doi: 10.2196/mhealth.3949.

22. Kurtzman GW, Day SC, Small DS, Lynch M, Zhu J, Wang W, et al. Social Incentives and Gamification to Promote Weight Loss: The LOSE IT Randomized, Controlled Trial. J Gen Intern Med. 2018 Oct;33(10):1669-75. PMID: 30003481. doi: 10.1007/s11606-018-4552-1.

23. LaRose JG, Tate DF, Lanoye A, Fava JL, Jelalian E, Blumenthal M, et al. Adapting evidence-based behavioral weight loss programs for emerging adults: A pilot randomized controlled trial. J Health Psychol. 2019 Jun;24(7):870-87. PMID: 28810394. doi: 10.1177/1359105316688951.

24. Leahey TM, Subak LL, Fava J, Schembri M, Thomas G, Xu X, et al. Benefits of adding small financial incentives or optional group meetings to a web-based statewide obesity initiative. Obesity (Silver Spring). 2015 Jan;23(1):70-6. PMID: 25384463. doi: 10.1002/oby.20937.

25. Lin PH, Wang Y, Levine E, Askew S, Lin S, Chang C, et al. A text messaging-assisted randomized lifestyle weight loss clinical trial among overweight adults in Beijing. Obesity (Silver Spring). 2014 May;22(5):E29-37. PMID: 24375969. doi: 10.1002/oby.20686.

26. Little P, Stuart B, Hobbs FR, Kelly J, Smith ER, Bradbury KJ, et al. An internet-based intervention with brief nurse support to manage obesity in primary care (POWeR+): a pragmatic, parallel-group, randomised controlled trial. Lancet Diabetes Endocrinol. 2016 Oct;4(10):821-8. PMID: 27474214. doi: 10.1016/S2213-8587(16)30099-7.

27. Little P, Stuart B, Hobbs FR, Kelly J, Smith ER, Bradbury KJ, et al. Randomised controlled trial and economic analysis of an internet-based weight management programme: POWeR+ (Positive Online Weight Reduction). Health Technol Assess. 2017 Jan;21(4):1-62. PMID: 28122658. doi: 10.3310/hta21040.

28. Madrona Marcos F, Panisello Royo JM, Tarraga Marcos ML, Rosich N, Carbayo Herencia JA, Alins J, et al. Effect of a motivational physical activity program on lipid parameters in patients with obesity and overweight. Clin Investig Arterioscler. 2019 Nov-Dec;31(6):245-50. PMID: 30971374. doi: 10.1016/j.arteri.2019.02.002.

29. Melchart D, Low P, Wuhr E, Kehl V, Weidenhammer W. Effects of a tailored lifestyle self-management intervention (TALENT) study on weight reduction: a randomized controlled trial. Diabetes Metab Syndr Obes. 2017;10:235-45. PMID: 28684917. doi: 10.2147/DMSO.S135572.

30. Micco N, Gold B, Buzzell P, Leonard H, Pintauro S, Harvey-Berino J. Minimal in-person support as an adjunct to internet obesity treatment. Annals of behavioral medicine : a publication of the Society of Behavioral Medicine. 2007;33(1):49-56.

31. Nicklas BJ, Gaukstern JE, Beavers KM, Newman JC, Leng X, Rejeski WJ. Self-monitoring of spontaneous physical activity and sedentary behavior to prevent weight regain in older adults. Obesity (Silver Spring). 2014 Jun;22(6):1406-12. PMID: 24585701. doi: 10.1002/oby.20732.

32. Pellegrini CA, Verba SD, Otto AD, Helsel DL, Davis KK, Jakicic JM. The comparison of a technology-based system and an in-person behavioral weight loss intervention. Obesity (Silver Spring). 2012 Feb;20(2):356-63. PMID: 21311506. doi: 10.1038/oby.2011.13.

33. Pinto AM, Fava JL, Hoffmann DA, Wing RR. Combining behavioral weight loss treatment and a commercial program: a randomized clinical trial. Obesity (Silver Spring). 2013 Apr;21(4):673-80. PMID: 23404824. doi: 10.1002/oby.20044.

34. Shuger SL, Barry VW, Sui X, McClain A, Hand GA, Wilcox S, et al. Electronic feedback in a diet- and physical activity-based lifestyle intervention for weight loss: a randomized controlled trial. Int J Behav Nutr Phys Act. 2011 May 18;8:41. PMID: 21592351. doi: 10.1186/1479-5868-8-41.

35. Spring B, Pellegrini CA, Pfammatter A, Duncan JM, Pictor A, McFadden HG, et al. Effects of an abbreviated obesity intervention supported by mobile technology: The ENGAGED randomized clinical trial. Obesity (Silver Spring). 2017 Jul;25(7):1191-8. PMID: 28494136. doi: 10.1002/oby.21842.

36. Sullivan DK, Goetz JR, Gibson CA, Washburn RA, Smith BK, Lee J, et al. Improving weight maintenance using virtual reality (Second Life). J Nutr Educ Behav. 2013 May-Jun;45(3):264-8. PMID: 23622351. doi: 10.1016/j.jneb.2012.10.007.

37. Svetkey LP, Batch BC, Lin PH, Intille SS, Corsino L, Tyson CC, et al. Cell phone intervention for you (CITY): A randomized, controlled trial of behavioral weight loss intervention for young adults using mobile technology. Obesity (Silver Spring). 2015 Nov;23(11):2133-41. PMID: 26530929. doi: 10.1002/oby.21226.

38. Lin PH, Grambow S, Intille S, Gallis JA, Lazenka T, Bosworth H, et al. The Association Between Engagement and Weight Loss Through Personal Coaching and Cell Phone Interventions in Young Adults: Randomized Controlled Trial. JMIR Mhealth Uhealth. 2018 Oct 18;6(10):e10471. PMID: 30341051. doi: 10.2196/10471.

39. Tarraga Marcos ML, Panisello Royo JM, Carbayo Herencia JA, Rosich Domenech N, Alins Presas J, Tarraga Lopez PJ. Effect on the lipid parameters of an intervention to reduce weight in overweight and obese patients. Clin Investig Arterioscler. 2017 May-Jun;29(3):103-10. PMID: 28318619. doi: 10.1016/j.arteri.2017.01.002.

40. Tate DF, Lutes LD, Bryant M, Truesdale KP, Hatley KE, Griffiths Z, et al. Efficacy of a Commercial Weight Management Program Compared With a Do-It-Yourself Approach: A Randomized Clinical Trial. JAMA Netw Open. 2022 Aug 1;5(8):e2226561. PMID: 35972742. doi: 10.1001/jamanetworkopen.2022.26561.

41. Teeriniemi AM, Salonurmi T, Jokelainen T, Vahanikkila H, Alahaivala T, Karppinen P, et al. A randomized clinical trial of the effectiveness of a Web-based health behaviour change support system and group lifestyle counselling on body weight loss in overweight and obese subjects: 2-year outcomes. J Intern Med. 2018 Nov;284(5):534-45. PMID: 29974563. doi: 10.1111/joim.12802.

42. Thomas JG, Bond DS, Raynor HA, Papandonatos GD, Wing RR. Comparison of Smartphone-Based Behavioral Obesity Treatment With Gold Standard Group Treatment and Control: A Randomized Trial. Obesity (Silver Spring). 2019 Apr;27(4):572-80. PMID: 30779333. doi: 10.1002/oby.22410.

43. Thompson WG, Kuhle CL, Koepp GA, McCrady-Spitzer SK, Levine JA. "Go4Life" exercise counseling, accelerometer feedback, and activity levels in older people. Arch Gerontol Geriatr. 2014 May-Jun;58(3):314-9. PMID: 24485546. doi: 10.1016/j.archger.2014.01.004.

44. Unick JL, O'Leary KC, Bond DS, Wing RR. Physical activity enhancement to a behavioral weight loss program for severely obese individuals: A preliminary investigation. ISRN Obes. 2012 Sep 5;2012. PMID: 24379985. doi: 10.5402/2012/465158.

45. van Wier MF, Ariens GA, Dekkers JC, Hendriksen IJ, Smid T, van Mechelen W. Phone and e-mail counselling are effective for weight management in an overweight working population: a randomized controlled trial. BMC Public Health. 2009 Jan 9;9:6. PMID: 19134171. doi: 10.1186/1471-2458-9-6.

46. van Wier MF, Dekkers JC, Hendriksen IJ, Heymans MW, Ariens GA, Pronk NP, et al. Effectiveness of phone and e-mail lifestyle counseling for long term weight control among overweight employees. J Occup Environ Med. 2011 Jun;53(6):680-6. PMID: 21654441. doi: 10.1097/JOM.0b013e31821f2bbb.

47. van Wier MF, Dekkers JC, Bosmans JE, Heymans MW, Hendriksen IJ, Pronk NP, et al. Economic evaluation of a weight control program with e-mail and telephone counseling among overweight employees: a randomized controlled trial. Int J Behav Nutr Phys Act. 2012 Sep 11;9:112. PMID: 22967224. doi: 10.1186/1479-5868-9-112.

48. Wyke S, Hunt K, Gray CM, Fenwick E, Bunn C, Donnan PT, et al. Football Fans in Training (FFIT): a randomised controlled trial of a gender-sensitised weight loss and healthy living programme for men - end of study report. Public Health Research. 2015. doi: <https://dx.doi.org/10.3310/phr03020>.
