# Supplementary material for: Evaluation of the Aspects of Digital Interventions That Successfully Support Weight Loss: Systematic Review With Component Network Meta-Analysis
Source: J Med Internet Res. 2025 May 22;27:e65443. doi: 10.2196/65443 (PMC12141966; doi:10.2196/65443)
Supplement: Multimedia Appendix 5 [file jmir_v27i1e65443_app5.docx]

**Multimedia Appendix 5.** Intervention coding table.

| Study | Trial arm (**I**ntervention/**C**ontrol) | Trial arm label | Trial arm category | Intervention targets (**D**iet/**E**xercise/**B**oth) | Overview of arm | Duration | Face to face content (**G**roup/**I**ndividual/**B**oth/n/a) | Tailored/ personalised (Yes/No) | Goal setting | Information | Feedback | Peer support | Reminders | Competition/ challenge | Specialist contact | Self-monitoring | Incentives/ rewards |
| --- | --- | --- | --- | --- | --- | --- | --- | --- | --- | --- | --- | --- | --- | --- | --- | --- | --- |
| Apiñaniz 2019[1] | I | Intervention | DL | B | Min 150 mins moderate or 75 mins vigorous exercise or a mix/wk; muscle strengthening at least 2x/wk; text messages 1/day for 1st month, 2/wk 2nd-6th month | 6m | n/a | No |  | APP, SMS, VID |  |  | SMS |  |  |  |  |
| Apiñaniz 2019[1] | C | Control | Tau+ | B | Health information only | 6m | n/a | No |  |  |  |  |  |  |  |  |  |
| Backman 2022[2] | C | Control | DO | B | Directed to website | 17w | n/a | No |  | WEB |  |  |  |  |  |  |  |
| Backman 2022[2] | I | Koa Family Programme | DL | B | Whole Health Programme (WHP): 17 weekly, 90 min, group interactive Zoom sessions with health coach; up to three, 15-minute personal check-ins with health coach, private Facebook group; weekly texts | 17w | G | No |  | VC, FBG | VC | FBG, SMS | SMS |  | VC |  |  |
| Beleigoli 2020[3] | I | Platform only | DO | B | 24-week behaviour change program delivered using a web platform with personalized computer-delivered feedback | 24w | n/a | Yes | WBP | WBP | WBP | WBP | EML | WBP |  | WBP |  |
| Beleigoli 2020[3] | I | Platform + coaching | DO | B | Same 24-week web-based behaviour change program plus 12 weeks of personalized feedback delivered online by a dietitian | 24w | n/a | Yes | WBP | WBP | WBP | WBP | EML | WBP | WBP | WBP |  |
| Beleigoli 2020[3] | C | Waitlist | WL | B | Dietary and PA recommendations through e-booklet; 4x 5-minute educational videos | 24m | n/a | No |  | e-book, VID |  |  | EML |  |  | EML, WBP |  |
| Bennett 2013[4], Lanpher 2016 | C | Usual care | TAU | B | Wellness newsletters every six months | 12m | n/a | No |  |  |  |  |  |  |  |  |  |
| Bennett 2013[4], Lanpher 2016[5] | I | Shape Plan | DL | B | Baseline goals revised every 6 weeks. 52x weekly IVR calls, 12x monthly 1-on-1 calls with dietitian | 12m | I | Yes | iOTA | IVR | IVR |  |  |  |  | IVR |  |
| Berli 2021[6] | I | Intervention | DL | E | Partner provides peer support. one action control text message each weekday | 14d | I | No | SMS |  |  |  | SMS |  |  | SMS, APP |  |
| Berli 2021[6] | C | Control | DO | E | Text message to remind to complete diary | 14d | n/a | No |  |  |  |  | SMS |  |  | APP |  |
| Braun 2022[7] | I | Mindful Self Compassion | DO | B | 4 months virtually delivered lifestyle modification programme, followed by 8-week group mindful self-compassion intervention – weekly 2 hr session | 6m | n/a | Yes | EML, VC | VC | EML, VC | VC |  |  | VC | EML (assumed) |  |
| Braun 2022[7] | C | Con | DO | B | 4 months virtually delivered lifestyle modification programme, followed by 8 week of group nutrition and cooking class - weekly 1.5 hr session | 6m | n/a | Yes | EML, VC | VC | EML, VC | VC |  |  | VC | EML (assumed) |  |
| Burke 2022[8] | I | Self-monitoring and feedback | DO | B | 90-minute 1:1 session with a dietitian, wireless scale and Fitbit Charge 2. Daily self-weighing, aim for weight loss of 1 to 2 lb per week or 7-10% overall. Up to 3 feedback messages/day | 12m | n/a | Yes |  |  | APP |  | AT, EML |  |  | AT, APP |  |
| Burke 2022[8] | C | Self-monitoring | DO | B | 90-minute 1:1 session with a dietitian, wireless scale and Fitbit Charge 2. Daily self-weighing, aim for weight loss of 1 to 2 lb per week or 7-10% overall. | 12m | n/a | No |  |  |  |  | AT, EML |  |  | AT, APP |  |
| Burke 2011,2012 [9, 10]; Turk 2013[11]; Conroy 2011[12] | C | PDA | DL | B | Daily self-monitoring of diet and PA; daily/weekly PA goals | 24m | G | No | PDA | PDA |  |  |  |  |  | PDA |  |
| Burke 2011,2012 [9, 10]; Turk 2013[11]; Conroy 2011[12] | I | PDA + Feedback | DL | B | Software on PDAs providing daily feedback on entries, positive reinforcement, and guidance for goal attainment. Daily self-monitoring of diet and PA; daily/weekly PA goals | 24m | G | Yes | PDA | PDA | PDA |  | PDA |  |  | PDA |  |
| Carter 2013[13] | I | Smartphone | DO | B | My Meal Mate app; goal setting, self-monitoring of diet and PA, feedback via weekly text messages | 6m | n/a | Yes | APP | APP | APP, SMS |  |  |  |  | APP |  |
| Carter 2013[13] | C | WEB | DO | B | Self-monitoring slimming website | 6m | n/a | No |  |  |  |  |  |  |  | WEB |  |
| Carter 2013[13] | C | Diary | TAU+ | B | Paper food diary and calorie counting book | 6m | n/a | No |  |  |  |  |  |  |  |  |  |
| Collins 2013[14] | I | Enhanced | DO | B | As ‘Basic’ plus weekly feedback and reminders. Option to repeat after 12 weeks or take seasonal variation. | 24w | n/a | Yes | WBP | EML, NEW | DIA, WBP | DIS | EML, SMS |  |  | WBP, SMS, DIA |  |
| Collins 2013[14] | C | Basic | DO | B | Weekly email to self-monitor, online diary, daily feedback, weekly information, individualised daily targets. | 24w | n/a | Yes | WBP | EML, NEW | DIA | DIS |  |  |  | WEB, SMS, DIA |  |
| Collins 2017[15] | I | Enhanced | DO | B | As ‘Basic’ plus weekly feedback and reminders. Option to repeat after 12 weeks or take seasonal variation. Weight maintenance. | 24w | n/a | Yes | WBP | EML, NEW | DIA, WBP | DIS | EML, SMS |  |  | WEB, SMS, DIA |  |
| Collins 2017[15] | C | Basic | DO | B | Weekly email to self-monitor, online diary, feedback, weekly online information, individualised daily targets. Weight maintenance. | 12m | n/a | Yes | WBP | EML, NEW | DIA | DIS |  |  |  | WEB, SMS, DIA |  |
| Conroy 2019[16] | I | Coaching | DO | B | Personalised weekly health coaching through the Electronic Health Record patient portal | 24m | n/a | Yes |  | WBP | WBP |  | EML, EHR |  | WBP | EHR |  |
| Conroy 2019[16] | C | Tracking | DO | B | Electronic Health Record tracking tools, daily log in tracking weight, diet and PA | 24m | n/a | No |  |  |  |  | EHR |  |  | EHR |  |
| Dombrowski 2020[17, 18] | C | Waitlist | WL | n/a | Waitlist (provided pedometer and WEB access at start) | 12m | n/a | n/a |  | WEB |  |  |  |  |  | PED |  |
| Dombrowski 2020[17, 18] | I | SMS | DO | NR | Narrative SMS: 0-5 texts/day for 12 months | 12m | n/a | No | SMS | WEB, SMS | SMS | SMS | SMS | SMS |  | WEB, PED |  |
| Dombrowski 2020[17, 18] | I | SMS + Incentive | DO | NR | Narrative SMS: 0-5 texts/day for 12 months, cash incentive | 12m | n/a | Yes | SMS | WEB, SMS | SMS | SMS | SMS | SMS |  | WEB, PED | SMS, WEB |
| Duncan 2020[19] | I | Traditional/enhanced | DL | B | One in person counselling session with dietician. Weekly feedback emails, weekly prompts , daily self-monitoring and app use for diet and physical activity (and sleep for enhanced group) | 6m | I | Yes | APP, SMS | APP, SMS | APP, EML, |  | SMS, EML |  |  | APP, AT, WEB |  |
| Duncan 2020[19] | C | Waitlist | WL | B | n/a | 6m | n/a | n/a |  |  |  |  |  |  |  |  |  |
| Dunn 2019[20] | I | Photo group | DO | D | Twice-weekly behavioural weight-loss podcasts and tracked diet using a photo app | 6m | n/a | No |  | POD | APP | APP | EML |  | EML | APP |  |
| Dunn 2019[20] | C | Calorie group | DO | D | Twice-weekly behavioural weight-loss podcasts and tracked diet using a calorie-tracking app | 6m | n/a | No | APP | POD, APP |  |  | EML |  | EML | APP |  |
| Eisenhauer 2021[21] | I | MT+ | DO | B | Premium app content. One/twice-daily reminder messages, daily information messages, weekly self-monitoring challenges and discussion board content. Daily self-monitoring with feedback. | 6m | n/a | Yes | APP | SMS | APP | APP, DIS | SMS | DIS | SMS | APP |  |
| Eisenhauer 2021[21] | C | MT | DO | B | Basic app. Daily self-monitoring only | 6m | n/a | No |  |  |  |  |  |  |  | APP |  |
| Falkenhain 2021[22] | C | Calorie restricted low fat diet app | DO | D | Participants record calorie intake in the app. Daily points value calculated and instructed not to exceed | 12w | n/a | No | APP | APP | APP | APP |  |  |  | APP |  |
| Falkenhain 2021[22] | I | Ketogenic diet app with biofeedback | DO | D | Biofeedback device used 3x/day, with their level of ketosis provided within the paired app, feedback given | 12w | n/a | No |  | APP | APP, biofeedback | APP |  |  |  | APP, biofeedback |  |
| Gemesi 2024[23] | I | ADHOC | DO | B | Use app for 24 weeks | 24w | n/a | No | APP | APP | APP |  |  |  |  | APP |  |
| Gemesi 2024[23] | C | EXPECT | DO | B | Use app for 12 weeks, starting after 12-week delay | 12w | n/a | No | APP | APP | APP |  |  |  |  | APP |  |
| Gold 2007[24] | C | E.Diets | DO | B | Access to commercial website for duration of study without guidance. Automated feedback | 12m | n/a | Yes | WBP | WBP | WBP | WBP |  |  |  | WBP |  |
| Gold 2007[24] | I | Vtrim | DO | B | Weekly online therapist-led meetings for 6 months, then bi-weekly for 6 months (maintenance phase) | 12m | n/a | Yes | WBP | WBP | WBP, EML | WBP |  |  | WBP, EML | WBP |  |
| Haapala 2009[25] | I | Experimental Group | DO | B | Staggered reduction of food intake and daily weight reporting with immediate tailored feedback | 12m | n/a | Yes | SMS | WEB | SMS |  | SMS |  |  | WEB, SMS |  |
| Haapala 2009[25] | C | Control Group | WL | B | n/a | 12m | n/a | n/a |  |  |  |  |  |  |  |  |  |
| Hageman 2017[26] | I | WE | DO | B | Web-based behaviour change lifestyle program plus professional email counselling, weekly feedback on posts 1-6 months, bi-weekly 6-18 months | 30m | n/a | Yes | WEB | WEB | EML, WEB |  | EML |  | EML | WEB, PED |  |
| Hageman 2017[26] | I | WD | DO | B | Web-based behaviour change lifestyle program plus supplemental peer-led discussion blog | 30m | n/a | n | WEB | WEB | WEB | DIS |  |  |  | WEB, PED |  |
| Hageman 2017[26] | C | WO | DO | B | Web-based behaviour change lifestyle programme, with diet and PA recommendations, goal setting, self-monitoring daily then weekly | 30m | n/a | No | WEB | WEB | WEB |  |  |  |  | WEB, PED |  |
| Hesseldal 2022[27], Christensen 2022[28] | I | Digital coaching | DL | B | One or two meetings (face-to-face or digital) with health coach, then asynchronous web-based consultations via SMS or video, 1/wk first 6 months, then 1/month | 12m | I | Yes | VC (APP/WEB) | VC (APP/WEB) | VC(APP/WEB) | DIS (APP/WEB) | APP/WEB |  | SMS,WEB, APP, VID | APP, WEB |  |
| Hesseldal 2022[27], Christensen 2022[28] | C | Usual care | TAU | B | Usual care - NR | 12m | n/a | No |  |  |  |  |  |  |  |  |  |
| Hutchesson 2018[29] | C | Control | WL | n/a | n/a | 6m | n/a | n/a |  |  |  |  |  |  |  |  |  |
| Hutchesson 2018[29] | I | BPBH | DO | B | Website with advice, information, quizzes, and feedback. Weekly to bi-weekly email newsletters and twice weekly to weekly text messages. Three social media posts/wk (education, challenge, and practical tip) Weekly self-monitoring | 6m | n/a | Yes | WEB | WEB, EML, SOC | WEB, APP, EML | SOC | EML, SMS, NEW | WEB, EML, NEW, SOC | WEB, EML | WEB, APP | EML, SMS, NEW |
| Jane 2017[30]2018[31] | I | FG | DO | B | Condensed version of the diet available through Facebook group | 24w | n/a | No |  | FBG |  | FBG |  |  |  | PED |  |
| Jane 2017[30], 2018[31] | I | PG | DO | B | Condensed version of the diet as printed info and pedometer with target steps | 24w | n/a | No |  |  |  |  |  |  |  | PED |  |
| Jane 2017[30], 2018[31] | C | CG | TAU | B | Instructed to follow the Australian Government dietary and physical activity guidelines | 24w | n/a | No |  |  |  |  |  |  |  |  |  |
| Joseph 2023[32] | I | Smart-walk | DO | E | Culturally tailored video and text-based PA promotion modules, online discussion, and a PA self-monitoring feature that integrated with Fitbit activity monitors, 3 PA texts each week | 8m | n/a | Yes | APP | APP, SMS |  | APP | SMS |  |  | APP, AT |  |
| Joseph 2023[32] | C | Smart-health | DO | n/a | Smartphone-delivered culturally tailored health promotion intervention, focusing on health topics not related to PA and cardiometabolic disease risk. No PA tracking tool | 8m | n/a | Yes |  | APP, SMS |  | APP | SMS |  |  |  |  |
| Kempf 2019[33] | I | TMC | DL | B | Weekly care calls in months 3–6 then monthly calls from months 7 to 12. Self-monitor for whole 12 months. | 12m | n/a | Yes |  |  |  |  |  |  |  | PED, WBP |  |
| Kempf 2019[33] | C | C1 | DO | B | Self-monitor for 12 months, no other input | 12m | n/a | No |  |  |  |  |  |  |  | PED, WBP |  |
| Kempf 2019[33] | I | C2 | DL | B | Self-monitor months 6-12. Weekly tele-coaching calls from months 6-9 | 12m | n/a | Yes |  |  |  |  |  |  |  | PED, WBP |  |
| Kempf 2018[34] | I | TM | DO | B | Monitoring of weight and steps (daily/weekly) | 12w | n/a | No |  |  |  |  |  |  |  | PED, WBP |  |
| Kempf 2018[34] | C | Control | TAU | B | Usual care | 12w | n/w | No |  |  |  |  |  |  |  |  |  |
| Kempf 2018[34] | I | TMC | DL | B | Monitoring of weight and steps (daily/weekly) with weekly care calls with trained coaches | 12w | I | Yes |  |  |  |  |  |  |  | PED, WBP |  |
| Keshavarz 2023[35] | I | Online circuit training | DO | E | Exercise 3x/wk for 12 wk with 24/36 sessions supervised via Microsoft Teams. Supervision tapered out. | 12w | n/a | No |  | VC |  | VC |  |  |  |  |  |
| Keshavarz 2023[35] | C | Online workout plan | DO | E | Received workout plan | 12w | n/a | No |  | WEB |  |  |  |  |  |  |  |
| Kharmats 2022[36] | I | SMS group | DO | B | Min 7 text messages each week for 16 weeks | 16w | n/a | Yes | SMS | SMS | SMS |  | SMS |  |  |  |  |
| Kharmats 2022[36] | C | Printed messages group | TAU+ | B | 8 weeks of messages given at baseline, then again after 8 weeks. | 16w | n/a | No |  |  |  |  |  |  |  |  |  |
| Kim 2020[37] | I | Digital CBT | DO | B | Daily coaching program with daily logging of food, weight and PA; group missions; personalised report and feedback; daily messages | 24w | n/a | Yes | APP |  | APP | APP |  | APP | APP | SMS, APP |  |
| Kim 2020[37] | C | Self-reported | DO | D | Food diary app without therapist input | 24w | n/a | No |  |  |  |  |  |  |  | APP |  |
| Kohl 2023[38] | I | Interactive web-based program | DO | B | Interactive web-based weight loss program, weight and PA tracking, weekly tasks | 12w | n/a | Yes | APP | APP | APP |  |  |  |  | APP |  |
| Kohl 2023[38] | C | Non-interactive web-based program | DO | B | Non-interactive web-based weight loss information | 12w | n/a | No |  | WBP |  |  |  |  |  |  |  |
| Kurtzman 2018[39] | C | Control | DO | B | Goals of 10,000 steps/day and 6-8% weight loss | 36w | n/a | No | APP |  |  | APP |  |  |  | APP, PED |  |
| Kurtzman 2018[39] | I | Gamification | DO | B | Weekly gamification with daily contact for 36 weeks and enhanced collaborative social incentives | 36w | n/a | No | APP, SMS or EML |  |  | APP | SMS, EML | APP |  | APP, PED, VC | APP |
| Laing 2014[40] | C | Usual care | TAU | B | NR | 6m | n/a | No |  |  |  |  |  |  |  |  |  |
| Laing 2014[40] | I | Usual care plus smartphone app | DL | B | Use app at will | 6m | n/a | Yes | APP | APP | APP | APP | APP | APP |  | APP |  |
| LaRose 2022[41] | I | aBWL + BE | DL | B | One group and one individual face to face session, and digital platform. Self-monitoring, goals, weekly feedback, weekly educational content, weekly messages, access to an optional private Facebook group to facilitate social support. Financial incentives for self-monitoring | 6m | B | Yes | EML | VID | EML, SMS | FBG |  |  |  | APP, PED |  |
| LaRose 2022[41] | C | aBWL | DL | B | Same as aBWL+BE, but without financial incentives | 6m | B | Yes | EML | VID | EML, SMS | FBG |  |  |  | APP, PED |  |
| LaRose 2022[41] | I | aBWL + SDT | DL | B | One group, one individual face to face session, and digital platform. Self-monitoring; goals; weekly feedback, educational content, messages; access to optional private Facebook group. 24 optional experiential group classes (eg, circuit training, cooking) | 6m | B | Yes | EML | VID | EML, SMS | FBG |  |  |  | APP, PED |  |
| Leahey 2015[42] | C | SI | DO | B | Weekly videos, self-monitoring platform, automated weekly feedback, automated weekly reminders | 3m | n/a | No |  | VID, WEB | WBP |  | WEB |  |  | WED, PED |  |
| Leahey 2015[42] | I | SII | DO | B | Weekly videos, self-monitoring platform, automated weekly feedback, automated weekly reminders plus messages linked with financial incentives | 3m | n/a | No |  | VID, WEB | WEB |  | WEB |  |  | WEB, PED | WEB |
| Leahey 2016[43] | C | Standard | DO | B | one-time group sessions plus periodic email contact | 10m | n/a | No |  | EML |  |  |  |  |  |  |  |
| Leahey 2016[43] | I | CB Pro | DO | B | Group session, self-monitoring and emails from professional | 10m | n/a | No | EML |  | EML |  | EML | EML | EML | EML, PED | EML |
| Leahey 2016[43] | I | CB peer | DO | B | Group session, self-monitoring and peer support | 10m | n/a | No | EML |  | EML | EML | EML | EML |  | EML, PED | EML |
| Little 2016[44, 45] | I | POWER+Remote | DL | D | 24-web based sessions and emails, 3x phone/email nurse contacts (+ 2 optional) | 6m | n/a | No |  | WEB |  |  | EML |  | EML |  |  |
| Little 2016[44, 45] | C | Control | TAU+ | D | Printable web-based pages | 6m | n/a | No |  |  |  |  |  |  |  |  |  |
| Markkanen 2024[46] | I | mHBCSS | DO | B | Information and persuasive software (e.g., reflective tasks and self-monitoring), twice a week. Then access to mHBCSS for 6 months, with a 3-week refresh period | 6m | n/a | No | APP | APP | APP |  | APP |  |  | APP |  |
| Markkanen 2024[46] | C | Waitlist | WL | n/a | n/a | 6m | n/a | n/a |  |  |  |  |  |  |  |  |  |
| McConnon 2007[47] | I | Internet group | DO | B | Instructed to log onto website at least weekly | 12m | n/a | Yes | WEB | WEB | WEB |  | EML |  |  | WEB |  |
| McConnon 2007[47] | C | Usual care | TAU | B | Standard written info only | 12m | n/a | No |  |  |  |  |  |  |  |  |  |
| Morgan 2009[48], 2011[49] | I | Internet | DO | B | 3-month online support | 3m | n/a | Yes | WEB, EMLs | WEB | EMLs | WEB |  |  |  | WEB |  |
| Morgan 2009[48, 49], 2011 | C | Information and self-help | TAU+ | B | Programme booklet | 3m | n/a | No |  |  |  |  |  |  |  |  |  |
| Morgan 2013[50], Blomfield 2014[51] | I | Resource group | DO | B | Self-monitor weight and waist circumference 1/wk,steps for 4 days 1/wk, set 3 SMART goals/month (1 for diet/PA/weight), identify sources of social support and strategies once/month | 3m | n/a | Yes |  | DVD |  |  |  |  |  | PED |  |
| Morgan 2013[50], Blomfield 2014[51] | I | Online group | DO | B | Use online food and exercise diary for 4 days each week. Participants asked to record weight on the website at least once/week | 3m | n/a | Yes |  | DVD | EMLs |  |  |  |  | WEB, PED |  |
| Morgan 2013[50], Blomfield 2014[51] | C | Waitlist | WL | B | None | 3m | n/a | n/a |  |  |  |  |  |  |  |  |  |
| Mueller 2022[52, 53], 2023 | I | SWiM-C | DL | B | Access on online web-platform with 12 weekly SWiM sessions | 4m | n/a | Yes |  | WBP |  |  | EML |  | EML | WEB |  |
| Mueller[52, 53], 2022, 2023 | C | Standard care | WL | B | Standard advice | 4m | n/a | No |  |  |  |  |  |  |  |  |  |
| Olson 2016[54], Wipfli 2019[55] | I | SHIFT | DL | B | Computer based-training, weekly self-monitoring. Up to 4 motivational coaching phone calls | 6m | I | Yes | WEB | WEB | WEB | WEB |  | WEB |  | WEB, PED |  |
| Olson 2016[54], Wipfli 2019[55] | C | Control | WL | n/a | Offered intervention after 6 months | 6m | n/a | n/a |  |  |  |  |  |  |  |  |  |
| Patel 2019[56] | I | Simultaneous | DO | B | Participants simultaneously tracked body weight and dietary intake each day and received additional behaviour change techniques via email | 3m | n/a | Yes | APP | EML | APP, EML |  | APP, EML |  |  | APP |  |
| Patel 2019[56] | I | Sequential | DO | B | Sequential arm, consisting of identical intervention components but allowing for mastery of one skill (i.e. self-monitoring of body weight) before beginning self-monitoring of diet | 3m | n/a | Yes | APP | EML | APP, EML |  | APP, EML |  |  | APP |  |
| Patel 2019[56] | C | App only | DO | B | App-Only arm that tracked diet with no behaviour change components | 3m | n/a | No | APP |  | APP |  | APP |  |  | APP |  |
| Patrick 2011[57] | I | Web-based intervention | DO | B | Initial assessment to tailor goals for behavioural targets, weekly learning activities, feedback | 12m | n/a | Yes | WEB | WEB | WEB |  |  |  | EML | WEB, PED |  |
| Patrick 2011[57] | C | Control | WL | n/a | Given access to alternative website containing general health information | 12m | n/a | n/a |  | WEB |  |  |  |  |  |  |  |
| Rogers 2016[58] | I | TECH | DL | B | Baseline orientation, monthly phone call, ad-lib use of BodyMedia® FIT system with activity monitor | 6m | I | n |  |  |  |  |  |  |  | WBP, AT |  |
| Rogers 2016[58] | I | EN-TECH | DL | B | Baseline orientation, monthly phone call, ad-lib use of BodyMedia® FIT system with activity monitor. Enhanced system for self-monitoring and feedback | 6m | I | Yes |  |  | WBP |  |  |  |  | WBP, APP, AT |  |
| Ross 2016[59] | I | TECH | DO | B | Baseline orientation, instructed to use daily, then no further contact | 6m | n/a | No |  |  | AT |  |  |  |  | AT |  |
| Ross 2016[59] | C | ST | DL | B | Baseline orientation, then no further contact | 6m | n/a | No |  |  |  |  |  |  |  | PED |  |
| Roth 2023[60] | I | Zanadio | DO | B | Access to *zanadio* app for 12 months | 12m | n/a | No | APP | APP, SMS, VID, WEB | APP | APP | APP |  | APP | APP |  |
| Roth 2023[60] | C | Waitlist | WL | n/a | n/a | n/a | n/a | n/a |  |  |  |  |  |  |  |  |  |
| Shapiro 2012[61] | C | Control | TAU+ | B | Monthly e-newsletter | 12m | n/a | No |  | NEW |  |  |  |  |  |  |  |
| Shapiro 2012[61] | I | Text4Diet | DO | B | 4 messages/day, monthly e-newsletter, access to WEB | 12m | n/a | Yes | SMS/MMS | SMS/MMS, WEB, NEW | SMS/MMS, WEB |  | SMS/MMS |  |  | PED, WEB |  |
| Shuger 2011[62] | I | SenseWear Alone | DO | B | Wear armband 16h, 7 days/wk | 9m | n/a | No |  |  | WEB |  |  |  |  | WEB, AT |  |
| Shuger 2011[62] | C | Standard care | TAU+ | B | Baseline self-directed weight loss manual | 9m | n/a | No |  |  |  |  |  |  |  |  |  |
| Silina 2017[63] | I | Intervention | DO | B | One SMS every 2 weeks | 12m | n/a | No |  | SMS |  |  |  |  |  |  |  |
| Silina 2017[63] | C | Control group | TAU | B | Baseline information | 12m | n/a | No |  |  |  |  |  |  |  |  |  |
| Simpson 2020[64, 65] | I | HelpMeDoIt! | DO | B | Use app/website at will for participant and helper. | 12m | n/a | Yes | APP | WEB | APP, EML | APP | APP | APP |  | APP | APP |
| Simpson 2020[64, 65] | C | Control group | TAU | B | Provided standard leaflets | 12m | n/a | No |  |  |  |  |  |  |  |  |  |
| Sniehotta 2019[66] | I | NuLevel | DL | B | Initial consultation, then free use of interface, weekly prompts to record weight | 12m | I | Yes | WBP | SMS | SMS |  | SMS |  |  | PED, WBP |  |
| Sniehotta 2019[66] | C | Control | TAU+ | B | 4 standard lifestyle coaching sessions, 3 months apart, delivered in SMS | 12m | n/a | No |  | SMS |  |  |  |  |  |  |  |
| Steinberg 2013[67] | I | Shape Plan | DL | B | Baseline orientation, daily SMS, weekly emails, daily self-monitoring, goals revised after 3 months | 6m | G | Yes | SMS, iOTA | VID | SMS, EML |  |  |  |  | SMS, PED |  |
| Steinberg 2013[67] | C | Education control | DL | B | Group education at baseline and 6 months; videos at 3 months; pedometers, 10,000 steps 'prescription' | 6m | G | No |  | VID |  |  |  |  |  | PED |  |
| Tate 2001[68] | C | Education | DL | B | Initial session then WEB access | 6m | G | No |  | WEB |  |  |  |  |  |  |  |
| Tate 2001[68] | I | Behaviour therapy | DL | B | Initial session then 24 weekly behavioural education content 'sessions' | 6m | G | Yes |  | WEB, EML | EML | DIS | EML |  | EML | DIA |  |
| Tate 2006[69] | C | NC | DO | B | *Slimfast* website available to public: weekly reporting and graphs of weight, weekly e-mail prompts to report weight, weekly weight loss tips via e-mail, recipes, and a weight loss e-buddy network system via email. Meal replacements for first week and coupons for remainder of intervention | 6m | n/a | No |  | WEB, EML |  | WEB,EML | EML |  |  | WEB |  |
| Tate 2006[69] | I | AF | DO | B | As control plus access to separate website with additional features, electronic diary, intervention specific message board, second weekly email to complete diary and weekly online lesson. Weekly feedback | 6m | n/a | Yes |  | WEB, EML | WEB | WEB,EML, DIS | EML |  |  | WEB, DIA |  |
| Tate 2006[69] | I | HC | DO | B | As AF but weekly feedback from human weight loss counsellor | 6m | n/a | Yes |  | WEB, EML | WEB, EML | WEB,EML DIS | EML |  | EML | WEB, DIA |  |
| Tate 2022[70] | I | IWL | DO | B | Use website at least 2 days/wk, self-monitor, tailored monthly feedback, 4x automated SMS/wk | 12m | n/a | Yes | WEB | WEB, SMS | WEB, SMS, EML | DIS |  |  |  | WEB, DIA | WEB |
| Tate 2022[70] | I | IWL+PCP | DO | B | Use website at least 2 days/wk, self-monitor, tailored monthly feedback, 4x automated SMS/wk, biweekly mails from PCP | 12m | n/a | Yes | WEB | WEB, SMS | WEB, SMS, EML | DIS |  |  | EML | WEB, DIA | WEB |
| Tate 2022[70] | C | EUC | TAU+ | B | Healthy weight booklet and goal setting worksheet | 12m | n/a | No |  |  |  |  |  |  |  |  |  |
| Teeriniemi 2018[71] | I | HBCSS | DO | B | Website providing weekly information, exercises, self-monitoring section, diary, weekly reminders | 12m | n/a | No | WEB | WEB | WEB |  |  |  |  | WEB |  |
| Teeriniemi 2018[71] | I | SHG + HBCSS | DL | B | 2x90 minute face-to-face group sessions plus website providing weekly information, exercises, self-monitoring section, diary, weekly reminders | 12m | G | No | WEB | WEB | WEB |  |  |  |  | WEB |  |
| Teeriniemi 2018[71] | C | Usual Care | TAU | B | Standard written info only | 12m | n/a | No |  |  |  |  |  |  |  |  |  |
| Thomas 2020[72] | C | WW | DO | B (D focus) | Access to website and app for 6 months | 6m | n/a | Yes | APP, WEB | APP, WEB |  |  |  |  |  | APP, WEB |  |
| Thomas 2020[72] | I | WW+ES | DO | B | Access to WW with Experience Success scenarios at weeks 2, 4, 6 and 8 | 6m | n/a | Yes | APP, WEB, WBP | APP, WEB, WBP | WBP | WBP |  | WBP |  | APP, WEB, WBP | WBP |
| Thomas 2017[73] | C | Control | TAU+ | B | Online newsletters | 12m | n/a | No |  | NEW |  |  |  |  |  |  |  |
| Thomas 2017[73] | I | WWO | DO | B | Use website daily and weekly | 12m | n/a | No | WEB | WEB |  |  |  |  |  | WEB, APP |  |
| Thomas 2017[73] | I | WWO+AL | DO | B | Encouraged to use daily and weekly. ActiveLink activity tracker used to set goals, provide feedback, set challenges and self-monitor. | 12m | n/a | No | WEB, APP | WEB | APP, AT |  |  | APP |  | WEB, APP, AT |  |
| Turner-McGrievy 2011[74] | I | Podcast + mobile | DO | B | 2 podcasts/wk for 3 months (approx. 15 mins each); 2 mini podcasts/wk for months 3–6 (approx. 5 mins each). Twitter used to create participant cohorts. | 6m | n/a | No | APP, POD | APP, POD |  | APP, SOC | SOC |  |  | APP |  |
| Turner-McGrievy 2011[74] | C | Podcast only | DO | B | 2 podcasts/wk for 3 months (approx. 15 mins each); 2 mini podcasts/wk for months 3–6 (approx. 5 mins each). | 6m | n/a | No | POD | POD |  |  |  |  |  |  |  |
| Turner-McGrievy 2017[75] | C | App | DO | B | 48 podcasts, use of app and pedometer | 6m | n/a | No |  | POD |  |  |  |  |  | APP, PED |  |
| Turner-McGrievy 2017[75] | I | Bite | DO | B | 48 podcasts, use of app, pedometer and bite counter | 6m | n/a | No |  | POD |  |  |  |  |  | BC, APP, PED |  |
| van Genugten 2012[76] | I | Tailored intervention | DO | B | 4 weekly modules, asked to visit website 3 or 4 times in 2 months | 6m | n/a | Yes | WEB | WEB | WEB | DIS | WEB |  |  |  |  |
| van Genugten 2012[76] | C | Generic intervention | DO | B | Access to website with 3 modules of information | 6m | n/a | No | WEB | WEB |  |  | EML |  |  |  |  |
| Van Wier 2009[77], 2011[78, 79] | I | Internet group | DO | B | Module-based web site, used ad-hoc with weekly reminders | 6m | n/a | No |  | WEB | EML |  | SMS, EML |  | EML |  |  |
| Van Wier 2009[77], 2011[78, 79] | C | Control group | TAU+ | B | Self-help materials at baseline | 6m | n/a | No |  |  |  |  |  |  |  |  |  |
| West 2016[80] | C | BT | DO | B | 1hr online chats weekly for 6 months then monthly for 12 months | 18m | n/a | Yes | WEB | WEB | EML | DIS, WEB |  |  | WEB (chat) | WEB, PED |  |
| West 2016[80] | I | BT + MI | DO | B | 1hr online chats weekly for 6 months then monthly for 12 months. 6 additional 30 min MI sessions | 18m | n/a | Yes | WEB | WEB | EML | DIS, WEB |  |  | WEB (chat), SMS | WEB, PED |  |
| West 2019[81] | I | Video-based chat with cellular-enabled scale | DO | B | Online behavioural treatment, 24 sessions, video chats with scales that automatically upload | 6m | n/a | Yes | VC | VC, WEB | VC, EML | VC, WEB |  |  | VC, EML | VC, WEB, APP,  AT |  |
| West 2019[81] | C | Text-based chat | DO | B | Online behavioural treatment, 24 sessions, text-based chats | 6m | n/a | Yes | SMS chat | SMS, WEB | SMS, EML | SMS, WEB |  |  | SMS, EML | SMS, WEB, APP |  |
| Womble 2004[82] | I | E.Diets | DL | B | Recommendation to record food intake daily during the first 16 weeks; 24h help desk; reminders; bi-weekly newsletter. | 12m | I | Yes | WBP, VC | WBP, EML, NEW |  | DIS, WBP | EML |  | VC | WEB |  |
| Womble 2004[82] | C | Weight loss manual | TAU+ | B | Weight loss manual: encouraged self-monitor food intake, increase PA and practice weight control behaviours | 12m | n/a | No |  |  |  |  |  |  |  |  |  |
| Young 2017[83] | I | The SHED-IT Weight Loss Maintenance Program | DO | B | After initial weight loss programme, advised to continue self-monitoring, high activity, eating breakfast, low tv watching. Weekly videos, biweekly motivational SMS | 6m | n/a | Yes | EML, VID | EML, VID |  |  | EML, VID, SMS |  |  | PED |  |
| Young 2017[83] | C | No additional resources | TAU | B | After initial WL programme, given no resources | 6m | n/a | No |  |  |  |  |  |  |  |  |  |
| EML, email; VID, video; SMS, short message service; PED, pedometer; WEB, website; WBP, web-based platform; VC, video conference; DIS, discussion board; AT, activity tracker; POD, podcast; SOC, social media; DIA, online diary; NEW, online newsletter; PDA, personal digital assistant; FBG, Facebook group; EHR, electronic health record; MMS, multimedia messaging; n/a, not applicable; DO, digital only; DL, digitally led; TAU, treatment as usual; TAU+, treatment as usual plus; WL, wait list; IVR, interactive voice response; PA, physical activity; MI, motivational interviewing; CBT, cognitive behavioural therapy; MT, mobile technology, MT+, mobile technology plus; WE, web-based plus email; WD, web-based plus peer support; WO, web-based only; BPBH, Be Positive Be Healthe; FG, Facebook group; PG, pamphlet group; CG, control group; TMC, telemedical coaching; C1, first control group; C2, second control group; TM, telemedical; TMC, telemedical coaching; aBWL, adapted behavioural weight loss; aBWL + BE, adapted behavioural weight loss plus behavioural economics; aBWL + SDT, adapted behavioural weight loss plus self-determination therapy; SI, Shape up + internet behavioural program; SII, Shape up + internet + incentives; CB Pro, cost-benefit approach with a professional coach; CB Peer, cost-benefit approach with a peer coach; mBHCSS, mobile health behaviour change support system; SWIM-C, Supporting Weight Management during Covid-19; ST, standard self-monitoring tools; NC, no counselling; AF, automated email feedback; HC, human email counselling; IWL, internet weight loss intervention only; IWL+PCP, internet weight loss intervention plus primary care provider; EUC, Enhanced usual care condition; HBCSS, Health behaviour change support system; SHG + HBCSS, Self-help guidance plus behaviour change support system; WW/WWO, Weight Watchers Online; WW+ES, Weight watchers online + experience Success; WWO+AL, Weight watchers online + ActiveLink; BT, Behavioural weight control treatment; BT+MI, Behavioural weight control treatment + motivational interviewing; SHIFT, Safety and Health Involvement for Truckers; iOTA, interactive obesity treatment approach (computer algorithm that assigned behaviour change goals); BC, bite counter device | | | | | | | | | | | | | | | | | |

Bibliography

1. Apinaniz A, Cobos-Campos R, Saez de Lafuente-Morinigo A, Parraza N, Aizpuru F, Perez I, et al. Effectiveness of randomized controlled trial of a mobile app to promote healthy lifestyle in obese and overweight patients. Fam Pract. 2019 Nov 18;36(6):699-705. PMID: 31093681. doi: 10.1093/fampra/cmz020.

2. Backman DR, Kohatsu ND, Padovani AJ, Dao C, Ritley D, Fleuret JE, et al. Achieving weight loss through a community-based, telewellness programme: A randomised controlled trial. Health Educ J. 2022;82(1):82-94. PMID: 2020396037. doi: 10.1177/00178969221139234.

3. Beleigoli A, Andrade AQ, Diniz MF, Ribeiro AL. Personalized Web-Based Weight Loss Behavior Change Program With and Without Dietitian Online Coaching for Adults With Overweight and Obesity: Randomized Controlled Trial. J Med Internet Res. 2020 Nov 5;22(11):e17494. PMID: 33151151. doi: 10.2196/17494.

4. Bennett GG, Foley P, Levine E, Whiteley J, Askew S, Steinberg DM, et al. Behavioral treatment for weight gain prevention among black women in primary care practice: a randomized clinical trial. JAMA Intern Med. 2013 Oct 28;173(19):1770-7. PMID: 23979005. doi: 10.1001/jamainternmed.2013.9263.

5. Lanpher MG, Askew S, Bennett GG. Health Literacy and Weight Change in a Digital Health Intervention for Women: A Randomized Controlled Trial in Primary Care Practice. J Health Commun. 2016;21 Suppl 1(Suppl):34-42. PMID: 27043756. doi: 10.1080/10810730.2015.1131773.

6. Berli C, Scholz U. Long-Term and Transfer Effects of an Action Control Intervention in Overweight Couples: A Randomized Controlled Trial Using Text Messages. Front Psychol. 2021;12:754488. PMID: 34899496. doi: 10.3389/fpsyg.2021.754488.

7. Braun TD, Olson K, Panza E, Lillis J, Schumacher L, Abrantes AM, et al. Internalized weight stigma in women with class III obesity: A randomized controlled trial of a virtual lifestyle modification intervention followed by a mindful self-compassion intervention. Obes Sci Pract. 2022 Dec;8(6):816-27. PMID: 36483124. doi: 10.1002/osp4.616.

8. Burke LE, Sereika SM, Parmanto B, Bizhanova Z, Kariuki JK, Cheng J, et al. Effect of tailored, daily feedback with lifestyle self-monitoring on weight loss: The SMARTER randomized clinical trial. Obesity (Silver Spring). 2022 Jan;30(1):75-84. PMID: 34898011. doi: 10.1002/oby.23321.

9. Burke LE, Conroy MB, Sereika SM, Elci OU, Styn MA, Acharya SD, et al. The effect of electronic self-monitoring on weight loss and dietary intake: a randomized behavioral weight loss trial. Obesity (Silver Spring). 2011 Feb;19(2):338-44. PMID: 20847736. doi: 10.1038/oby.2010.208.

10. Burke LE, Styn MA, Sereika SM, Conroy MB, Ye L, Glanz K, et al. Using mHealth technology to enhance self-monitoring for weight loss: a randomized trial. Am J Prev Med. 2012 Jul;43(1):20-6. PMID: 22704741. doi: 10.1016/j.amepre.2012.03.016.

11. Turk MW, Elci OU, Wang J, Sereika SM, Ewing LJ, Acharya SD, et al. Self-monitoring as a mediator of weight loss in the SMART randomized clinical trial. Int J Behav Med. 2013 Dec;20(4):556-61. PMID: 22936524. doi: 10.1007/s12529-012-9259-9.

12. Conroy MB, Yang K, Elci OU, Gabriel KP, Styn MA, Wang J, et al. Physical activity self-monitoring and weight loss: 6-month results of the SMART trial. Med Sci Sports Exerc. 2011 Aug;43(8):1568-74. PMID: 21200337. doi: 10.1249/MSS.0b013e31820b9395.

13. Carter MC, Burley VJ, Nykjaer C, Cade JE. Adherence to a smartphone application for weight loss compared to website and paper diary: pilot randomized controlled trial. J Med Internet Res. 2013 Apr 15;15(4):e32. PMID: 23587561. doi: 10.2196/jmir.2283.

14. Collins CE, Morgan PJ, Hutchesson MJ, Callister R. Efficacy of standard versus enhanced features in a Web-based commercial weight-loss program for obese adults, part 2: randomized controlled trial. J Med Internet Res. 2013 Jul 22;15(7):e140. PMID: 23876832. doi: 10.2196/jmir.2626.

15. Collins CE, Morgan PJ, Hutchesson MJ, Oldmeadow C, Barker D, Callister R. Efficacy of Web-Based Weight Loss Maintenance Programs: A Randomized Controlled Trial Comparing Standard Features Versus the Addition of Enhanced Personalized Feedback over 12 Months. Behav Sci (Basel). 2017 Nov 8;7(4). PMID: 29117105. doi: 10.3390/bs7040076.

16. Conroy MB, McTigue KM, Bryce CL, Tudorascu D, Gibbs BB, Arnold J, et al. Effect of Electronic Health Record-Based Coaching on Weight Maintenance: A Randomized Trial. Ann Intern Med. 2019 Dec 3;171(11):777-84. PMID: 31711168. doi: 10.7326/M18-3337.

17. Dombrowski SU, McDonald M, van der Pol M, Grindle M, Avenell A, Carroll P, et al. Game of Stones: feasibility randomised controlled trial of how to engage men with obesity in text message and incentive interventions for weight loss. BMJ Open. 2020 Feb 25;10(2):e032653. PMID: 32102807. doi: 10.1136/bmjopen-2019-032653.

18. Dombrowski SU, McDonald M, van der Pol M, Grindle M, Avenell A, Carroll P, et al. Text messaging and financial incentives to encourage weight loss in men with obesity: the Game of Stones feasibility RCT. Public Health Research 2020;8(11). doi: <https://dx.doi.org/10.3310/phr08110>.

19. Duncan MJ, Fenton S, Brown WJ, Collins CE, Glozier N, Kolt GS, et al. Efficacy of a Multi-component m-Health Weight-loss Intervention in Overweight and Obese Adults: A Randomised Controlled Trial. Int J Environ Res Public Health. 2020 Aug 26;17(17). PMID: 32859100. doi: 10.3390/ijerph17176200.

20. Dunn CG, Turner-McGrievy GM, Wilcox S, Hutto B. Dietary Self-Monitoring Through Calorie Tracking but Not Through a Digital Photography App Is Associated with Significant Weight Loss: The 2SMART Pilot Study-A 6-Month Randomized Trial. J Acad Nutr Diet. 2019 Sep;119(9):1525-32. PMID: 31155474. doi: 10.1016/j.jand.2019.03.013.

21. Eisenhauer CM, Brito F, Kupzyk K, Yoder A, Almeida F, Beller RJ, et al. Mobile health assisted self-monitoring is acceptable for supporting weight loss in rural men: a pragmatic randomized controlled feasibility trial. BMC Public Health. 2021 Aug 18;21(1):1568. PMID: 34407782. doi: 10.1186/s12889-021-11618-7.

22. Falkenhain K, Locke SR, Lowe DA, Reitsma NJ, Lee T, Singer J, et al. Keyto app and device versus WW app on weight loss and metabolic risk in adults with overweight or obesity: A randomized trial. Obesity (Silver Spring). 2021 Oct;29(10):1606-14. PMID: 34124856. doi: 10.1002/oby.23242.

23. Gemesi K, Winkler S, Schmidt-Tesch S, Schederecker F, Hauner H, Holzapfel C. Efficacy of an app-based multimodal lifestyle intervention on body weight in persons with obesity: results from a randomized controlled trial. Int J Obes (Lond). 2024 Jan;48(1):118-26. PMID: 38017117. doi: 10.1038/s41366-023-01415-0.

24. Gold BC, Burke S, Pintauro S, Buzzell P, Harvey-Berino J. Weight loss on the web: A pilot study comparing a structured behavioral intervention to a commercial program. Obesity (Silver Spring). 2007 Jan;15(1):155-64. PMID: 17228043. doi: 10.1038/oby.2007.520.

25. Haapala I, Barengo NC, Biggs S, Surakka L, Manninen P. Weight loss by mobile phone: a 1-year effectiveness study. Public Health Nutr. 2009 Dec;12(12):2382-91. PMID: 19323865. doi: 10.1017/S1368980009005230.

26. Hageman PA, Pullen CH, Hertzog M, Pozehl B, Eisenhauer C, Boeckner LS. Web-Based Interventions Alone or Supplemented with Peer-Led Support or Professional Email Counseling for Weight Loss and Weight Maintenance in Women from Rural Communities: Results of a Clinical Trial. J Obes. 2017;2017:1602627. PMID: 28480078. doi: 10.1155/2017/1602627.

27. Hesseldal L, Christensen JR, Olesen TB, Olsen MH, Jakobsen PR, Laursen DH, et al. Long-term Weight Loss in a Primary Care-Anchored eHealth Lifestyle Coaching Program: Randomized Controlled Trial. J Med Internet Res. 2022 Sep 23;24(9):e39741. PMID: 36149735. doi: 10.2196/39741.

28. Christensen JR, Hesseldal L, Olesen TB, Olsen MH, Jakobsen PR, Laursen DH, et al. Long-term weight loss in a 24-month primary care-anchored telehealth lifestyle coaching program: Randomized controlled trial. J Telemed Telecare. 2022 Dec;28(10):764-70. PMID: 36346936. doi: 10.1177/1357633X221123411.

29. Hutchesson MJ, Callister R, Morgan PJ, Pranata I, Clarke ED, Skinner G, et al. A Targeted and Tailored eHealth Weight Loss Program for Young Women: The Be Positive Be Healthe Randomized Controlled Trial. Healthcare (Basel). 2018 May 2;6(2). PMID: 29724054. doi: 10.3390/healthcare6020039.

30. Jane M, Hagger M, Foster J, Ho S, Kane R, Pal S. Effects of a weight management program delivered by social media on weight and metabolic syndrome risk factors in overweight and obese adults: A randomised controlled trial. PLoS One. 2017;12(6):e0178326. PMID: 28575048. doi: 10.1371/journal.pone.0178326.

31. Jane M, Foster J, Hagger M, Ho S, Kane R, Pal S. Psychological effects of belonging to a Facebook weight management group in overweight and obese adults: Results of a randomised controlled trial. Health Soc Care Community. 2018 May 18. PMID: 29774616. doi: 10.1111/hsc.12584.

32. Joseph RP, Todd M, Ainsworth BE, Vega-Lopez S, Adams MA, Hollingshead K, et al. Smart Walk: A Culturally Tailored Smartphone-Delivered Physical Activity Intervention for Cardiometabolic Risk Reduction among African American Women. Int J Environ Res Public Health. 2023 Jan 5;20(2). PMID: 36673756. doi: 10.3390/ijerph20021000.

33. Kempf K, Rohling M, Martin S, Schneider M. Telemedical coaching for weight loss in overweight employees: a three-armed randomised controlled trial. BMJ Open. 2019 Apr 11;9(4):e022242. PMID: 30975666. doi: 10.1136/bmjopen-2018-022242.

34. Kempf K, Rohling M, Stichert M, Fischer G, Boschem E, Konner J, et al. Telemedical Coaching Improves Long-Term Weight Loss in Overweight Persons: A Randomized Controlled Trial. Int J Telemed Appl. 2018;2018:7530602. PMID: 30271433. doi: 10.1155/2018/7530602.

35. Keshavarz M, Senechal M, Bouchard DR. Online Circuit Training Increases Adherence to Physical Activity: A Randomized Controlled Trial of Men with Obesity. Med Sci Sports Exerc. 2023 Dec 1;55(12):2308-15. PMID: 37535330. doi: 10.1249/MSS.0000000000003270.

36. Kharmats AY, Wang C, Fuentes L, Hu L, Kline T, Welding K, et al. Monday-focused tailored rapid interactive mobile messaging for weight management 2 (MTRIMM2): results from a randomized controlled trial. Mhealth. 2022;8:1. PMID: 35178432. doi: 10.21037/mhealth-21-3.

37. Kim M, Kim Y, Go Y, Lee S, Na M, Lee Y, et al. Multidimensional Cognitive Behavioral Therapy for Obesity Applied by Psychologists Using a Digital Platform: Open-Label Randomized Controlled Trial. JMIR Mhealth Uhealth. 2020 Apr 30;8(4):e14817. PMID: 32352391. doi: 10.2196/14817.

38. Kohl J, Brame J, Centner C, Wurst R, Fuchs R, Sehlbrede M, et al. Effects of a Web-Based Lifestyle Intervention on Weight Loss and Cardiometabolic Risk Factors in Adults With Overweight and Obesity: Randomized Controlled Clinical Trial. J Med Internet Res. 2023 Jun 27;25:e43426. PMID: 37368484. doi: 10.2196/43426.

39. Kurtzman GW, Day SC, Small DS, Lynch M, Zhu J, Wang W, et al. Social Incentives and Gamification to Promote Weight Loss: The LOSE IT Randomized, Controlled Trial. J Gen Intern Med. 2018 Oct;33(10):1669-75. PMID: 30003481. doi: 10.1007/s11606-018-4552-1.

40. Laing BY, Mangione CM, Tseng CH, Leng M, Vaisberg E, Mahida M, et al. Effectiveness of a smartphone application for weight loss compared with usual care in overweight primary care patients: a randomized, controlled trial. Ann Intern Med. 2014 Nov 18;161(10 Suppl):S5-12. PMID: 25402403. doi: 10.7326/M13-3005.

41. LaRose JG, Leahey TM, Lanoye A, Bean MK, Fava JL, Tate DF, et al. Effect of a Lifestyle Intervention on Cardiometabolic Health Among Emerging Adults: A Randomized Clinical Trial. JAMA Netw Open. 2022 Sep 1;5(9):e2231903. PMID: 36121656. doi: 10.1001/jamanetworkopen.2022.31903.

42. Leahey TM, Subak LL, Fava J, Schembri M, Thomas G, Xu X, et al. Benefits of adding small financial incentives or optional group meetings to a web-based statewide obesity initiative. Obesity (Silver Spring). 2015 Jan;23(1):70-6. PMID: 25384463. doi: 10.1002/oby.20937.

43. Leahey TM, Fava JL, Seiden A, Fernandes D, Doyle C, Kent K, et al. A randomized controlled trial testing an Internet delivered cost-benefit approach to weight loss maintenance. Prev Med. 2016 Nov;92:51-7. PMID: 27095323. doi: 10.1016/j.ypmed.2016.04.013.

44. Little P, Stuart B, Hobbs FR, Kelly J, Smith ER, Bradbury KJ, et al. An internet-based intervention with brief nurse support to manage obesity in primary care (POWeR+): a pragmatic, parallel-group, randomised controlled trial. Lancet Diabetes Endocrinol. 2016 Oct;4(10):821-8. PMID: 27474214. doi: 10.1016/S2213-8587(16)30099-7.

45. Little P, Stuart B, Hobbs FR, Kelly J, Smith ER, Bradbury KJ, et al. Randomised controlled trial and economic analysis of an internet-based weight management programme: POWeR+ (Positive Online Weight Reduction). Health Technol Assess. 2017 Jan;21(4):1-62. PMID: 28122658. doi: 10.3310/hta21040.

46. Markkanen JO, Oikarinen N, Savolainen MJ, Merikallio H, Nyman V, Salminen V, et al. Mobile health behaviour change support system as independent treatment tool for obesity: a randomized controlled trial. Int J Obes (Lond). 2024 Mar;48(3):376-83. PMID: 38062218. doi: 10.1038/s41366-023-01426-x.

47. McConnon A, Kirk SF, Cockroft JE, Harvey EL, Greenwood DC, Thomas JD, et al. The Internet for weight control in an obese sample: results of a randomised controlled trial. BMC Health Serv Res. 2007 Dec 19;7:206. PMID: 18093289. doi: 10.1186/1472-6963-7-206.

48. Morgan PJ, Lubans DR, Collins CE, Warren JM, Callister R. The SHED-IT randomized controlled trial: evaluation of an Internet-based weight-loss program for men. Obesity (Silver Spring). 2009 Nov;17(11):2025-32. PMID: 19343018. doi: 10.1038/oby.2009.85.

49. Morgan PJ, Lubans DR, Collins CE, Warren JM, Callister R. 12-month outcomes and process evaluation of the SHED-IT RCT: an internet-based weight loss program targeting men. Obesity (Silver Spring). 2011 Jan;19(1):142-51. PMID: 20523304. doi: 10.1038/oby.2010.119.

50. Morgan PJ, Callister R, Collins CE, Plotnikoff RC, Young MD, Berry N, et al. The SHED-IT community trial: a randomized controlled trial of internet- and paper-based weight loss programs tailored for overweight and obese men. Ann Behav Med. 2013 Apr;45(2):139-52. PMID: 23129021. doi: 10.1007/s12160-012-9424-z.

51. Blomfield RL, Collins CE, Hutchesson MJ, Young MD, Jensen ME, Callister R, et al. Impact of self-help weight loss resources with or without online support on the dietary intake of overweight and obese men: the SHED-IT randomised controlled trial. Obes Res Clin Pract. 2014 Sep-Oct;8(5):e476-87. PMID: 25263837. doi: 10.1016/j.orcp.2013.09.004.

52. Mueller J, Richards R, Jones RA, Whittle F, Woolston J, Stubbings M, et al. Supporting Weight Management during COVID-19: A Randomized Controlled Trial of a Web-Based, ACT-Based, Guided Self-Help Intervention. Obes Facts. 2022;15(4):550-9. PMID: 35417910. doi: 10.1159/000524031.

53. Mueller J, Richards R, Jones RA, Whittle F, Woolston J, Stubbings M, et al. Supporting Weight Management during COVID-19 (SWiM-C): twelve-month follow-up of a randomised controlled trial of a web-based, ACT-based, guided self-help intervention. Int J Obes (Lond). 2023 Jan;47(1):51-9. PMID: 36369513. doi: 10.1038/s41366-022-01232-x.

54. Olson R, Wipfli B, Thompson SV, Elliot DL, Anger WK, Bodner T, et al. Weight Control Intervention for Truck Drivers: The SHIFT Randomized Controlled Trial, United States. Am J Public Health. 2016 Sep;106(9):1698-706. PMID: 27463067. doi: 10.2105/AJPH.2016.303262.

55. Wipfli B, Hanson G, Anger K, Elliot DL, Bodner T, Stevens V, et al. Process Evaluation of a Mobile Weight Loss Intervention for Truck Drivers. Saf Health Work. 2019 Mar;10(1):95-102. PMID: 30949387. doi: 10.1016/j.shaw.2018.08.002.

56. Patel ML, Hopkins CM, Brooks TL, Bennett GG. Comparing Self-Monitoring Strategies for Weight Loss in a Smartphone App: Randomized Controlled Trial. JMIR Mhealth Uhealth. 2019 Feb 28;7(2):e12209. PMID: 30816851. doi: 10.2196/12209.

57. Patrick K, Calfas KJ, Norman GJ, Rosenberg D, Zabinski MF, Sallis JF, et al. Outcomes of a 12-month web-based intervention for overweight and obese men. Ann Behav Med. 2011 Dec;42(3):391-401. PMID: 21822750. doi: 10.1007/s12160-011-9296-7.

58. Rogers RJ, Lang W, Barone Gibbs B, Davis KK, Burke LE, Kovacs SJ, et al. Applying a technology-based system for weight loss in adults with obesity. Obes Sci Pract. 2016 Mar;2(1):3-12. PMID: 27812375. doi: 10.1002/osp4.18.

59. Ross KM, Wing RR. Impact of newer self-monitoring technology and brief phone-based intervention on weight loss: A randomized pilot study. Obesity (Silver Spring). 2016 Aug;24(8):1653-9. PMID: 27367614. doi: 10.1002/oby.21536.

60. Roth L, Ordnung M, Forkmann K, Mehl N, Horstmann A. A randomized-controlled trial to evaluate the app-based multimodal weight loss program zanadio for patients with obesity. Obesity (Silver Spring). 2023 May;31(5):1300-10. PMID: 37140392. doi: 10.1002/oby.23744.

61. Shapiro JR, Koro T, Doran N, Thompson S, Sallis JF, Calfas K, et al. Text4Diet: a randomized controlled study using text messaging for weight loss behaviors. Prev Med. 2012 Nov;55(5):412-7. PMID: 22944150. doi: 10.1016/j.ypmed.2012.08.011.

62. Shuger SL, Barry VW, Sui X, McClain A, Hand GA, Wilcox S, et al. Electronic feedback in a diet- and physical activity-based lifestyle intervention for weight loss: a randomized controlled trial. Int J Behav Nutr Phys Act. 2011 May 18;8:41. PMID: 21592351. doi: 10.1186/1479-5868-8-41.

63. Silina V, Tessma MK, Senkane S, Krievina G, Bahs G. Text messaging (SMS) as a tool to facilitate weight loss and prevent metabolic deterioration in clinically healthy overweight and obese subjects: a randomised controlled trial. Scand J Prim Health Care. 2017 Sep;35(3):262-70. PMID: 28812403. doi: 10.1080/02813432.2017.1358435.

64. Simpson SA, Matthews L, Pugmire J, McConnachie A, McIntosh E, Coulman E, et al. An app-, web- and social support-based weight loss intervention for adults with obesity: the HelpMeDoIt! feasibility RCT. Public Health Research. 2020;8(3). doi: <https://dx.doi.org/10.3310/phr08030>.

65. Simpson SA, Matthews L, Pugmire J, McConnachie A, McIntosh E, Coulman E, et al. An app-, web- and social support-based weight loss intervention for adults with obesity: the 'HelpMeDoIt!' feasibility randomised controlled trial. Pilot Feasibility Stud. 2020;6:133. PMID: 32968544. doi: 10.1186/s40814-020-00656-4.

66. Sniehotta FF, Evans EH, Sainsbury K, Adamson A, Batterham A, Becker F, et al. Behavioural intervention for weight loss maintenance versus standard weight advice in adults with obesity: A randomised controlled trial in the UK (NULevel Trial). PLoS Med. 2019 May;16(5):e1002793. PMID: 31063507. doi: 10.1371/journal.pmed.1002793.

67. Steinberg DM, Levine EL, Askew S, Foley P, Bennett GG. Daily text messaging for weight control among racial and ethnic minority women: randomized controlled pilot study. J Med Internet Res. 2013 Nov 18;15(11):e244. PMID: 24246427. doi: 10.2196/jmir.2844.

68. Tate DF, Wing RR, Winett RA. Using Internet technology to deliver a behavioral weight loss program. JAMA. 2001 Mar 7;285(9):1172-7. PMID: 11231746. doi: 10.1001/jama.285.9.1172.

69. Tate DF, Jackvony EH, Wing RR. A randomized trial comparing human e-mail counseling, computer-automated tailored counseling, and no counseling in an Internet weight loss program. Arch Intern Med. 2006;166(15):1620-5.

70. Tate DF, Kraschnewski JL, Martinez C, Diamond M, Veldheer S, Hwang KO, et al. A cluster-randomized controlled trial of automated internet weight-loss programs in primary care: Role of automated provider feedback. Obesity (Silver Spring). 2022 Dec;30(12):2363-75. PMID: 36416000. doi: 10.1002/oby.23506.

71. Teeriniemi AM, Salonurmi T, Jokelainen T, Vahanikkila H, Alahaivala T, Karppinen P, et al. A randomized clinical trial of the effectiveness of a Web-based health behaviour change support system and group lifestyle counselling on body weight loss in overweight and obese subjects: 2-year outcomes. J Intern Med. 2018 Nov;284(5):534-45. PMID: 29974563. doi: 10.1111/joim.12802.

72. Thomas JG, Goldstein CM, Bond DS, Hadley W, Tuerk PW. Web-based virtual reality to enhance behavioural skills training and weight loss in a commercial online weight management programme: The Experience Success randomized trial. Obes Sci Pract. 2020 Dec;6(6):587-95. PMID: 33354337. doi: 10.1002/osp4.451.

73. Thomas JG, Raynor HA, Bond DS, Luke AK, Cardoso CC, Foster GD, et al. Weight loss in Weight Watchers Online with and without an activity tracking device compared to control: A randomized trial. Obesity (Silver Spring). 2017 Jun;25(6):1014-21. PMID: 28437597. doi: 10.1002/oby.21846.

74. Turner-McGrievy G, Tate D. Tweets, Apps, and Pods: Results of the 6-month Mobile Pounds Off Digitally (Mobile POD) randomized weight-loss intervention among adults. J Med Internet Res. 2011 Dec 20;13(4):e120. PMID: 22186428. doi: 10.2196/jmir.1841.

75. Turner-McGrievy GM, Wilcox S, Boutte A, Hutto BE, Singletary C, Muth ER, et al. The Dietary Intervention to Enhance Tracking with Mobile Devices (DIET Mobile) Study: A 6-Month Randomized Weight Loss Trial. Obesity (Silver Spring). 2017 Aug;25(8):1336-42. PMID: 28600833. doi: 10.1002/oby.21889.

76. van Genugten L, van Empelen P, Boon B, Borsboom G, Visscher T, Oenema A. Results from an online computer-tailored weight management intervention for overweight adults: randomized controlled trial. J Med Internet Res. 2012 Mar 14;14(2):e44. PMID: 22417813. doi: 10.2196/jmir.1901.

77. van Wier MF, Ariens GA, Dekkers JC, Hendriksen IJ, Smid T, van Mechelen W. Phone and e-mail counselling are effective for weight management in an overweight working population: a randomized controlled trial. BMC Public Health. 2009 Jan 9;9:6. PMID: 19134171. doi: 10.1186/1471-2458-9-6.

78. van Wier MF, Dekkers JC, Hendriksen IJ, Heymans MW, Ariens GA, Pronk NP, et al. Effectiveness of phone and e-mail lifestyle counseling for long term weight control among overweight employees. J Occup Environ Med. 2011 Jun;53(6):680-6. PMID: 21654441. doi: 10.1097/JOM.0b013e31821f2bbb.

79. van Wier MF, Dekkers JC, Bosmans JE, Heymans MW, Hendriksen IJ, Pronk NP, et al. Economic evaluation of a weight control program with e-mail and telephone counseling among overweight employees: a randomized controlled trial. Int J Behav Nutr Phys Act. 2012 Sep 11;9:112. PMID: 22967224. doi: 10.1186/1479-5868-9-112.

80. West DS, Harvey JR, Krukowski RA, Prewitt TE, Priest J, Ashikaga T. Do individual, online motivational interviewing chat sessions enhance weight loss in a group-based, online weight control program? Obesity (Silver Spring). 2016 Nov;24(11):2334-40. PMID: 27616628. doi: 10.1002/oby.21645.

81. West DS, Stansbury M, Krukowski RA, Harvey J. Enhancing group-based internet obesity treatment: A pilot RCT comparing video and text-based chat. Obes Sci Pract. 2019 Dec;5(6):513-20. PMID: 31890241. doi: 10.1002/osp4.371.

82. Womble LG, Wadden TA, McGuckin BG, Sargent SL, Rothman RA, Krauthamer-Ewing ES. A randomized controlled trial of a commercial internet weight loss program. Obes Res. 2004 Jun;12(6):1011-8. PMID: 15229342. doi: 10.1038/oby.2004.124.

83. Young MD, Callister R, Collins CE, Plotnikoff RC, Aguiar EJ, Morgan PJ. Efficacy of a gender-tailored intervention to prevent weight regain in men over 3 years: A weight loss maintenance RCT. Obesity (Silver Spring). 2017 Jan;25(1):56-65. PMID: 27925437. doi: 10.1002/oby.21696.
