# Supplementary material for: Evaluation of the Aspects of Digital Interventions That Successfully Support Weight Loss: Systematic Review With Component Network Meta-Analysis
Source: J Med Internet Res. 2025 May 22;27:e65443. doi: 10.2196/65443 (PMC12141966; doi:10.2196/65443)
Supplement: Multimedia Appendix 6 [file jmir_v27i1e65443_app6.docx]

### Multimedia Appendix 6. Component network meta-analysis model diagnostics.

| **Outcome**​ | **Timeframe**​ | **Studies**​ | **τ^2^**​ | **I^2^ (%)**​ | **Q**​ | **p-value**​ |
| --- | --- | --- | --- | --- | --- | --- |
| Absolute weight loss | At 6 months​ | 43​ | 1.0176​ | 61.1​ | 105.37​ | <0.0001​ |
| Absolute weight loss | At 12 months​ | 28​ | 0.8457​ | 57.2​ | 53.7​ | 0.0003​ |
| BMI​ | At 6 months​ | 28​ | 0.0591​ | 29.1​ | 32.46​ | 0.09​ |
| BMI​ | At 12 months​ | 14​ | 0.0642​ | 30.1​ | 10.02​ | 0.19​ |
| Responder​ | At 6 months​ | 20​ | 0.1132​ | 50.1​ | 30.09​ | 0.01​ |
| Responder​ | At 12 months​ | 15​ | 0.223​ | 59.2​ | 22.09​ | 0.009​ |
| Pct loss​ | At 6 months​ | 20​ | 1.226​ | 56​ | 31.84​ | 0.004​ |
| Pct loss​ | At 12 months​ | 14​ | 5.9317​ | 82.9​ | 52.49​ | <0.0001​ |
