# Supplementary material for: Evaluation of the Aspects of Digital Interventions That Successfully Support Weight Loss: Systematic Review With Component Network Meta-Analysis
Source: J Med Internet Res. 2025 May 22;27:e65443. doi: 10.2196/65443 (PMC12141966; doi:10.2196/65443)
Supplement: Multimedia Appendix 7 [file jmir_v27i1e65443_app7.docx]

**Multimedia Appendix 7.** Extracted effect sizes for all trial arms included in the component network meta-analysis.

| **Study ID** | **Arm 1** | **Arm 2** | **Outcome category** | **Outcome notes** | **Time category** | **Time notes** | **Units** | **MD** | **MD LCI** | **MD UCI** | **MD SE** |
| --- | --- | --- | --- | --- | --- | --- | --- | --- | --- | --- | --- |
| Apiñaniz2019[1] | Intervention | Control | Absolute weight loss |  | Around 6 | At 6 months (only follow-up presented) | kg | 0.70 |  |  | 3.15 |
| Backman2023[2] | KFP | Control | Absolute weight loss |  | Around 6 | 25 weeks | lbs | -7.72 | -13.02 | -2.42 | 2.70 |
|  | KFP | Control | BMI |  | Around 6 | 25 weeks |  | -1.24 | -2.10 | -0.38 | 0.44 |
| Beleigoli2020[3] | Platform | WL | Absolute weight loss |  | Around 6 | 24 weeks | kg | -0.42 |  |  | 0.35 |
|  | Platform plus coaching | WL | Absolute weight loss |  | Around 6 | 24 weeks | kg | -0.91 |  |  | 0.24 |
|  | Platform plus coaching | Platform | Absolute weight loss |  | Around 6 | 24 weeks | kg | -0.49 |  |  | 0.33 |
|  | Platform | WL | BMI |  | Around 6 | 24 weeks |  | -0.14 |  |  | 0.13 |
|  | Platform plus coaching | WL | BMI |  | Around 6 | 24 weeks |  | -0.32 |  |  | 0.09 |
|  | Platform plus coaching | Platform | BMI |  | Around 6 | 24 weeks |  | -0.18 |  |  | 0.71 |
|  | Platform | WL | Responder | At least 5% loss | Around 6 | 24 weeks |  |  |  |  |  |
|  | Platform plus coaching | WL | Responder | At least 5% loss | Around 6 | 24 weeks |  |  |  |  |  |
|  | Platform plus coaching | Platform | Responder | At least 5% loss | Around 6 | 24 weeks |  |  |  |  |  |
| Berli2021[4] | Intervention | Control | BMI |  | Around 6 | At 6 months |  | 1.12 |  |  | 1.02 |
|  | Intervention | Control | Anthrop | Waist to hip ratio | Around 6 | At 6 months |  | 0.00 |  |  | 0.02 |
| Braun2022[5] | MSC | Control | Absolute weight loss |  | Around 6 | At 6 months | kg | 0.80 |  |  | 0.57 |
| Burke2011,[6]2012;[7]Turk2013;[8] Conroy2011[9] | PDA+FB | PDA | Pct loss |  | Around 6 | At 6 months |  | -1.80 |  |  | 1.16 |
|  | PDA+FB | PDA | Responder | At least 5% loss | Around 6 | At 6 months |  |  |  |  |  |
|  | PDA+FB | PDA | Anthrop | Waist circumference percentage loss | Around 6 | At 6 months |  | -1.40 |  |  | 0.95 |
|  | PDA+FB | PDA | Pct loss |  | Around 12 | 12 months |  | -1.98 |  |  | 1.27 |
|  | PDA+FB | PDA | Pct loss |  | Longer than 12 | 18 months |  | -1.80 |  |  | 1.29 |
|  | PDA+FB | PDA | Pct loss |  | Longer than 12 | 24 months |  | -0.94 |  |  | 1.29 |
|  | PDA+FB | PDA | Absolute weight loss |  | Longer than 12 | 24 months | kg | -0.99 |  |  | 1.36 |
| Burke2022,2022[10, 11] | SM+FB | SM | Pct loss |  | Around 6 | At 6 months |  | 0.04 | -0.91 | 0.99 | 0.48 |
|  | SM+FB | SM | Absolute weight loss |  | Around 6 | At 6 months | kg | 0.04 | -0.91 | 0.99 | 0.48 |
|  | SM+FB | SM | Responder | At least 5% loss | Around 6 | At 6 months |  |  |  |  |  |
|  | SM+FB | SM | Responder | At least 3% loss | Around 6 | At 6 months |  |  |  |  |  |
|  | SM+FB | SM | BMI |  | Around 6 | At 6 months |  | 0.59 | -0.26 | 1.44 | 0.43 |
|  | SM+FB | SM | Anthrop | Body fat % | Around 6 | At 6 months |  | 0.62 | -1.01 | 2.26 |  |
|  | SM+FB | SM | Anthrop | Female waist circumference (cm) | Around 6 | At 6 months |  | -0.11 | -2.87 | 2.65 |  |
|  | SM+FB | SM | Anthrop | Male waist circumference (cm) | Around 6 | At 6 months |  | 0.05 | -5.53 | 5.64 |  |
|  | SM+FB | SM | Absolute weight loss |  | Around 12 | At 12 months | kg | 0.36 | -0.70 | 1.43 | 0.54 |
|  | SM+FB | SM | Pct loss |  | Around 12 | At 12 months |  | 0.27 | -1.03 | 1.57 | 0.66 |
|  | SM+FB | SM | Responder | At least 5% loss | Around 12 | At 12 months |  |  |  |  |  |
| Carter2013[12] | smartphone | diary | Absolute weight loss |  | Around 6 | At 6 months | kg | -1.70 |  |  | 1.09 |
|  | website | diary | Absolute weight loss |  | Around 6 | At 6 months | kg | 1.60 |  |  | 1.08 |
|  | smartphone | website | Absolute weight loss |  | Around 6 | At 6 months | kg | -3.30 | -5.40 | -1.2 | 1.07 |
|  | smartphone | diary | BMI |  | Around 6 | At 6 months |  | -0.60 |  |  | 0.42 |
|  | website | diary | BMI |  | Around 6 | At 6 months |  | 0.50 |  |  | 0.38 |
|  | smartphone | website | BMI |  | Around 6 | At 6 months |  | -1.10 |  |  | 0.36 |
|  | smartphone | diary | Anthrop | Body fat % | Around 6 | At 6 months |  | -0.40 |  |  | 0.36 |
|  | website | diary | Anthrop | Body fat % | Around 6 | At 6 months |  | 0.40 |  |  | 0.36 |
|  | smartphone | website | Anthrop | Body fat % | Around 6 | At 6 months |  | -0.80 |  |  | 0.32 |
|  | smartphone | diary | Responder | At least 5% loss | Around 6 | At 6 months |  |  |  |  |  |
|  | website | diary | Responder | At least 5% loss | Around 6 | At 6 months |  |  |  |  |  |
|  | smartphone | website | Responder | At least 5% loss | Around 6 | At 6 months |  |  |  |  |  |
| Collins2013[13] | Enhanced | Basic | Absolute weight loss |  | Around 6 | At 6 months | kg | -0.70 | -2.00 | 0.6 | 0.66 |
|  | Enhanced | Basic | Pct loss |  | Around 6 | At 6 months |  | -0.71 | -2.02 | 0.59 | 0.67 |
|  | Enhanced | Basic | Responder | At least 5% loss | Around 6 | At 6 months |  |  |  |  |  |
|  | Enhanced | Basic | BMI |  | Around 6 | At 6 months |  | -0.20 | -0.60 | 0.2 | 0.20 |
|  | Enhanced | Basic | Anthrop | Waist circumference at umbilicus (cm) | Around 6 | At 6 months |  | -0.90 | -2.30 | 0.5 |  |
|  | Enhanced | Basic | Anthrop | Waist circumference at narrowest point (cm) | Around 6 | At 6 months |  | -0.90 | -2.20 | 0.3 |  |
|  | Enhanced | Basic | Anthrop | Waist to height ratio, umbilicus | Around 6 | At 6 months |  | 0.00 | -0.01 | 0.01 |  |
|  | Enhanced | Basic | Anthrop | Waist to height ratio, narrowest point | Around 6 | At 6 months |  | 0.00 | -0.01 | 0.01 |  |
| Collins2017[14] | Enhanced | Basic | Absolute weight loss |  | Around 12 | At 12 months | kg | -0.15 | -1.39 | 1.09 | 0.63 |
|  | Enhanced | Basic | BMI |  | Around 12 | At 12 months |  | -0.02 | -0.50 | 0.46 | 0.24 |
|  | Enhanced | Basic | Anthrop | Waist circumference at umbilicus (cm) | Around 12 | At 12 months |  | -0.76 | -2.16 | 0.63 |  |
|  | Enhanced | Basic | Anthrop | Waist circumference at narrowest point (cm) | Around 12 | At 12 months |  | -0.11 | -1.35 | 1.12 |  |
|  | Enhanced | Basic | Anthrop | Waist to height ratio, umbilicus | Around 12 | At 12 months |  | -0.01 | -0.01 | 0.01 |  |
|  | Enhanced | Basic | Anthrop | Waist to height ratio, narrowest point | Around 12 | At 12 months |  | 0.00 | -0.01 | 0.01 |  |
| Conroy2019[15] | Coaching | Tracking | Absolute weight loss |  | Around 6 | At 6 months | kg | -1.88 | -3.55 | -0.2 | 0.85 |
|  | Coaching | Tracking | BMI |  | Around 6 | At 6 months |  | -0.65 | -1.26 | -0.04 | 0.31 |
|  | Coaching | Tracking | Anthrop | Waist circumference (cm) | Around 6 | At 6 months |  | -2.22 | -4.12 | -0.32 | 0.97 |
|  | Coaching | Tracking | Absolute weight loss |  | Around 12 | At 12 months | kg | -1.31 | -3.00 | 0.39 | 0.86 |
|  | Coaching | Tracking | BMI |  | Around 12 | At 12 months |  | -0.46 | -1.08 | 0.15 | 0.31 |
|  | Coaching | Tracking | Anthrop | Waist circumference (cm) | Around 12 | At 12 months |  | -2.07 | -4.01 | -0.12 | 0.99 |
|  | Coaching | Tracking | Absolute weight loss |  | Longer than 12 | At 24 months | kg | -2.86 | -4.60 | -1.11 | 0.89 |
|  | Coaching | Tracking | BMI |  | Longer than 12 | At 24 months |  | -1.00 | -1.62 | -0.37 | 0.32 |
|  | Coaching | Tracking | Anthrop | Waist circumference (cm) | Longer than 12 | At 24 months |  | -2.49 | -4.53 | -0.44 | 1.04 |
|  | Coaching | Tracking | Anthrop | Waist circumference (cm) | Longer than 12 | At 30 months |  | -3.21 | -5.17 | -1.25 |  |
| Duncan2020,[16] | Traditional/enhanced | WL | Absolute weight loss |  | Around 6 | At 6 months | kg | -1.33 | -5.38 | 2.72 | 2.07 |
|  | Traditional/enhanced | WL | Absolute weight loss |  | Around 12 | At 12 months | kg | -2.61 | -8.42 | 3.2 | 2.96 |
|  | Traditional/enhanced | WL | Anthrop | Waist circumference (cm) | Around 6 | At 6 months |  | -1.77 | -5.56 | 2.02 |  |
|  | Traditional/enhanced | WL | Anthrop | Waist circumference (cm) | Around 12 | At 12 months |  | -2.77 | -7.73 | 2.19 |  |
| Eisenhauer2021[17] | MT+ | MT | Absolute weight loss |  | Around 6 | At 6 months | kg | -2.89 |  |  | 1.87 |
|  | MT+ | MT | BMI |  | Around 6 | At 6 months |  | -0.81 |  |  | 0.57 |
|  | MT+ | MT | Pct loss |  | Around 6 | At 6 months |  | -2.08 |  |  | 1.53 |
|  | MT+ | MT | Responder | At least 5% loss | Around 6 | At 6 months |  |  |  |  |  |
|  | MT+ | MT | Responder | At least 3% loss | Around 6 | At 6 months |  |  |  |  |  |
| Gemesi2024[18] | ADHOC | EXPECT | Absolute weight loss |  | Around 6 | At 24 weeks | kg | -1.00 |  |  | 0.57 |
|  | ADHOC | EXPECT | Pct loss |  | Around 6 | At 24 weeks |  | -1.10 |  |  | 0.55 |
|  | ADHOC | EXPECT | Responder | At least 3% loss | Around 6 | At 24 weeks |  |  |  |  |  |
|  | ADHOC | EXPECT | Responder | At least 5% loss | Around 6 | At 24 weeks |  |  |  |  |  |
|  | ADHOC | EXPECT | Anthrop | Fat mass (%) | Around 6 | At 24 weeks |  | -0.65 |  |  | 0.42 |
|  | ADHOC | EXPECT | Anthrop | Fat mass (kg) | Around 6 | At 24 weeks |  | -1.27 |  |  | 0.63 |
|  | ADHOC | EXPECT | Anthrop | Fat free mass (kg) | Around 6 | At 24 weeks |  | -0.30 |  |  | 0.35 |
| Gold2007[19] | Vtrim | E.Diets | Absolute weight loss |  | Around 6 | At 6 months | kg | -3.50 |  |  | 1.25 |
|  | Vtrim | E.Diets | Pct loss |  | Around 6 | At 6 months |  | -3.70 |  |  | 1.26 |
|  | Vtrim | E.Diets | Absolute weight loss |  | Around 12 | At 12 months | kg | -2.50 |  |  | 1.18 |
|  | Vtrim | E.Diets | Pct loss |  | Around 12 | At 12 months |  | -2.70 |  |  | 1.19 |
|  | Vtrim | E.Diets | Responder | At least 5% loss | Around 12 | At 12 months |  |  |  |  |  |
| Haapala2009[20] | EG | CG | Absolute weight loss |  | Around 12 | At 12 months | kg | -2.40 |  |  | 0.83 |
|  | EG | CG | Pct loss |  | Around 12 | At 12 months |  | -4.10 |  |  | 1.36 |
|  | EG | CG | Anthrop | Waist circumference (cm) | Around 12 | At 12 months |  | -2.90 |  |  | 1.01 |
|  | EG | CG | Responder | At least 10% loss | Around 12 | At 12 months |  |  |  |  |  |
|  | EG | CG | Responder | At least 5% loss | Around 12 | At 12 months |  |  |  |  |  |
| Hageman2017[21] | WE | WO | Absolute weight loss |  | Around 6 | At 6 months | kg | -0.80 | -2.50 | 0.9 | 0.87 |
|  | WD | WO | Absolute weight loss |  | Around 6 | At 6 months | kg | 0.90 | -0.80 | 2.7 | 0.89 |
|  | WE | WD | Absolute weight loss |  | Around 6 | At 6 months | kg | -1.70 | -3.40 | 0 | 0.87 |
|  | WE | WO | Anthrop | Waist circumference (cm) | Around 6 | At 6 months |  | 0.10 | -2.00 | 2.2 |  |
|  | WD | WO | Anthrop | Waist circumference (cm) | Around 6 | At 6 months |  | 1.60 | -0.50 | 3.7 |  |
|  | WE | WD | Anthrop | Waist circumference (cm) | Around 6 | At 6 months |  | -1.50 | -3.50 | 0.6 |  |
|  | WO | WD | Absolute weight loss |  | Longer than 12 | At 18 months | kg | -0.70 |  |  | 2.31 |
|  | WE | WD | Absolute weight loss |  | Longer than 12 | At 18 months | kg | -1.30 |  |  | 2.40 |
|  | WO | WE | Absolute weight loss |  | Longer than 12 | At 18 months | kg | 0.60 |  |  | 2.41 |
|  | WO | WD | Absolute weight loss |  | Longer than 12 | At 30 months | kg | -1.00 |  |  | 2.29 |
|  | WE | WD | Absolute weight loss |  | Longer than 12 | At 30 months | kg | -0.90 |  |  | 2.57 |
|  | WO | WE | Absolute weight loss |  | Longer than 12 | At 30 months | kg | -0.10 |  |  | 2.52 |
|  | WO | WD | Anthrop | Waist circumference (cm) | Longer than 12 | At 18 months |  | 0.30 |  |  | 1.90 |
|  | WE | WD | Anthrop | Waist circumference (cm) | Longer than 12 | At 18 months |  | -0.30 |  |  | 2.06 |
|  | WO | WE | Anthrop | Waist circumference (cm) | Longer than 12 | At 18 months |  | 0.60 |  |  | 2.03 |
| Hageman2017[21] | WO | WD | Anthrop | Waist circumference (cm) | Longer than 12 | At 30 months |  | 0.00 |  |  | 1.91 |
|  | WE | WD | Anthrop | Waist circumference (cm) | Longer than 12 | At 30 months |  | 0.50 |  |  | 2.08 |
|  | WO | WE | Anthrop | Waist circumference (cm) | Longer than 12 | At 30 months |  | -0.50 |  |  | 2.10 |
|  | WO | WD | Responder | At least 5% loss | Around 6 | At 6 months |  |  |  |  |  |
|  | WO | WE | Responder | At least 5% loss | Around 6 | At 6 months |  |  |  |  |  |
|  | WD | WE | Responder | At least 5% loss | Around 6 | At 6 months |  |  |  |  |  |
|  | WO | WD | Responder | At least 5% loss | Longer than 12 | At 18 months |  |  |  |  |  |
|  | WO | WE | Responder | At least 5% loss | Longer than 12 | At 18 months |  |  |  |  |  |
|  | WD | WE | Responder | At least 5% loss | Longer than 12 | At 18 months |  |  |  |  |  |
|  | WO | WD | Responder | At least 5% loss | Longer than 12 | At 30 months |  |  |  |  |  |
|  | WO | WE | Responder | At least 5% loss | Longer than 12 | At 30 months |  |  |  |  |  |
|  | WD | WE | Responder | At least 5% loss | Longer than 12 | At 30 months |  |  |  |  |  |
|  | WO | WD | Responder | At least 10% loss | Around 6 | At 6 months |  |  |  |  |  |
|  | WO | WE | Responder | At least 10% loss | Around 6 | At 6 months |  |  |  |  |  |
|  | WD | WE | Responder | At least 10% loss | Around 6 | At 6 months |  |  |  |  |  |
|  | WO | WD | Responder | At least 10% loss | Longer than 12 | At 18 months |  |  |  |  |  |
|  | WO | WE | Responder | At least 10% loss | Longer than 12 | At 18 months |  |  |  |  |  |
|  | WD | WE | Responder | At least 10% loss | Longer than 12 | At 18 months |  |  |  |  |  |
|  | WO | WD | Responder | At least 10% loss | Longer than 12 | At 30 months |  |  |  |  |  |
|  | WO | WE | Responder | At least 10% loss | Longer than 12 | At 30 months |  |  |  |  |  |
|  | WD | WE | Responder | At least 10% loss | Longer than 12 | At 30 months |  |  |  |  |  |
| Hesseldal2022,[22] Christensen2022[23] | DL | TAU | Absolute weight loss |  | Around 6 | At 6 months | kg | -4.20 | -5.50 | -2.8 | 0.69 |
|  | DL | TAU | Pct loss |  | Around 6 | At 6 months |  | -3.90 | -5.30 | -2.6 | 0.69 |
|  | DL | TAU | Responder | At least 5% loss | Around 6 | At 6 months |  |  |  |  |  |
|  | DL | TAU | BMI |  | Around 6 | At 6 months |  | -1.40 | -1.80 | -0.9 | 0.23 |
|  | DL | TAU | Anthrop | Hip circumference (cm) | Around 6 | At 6 months |  | -3.60 | -5.20 | -2 |  |
|  | DL | TAU | Anthrop | Waist circumference (cm) | Around 6 | At 6 months |  | -5.60 | -7.60 | -3.6 |  |
|  | DL | TAU | Anthrop | Waist to hip ratio | Around 6 | At 6 months |  | -0.02 | -0.04 | 0 |  |
|  | DL | TAU | Absolute weight loss |  | Around 12 | At 12 months (plus C19 extension) | kg | -3.00 | -4.80 | -1.3 | 0.89 |
|  | DL | TAU | Pct loss |  | Around 12 | At 12 months (plus C19 extension) |  | -3.20 | -5.00 | -1.4 | 0.92 |
|  | DL | TAU | Responder | At least 5% loss | Around 12 | At 12 months (plus C19 extension) |  |  |  |  |  |
|  | DL | TAU | BMI |  | Around 12 | At 12 months (plus C19 extension) |  | -1.00 | -1.70 | -0.4 | 0.33 |
|  | DL | TAU | Anthrop | Hip circumference (cm) | Around 12 | At 12 months (plus C19 extension) |  | -3.50 | -5.30 | -1.7 |  |
|  | DL | TAU | Anthrop | Waist circumference (cm) | Around 12 | At 12 months (plus C19 extension) |  | -5.30 | -7.80 | -2.8 |  |
|  | DL | TAU | Anthrop | Waist to hip ratio | Around 12 | At 12 months (plus C19 extension) |  | -0.02 | -0.36 | -0.003 |  |
|  | DL | TAU | Absolute weight loss |  | Longer than 12 | At 24 months | kg | -1.90 |  |  | 1.16 |
| Hutchesson2018[24] | BPBH | WL | Absolute weight loss |  | Around 6 | At 6 months | kg | -1.94 | -4.31 | 0.42 | 1.21 |
|  | BPBH | WL | BMI |  | Around 6 | At 6 months |  | -0.68 | -1.47 | 1.09 | 0.65 |
|  | BPBH | WL | Anthrop | Body fat (kg) | Around 6 | At 6 months |  | -3.10 | -5.69 | 0.52 |  |
|  | BPBH | WL | Anthrop | Body fat (%) | Around 6 | At 6 months |  | -2.00 | -4.33 | 0.33 |  |
|  | BPBH | WL | Anthrop | Waist circumference (cm) | Around 6 | At 6 months |  | -1.40 | -3.80 | 1 |  |
| Jane2017,[25]2018[26] | FG | CG | Pct loss |  | Around 6 | 24 weeks |  | -3.30 |  |  | 1.28 |
|  | PG | CG | Pct loss |  | Around 6 | 24 weeks |  | -2.10 |  |  | 1.07 |
|  | FG | PG | Pct loss |  | Around 6 | 24 weeks |  | -1.20 |  |  | 1.19 |
|  | FG | CG | BMI |  | Around 6 | 24 weeks |  | -1.00 |  |  | 0.46 |
|  | PG | CG | BMI |  | Around 6 | 24 weeks |  | -0.80 |  |  | 0.36 |
|  | FG | PG | BMI |  | Around 6 | 24 weeks |  | -0.20 |  |  | 0.50 |
|  | FG | CG | Anthrop | Waist circumference (cm) | Around 6 | 24 weeks |  | -2.70 |  |  | 1.36 |
|  | PG | CG | Anthrop | Waist circumference (cm) | Around 6 | 24 weeks |  | -1.20 |  |  | 1.20 |
|  | FG | PG | Anthrop | Waist circumference (cm) | Around 6 | 24 weeks |  | -1.50 |  |  | 1.36 |
|  | FG | CG | Anthrop | Hip circumference (cm) | Around 6 | 24 weeks |  | -1.80 |  |  | 1.11 |
|  | PG | CG | Anthrop | Hip circumference (cm) | Around 6 | 24 weeks |  | -1.70 |  |  | 0.85 |
|  | FG | PG | Anthrop | Hip circumference (cm) | Around 6 | 24 weeks |  | -0.10 |  |  | 1.11 |
|  | FG | CG | Anthrop | Fat mass (%) | Around 6 | 24 weeks |  | -2.00 |  |  | 0.79 |
|  | PG | CG | Anthrop | Fat mass (%) | Around 6 | 24 weeks |  | -0.80 |  |  | 0.59 |
|  | FG | PG | Anthrop | Fat mass (%) | Around 6 | 24 weeks |  | -1.20 |  |  | 0.79 |
|  | FG | CG | Anthrop | Lean mass (%) | Around 6 | 24 weeks |  | 0.90 |  |  | 0.37 |
|  | PG | CG | Anthrop | Lean mass (%) | Around 6 | 24 weeks |  | 0.40 |  |  | 0.28 |
|  | FG | PG | Anthrop | Lean mass (%) | Around 6 | 24 weeks |  | 0.50 |  |  | 0.37 |
|  | FG | CG | Absolute weight loss |  | Around 6 | 24 weeks | kg | -10.70 |  |  | 4.67 |
|  | PG | CG | Absolute weight loss |  | Around 6 | 24 weeks | kg | -9.50 |  |  | 4.73 |
|  | FG | PG | Absolute weight loss |  | Around 6 | 24 weeks | kg | -1.20 |  |  | 4.60 |
| Dunn2019[27] | Photo | Calorie | Absolute weight loss |  | Around 6 | At 6 months | kg | -0.10 |  |  | 0.30 |
| Falkenhain2021[28] | Ketogenic diet app with biofeedback | Calorie restricted low fat diet app | Absolute weight loss |  | Around 6 | 24 weeks | kg | -4.90 | -7.30 | -2.6 | 1.20 |
| Joseph2023[29] | Smart-walk | Smart-health | BMI |  | Around 6 | 8 months |  | -4.90 |  |  | 4.90 |
| Kempf2019[30] | TMC | C1 | Absolute weight loss |  | Around 6 | At 6 months | kg | -0.70 |  |  | 1.17 |
|  | C2 | C1 | Absolute weight loss |  | Around 6 | At 6 months | kg | 3.30 |  |  | 1.16 |
|  | TMC | C2 | Absolute weight loss |  | Around 6 | At 6 months | kg | -4.00 |  |  | 1.17 |
|  | TMC | C1 | Absolute weight loss |  | Around 12 | At 12 months | kg | -1.80 |  |  | 1.88 |
|  | C2 | C1 | Absolute weight loss |  | Around 12 | At 12 months | kg | 1.50 |  |  | 1.84 |
|  | TMC | C2 | Absolute weight loss |  | Around 12 | At 12 months | kg | -3.30 |  |  | 1.77 |
|  | TMC | C1 | Absolute weight loss |  | Longer than 12 | At 24 months | kg | -0.80 |  |  | 2.11 |
|  | C2 | C1 | Absolute weight loss |  | Longer than 12 | At 24 months | kg | 0.70 |  |  | 2.07 |
|  | TMC | C2 | Absolute weight loss |  | Longer than 12 | At 24 months | kg | -1.50 |  |  | 2.02 |
|  | TMC | C1 | Responder | At least 2% loss | Around 12 | At 12 months |  |  |  |  |  |
|  | C2 | C1 | Responder | At least 2% loss | Around 12 | At 12 months |  |  |  |  |  |
|  | TMC | C2 | Responder | At least 2% loss | Around 12 | At 12 months |  |  |  |  |  |
|  | TMC | C1 | Responder | At least 5% loss | Around 12 | At 12 months |  |  |  |  |  |
|  | C2 | C1 | Responder | At least 5% loss | Around 12 | At 12 months |  |  |  |  |  |
|  | TMC | C2 | Responder | At least 5% loss | Around 12 | At 12 months |  |  |  |  |  |
|  | TMC | C1 | Responder | At least 10% loss | Around 12 | At 12 months |  |  |  |  |  |
|  | C2 | C1 | Responder | At least 10% loss | Around 12 | At 12 months |  |  |  |  |  |
|  | TMC | C2 | Responder | At least 10% loss | Around 12 | At 12 months |  |  |  |  |  |
|  | TMC | C1 | BMI |  | Around 12 | At 12 months |  | -0.90 |  |  | 0.63 |
|  | C2 | C1 | BMI |  | Around 12 | At 12 months |  | 0.20 |  |  | 0.62 |
|  | TMC | C2 | BMI |  | Around 12 | At 12 months |  | -1.10 |  |  | 0.60 |
| Kempf2018[31] | TM/TMC | Control | Absolute weight loss |  | Around 12 | At 12 months | kg | -2.29 |  |  | 0.85 |
| Keshvarz2023[32] | Online circuit training | Online workout plan | Absolute weight loss |  | Around 6 | At 24 weeks | kg | 2.10 |  |  | 4.57 |
|  | Online circuit training | Online workout plan | Absolute weight loss |  | Around 12 | At 46 weeks | kg | 3.10 |  |  | 4.69 |
|  | Online circuit training | Online workout plan | BMI |  | Around 6 | At 24 weeks |  | 1.00 |  |  | 1.10 |
|  | Online circuit training | Online workout plan | BMI |  | Around 12 | At 46 weeks |  | 1.80 |  |  | 1.19 |
|  | Online circuit training | Online workout plan | Anthrop | Waist circumference (cm) | Around 6 | At 24 weeks |  | 1.30 |  |  | 3.86 |
|  | Online circuit training | Online workout plan | Anthrop | Waist circumference (cm) | Around 12 | At 46 weeks |  | 3.70 |  |  | 4.09 |
|  | Online circuit training | Online workout plan | Anthrop | Body fat (%) | Around 6 | At 24 weeks |  | 3.00 |  |  | 2.22 |
|  | Online circuit training | Online workout plan | Anthrop | Body fat (%) | Around 12 | At 46 weeks |  | 3.20 |  |  | 2.13 |
|  | Online circuit training | Online workout plan | Anthrop | Muscle mass (kg) | Around 6 | At 24 weeks |  | -0.90 |  |  | 2.24 |
|  | Online circuit training | Online workout plan | Anthrop | Muscle mass (kg) | Around 12 | At 46 weeks |  | -1.30 |  |  | 2.22 |
| Kharmats2022[33] | SMS | Printed messages | Absolute weight loss |  | Around 6 | At 42 weeks | kg | -0.46 | -1.85 | -0.924 | 0.24 |
|  | SMS | Printed messages | BMI |  | Around 6 | At 42 weeks |  | -0.25 | -0.80 | 0.252 | 0.27 |
|  | SMS | Printed messages | Pct loss |  | Around 6 | At 42 weeks |  | -0.63 | -2.06 | 0.84 | 0.74 |
| Kim2020[34] | digital CBT | self-report | Absolute weight loss |  | Around 6 | At 24 weeks | kg | 1.50 |  |  | 2.58 |
|  | digital CBT | self-report | BMI |  | Around 6 | At 24 weeks |  | 0.10 |  |  | 0.88 |
|  | digital CBT | self-report | Anthrop | Fat mass (%) | Around 6 | At 24 weeks |  | -0.60 |  |  | 1.93 |
|  | digital CBT | self-report | Anthrop | Lean body mass (%) | Around 6 | At 24 weeks |  | 0.70 |  |  | 0.74 |
|  | digital CBT | self-report | Pct loss |  | Around 6 | At 24 weeks |  | -1.00 |  |  | 2.02 |
|  | digital CBT | self-report | Pct loss | BMI | Around 6 | At 24 weeks |  | -1.19 |  |  | 2.25 |
|  | digital CBT | self-report | Anthrop | Fat mass change (%) | Around 6 | At 24 weeks |  | -6.10 |  |  | 4.76 |
|  | digital CBT | self-report | Anthrop | Lean body mass change (%) | Around 6 | At 24 weeks |  | 2.10 |  |  | 3.26 |
|  | digital CBT | self-report | Responder | At least 3% loss | Around 6 | At 24 weeks |  |  |  |  |  |
|  | digital CBT | self-report | Responder | At least 5% loss | Around 6 | At 24 weeks |  |  |  |  |  |
| Kohl2023[35] | interactive web-based program | non-interactive web-based program | Absolute weight loss |  | Around 6 | At 6 months | kg | -2.37 |  |  | 0.94 |
|  | interactive web-based program | non-interactive web-based program | Absolute weight loss |  | Around 12 | At 12 months | kg | -2.41 |  |  | 0.84 |
|  | interactive web-based program | non-interactive web-based program | Anthrop | Fat mass (kg) | Around 6 | At 6 months |  | -1.43 |  |  | 0.70 |
|  | interactive web-based program | non-interactive web-based program | Anthrop | Fat mass (kg) | Around 12 | At 12 months |  | -1.64 |  |  | 0.70 |
|  | interactive web-based program | non-interactive web-based program | Anthrop | Fat-free mass (kg) | Around 6 | At 6 months |  | -0.03 |  |  | 0.43 |
|  | interactive web-based program | non-interactive web-based program | Anthrop | Fat-free mass (kg) | Around 12 | At 12 months |  | -0.69 |  |  | 0.39 |
|  | interactive web-based program | non-interactive web-based program | Anthrop | Waist circumference (cm) | Around 6 | At 6 months |  | -1.63 |  |  | 0.87 |
|  | interactive web-based program | non-interactive web-based program | Anthrop | Waist circumference (cm) | Around 12 | At 12 months |  | -2.75 |  |  | 0.87 |
|  | interactive web-based program | non-interactive web-based program | Responder | At least 5% loss | Around 12 | At 12 months |  |  |  |  |  |
| Kurtzman2018[36] | Gamification | Control | Absolute weight loss |  | Around 6 | At 24 weeks | lbs | -2.00 |  |  | 1.99 |
| Laing2014[37] | Smartphone app | Usual care | Absolute weight loss |  | Around 6 | At 6 months | kg | -0.30 | -1.50 | 0.95 | 0.63 |
|  | Smartphone app | Usual care | Responder | At least 2.7 kg lost | Around 6 | At 6 months |  |  |  |  |  |
| Bennett2013,[38] Lanpher2016[39] | Shape Plan | Usual care | Absolute weight loss |  | Around 6 | At 6 months | kg | -1.10 | -2.30 | 0.04 | 0.60 |
|  | Shape Plan | Usual care | Absolute weight loss |  | Around 12 | At 12 months | kg | -1.40 | -2.80 | -0.1 | 0.69 |
|  | Shape Plan | Usual care | Absolute weight loss |  | Longer than 12 | At 18 months | kg | -1.70 | -3.30 | -0.2 | 0.79 |
|  | Shape Plan | Usual care | BMI |  | Around 6 | At 6 months |  | -0.40 | -0.80 | 0.03 | 0.21 |
|  | Shape Plan | Usual care | BMI |  | Around 12 | At 12 months |  | -0.60 | -1.10 | -0.1 | 0.26 |
|  | Shape Plan | Usual care | BMI |  | Longer than 12 | At 18 months |  | -0.60 | -1.20 | -0.1 | 0.28 |
|  | Shape Plan | Usual care | Anthrop | Waist circumference (cm) | Around 6 | At 6 months |  | -0.60 | -2.40 | 1.2 | 0.92 |
|  | Shape Plan | Usual care | Anthrop | Waist circumference (cm) | Around 12 | At 12 months |  | -1.30 | -3.10 | 0.5 | 0.92 |
|  | Shape Plan | Usual care | Anthrop | Waist circumference (cm) | Longer than 12 | At 18 months |  | -1.20 | -3.40 | 1 | 1.12 |
| LaRose2022[40] | aBWL + BE | aBWL | Absolute weight loss |  | Around 6 | At 6 months | kg | -0.25 | -1.79 | 1.29 | 0.79 |
|  | aBWL + SDT | aBWL | Absolute weight loss |  | Around 6 | At 6 months | kg | -0.18 | -1.67 | 1.31 | 0.76 |
|  | aBWL + SDT | aBWL + BE | Absolute weight loss |  | Around 6 | At 6 months | kg | 0.07 | -1.45 | 1.59 | 0.78 |
|  | aBWL + BE | aBWL | Pct loss |  | Around 6 | At 6 months |  | -0.37 | -2.03 | 1.29 | 0.85 |
|  | aBWL + SDT | aBWL | Pct loss |  | Around 6 | At 6 months |  | -0.25 | -1.86 | 1.37 | 0.82 |
|  | aBWL + SDT | aBWL + BE | Pct loss |  | Around 6 | At 6 months |  | 0.12 | -1.53 | 1.77 | 0.84 |
|  | aBWL + BE | aBWL | BMI |  | Around 6 | At 6 months |  | -0.16 | -0.70 | 0.38 | 0.28 |
|  | aBWL + SDT | aBWL | BMI |  | Around 6 | At 6 months |  | -0.17 | -0.69 | 0.36 | 0.27 |
|  | aBWL + SDT | aBWL + BE | BMI |  | Around 6 | At 6 months |  | -0.01 | -0.54 | 0.53 | 0.27 |
|  | aBWL + BE | aBWL | Anthrop | Waist circumference (cm) | Around 6 | At 6 months | kg | -0.14 | -1.76 | 1.48 | 0.83 |
|  | aBWL + SDT | aBWL | Anthrop | Waist circumference (cm) | Around 6 | At 6 months | kg | -0.33 | -1.94 | 1.29 | 0.82 |
|  | aBWL + SDT | aBWL + BE | Anthrop | Waist circumference (cm) | Around 6 | At 6 months | kg | -0.18 | -1.77 | 1.4 | 0.81 |
|  | aBWL + BE | aBWL | Anthrop | Body fat (%) | Around 6 | At 6 months | kg | -0.32 | -1.13 | 0.48 | 0.41 |
|  | aBWL + SDT | aBWL | Anthrop | Body fat (%) | Around 6 | At 6 months | kg | -0.37 | -1.17 | 0.42 | 0.41 |
|  | aBWL + SDT | aBWL + BE | Anthrop | Body fat (%) | Around 6 | At 6 months | kg | -0.05 | -0.81 | 0.72 | 0.39 |
|  | aBWL + BE | aBWL | Responder | At least 5% loss | Around 6 | At 6 months | kg |  |  |  |  |
|  | aBWL + SDT | aBWL | Responder | At least 5% loss | Around 6 | At 6 months | kg |  |  |  |  |
|  | aBWL + SDT | aBWL + BE | Responder | At least 5% loss | Around 6 | At 6 months | kg |  |  |  |  |
| Leahey2015[41] | SII | SI | Pct loss |  | Around 12 | At 12 months |  | -1.90 |  |  | 0.97 |
|  | SII | SI | Responder | At least 5% loss | Around 12 | At 12 months |  |  |  |  |  |
| Leahey2016[42] | CB pro | Standard | Absolute weight loss |  | Around 12 | At 10 months | kg | -5.30 |  |  | 2.11 |
|  | CB peer | Standard | Absolute weight loss |  | Around 12 | At 10 months | kg | -4.00 |  |  | 2.03 |
|  | CB pro | CB peer | Absolute weight loss |  | Around 12 | At 10 months | kg | -1.30 |  |  | 1.80 |
|  | CB pro | Standard | Responder | Maintained at least 5% weight loss | Around 12 | At 10 months |  |  |  |  |  |
|  | CB peer | Standard | Responder | Maintained at least 5% weight loss | Around 12 | At 10 months |  |  |  |  |  |
|  | CB pro | CB peer | Responder | Maintained at least 5% weight loss | Around 12 | At 10 months |  |  |  |  |  |
|  | CB pro | Standard | Pct loss |  | Around 12 | At 10 months |  | -5.60 |  |  | 1.97 |
|  | CB peer | Standard | Pct loss |  | Around 12 | At 10 months |  | -4.30 |  |  | 1.74 |
|  | CB pro | CB peer | Pct loss |  | Around 12 | At 10 months |  | -1.30 |  |  | 1.97 |
| Little2016,[43]2017[44] | POWER + remote | Control | Absolute weight loss |  | Around 6 | At 6 months | kg | -1.97 | -3.18 | -0.76 | 0.62 |
|  | POWER + remote | Control | Absolute weight loss |  | Around 12 | At 12 months | kg | -0.58 | -1.88 | 0.72 | 0.66 |
|  | POWER + remote | Control | Responder | At least 5% loss | Around 6 | At 6 months |  |  |  |  |  |
|  | POWER + remote | Control | Responder | At least 5% loss | Around 12 | At 12 months |  |  |  |  |  |
|  | POWER + remote | Control | Anthrop | Body fat (%) | Around 12 | At 12 months |  | -0.53 | -1.47 | 0.42 |  |
| Markkanen2024[45] | mHBCSS | WL | Pct loss |  | Around 6 | At 6 months |  | -2.70 | -3.80 | -1.6 | 0.56 |
|  | mHBCSS | WL | BMI |  | Around 6 | At 6 months |  | -0.90 | -1.30 | -0.6 | 0.18 |
|  | mHBCSS | WL | Anthrop | Waist circumference (cm) | Around 6 | At 6 months |  | -2.30 | -3.20 | -1.4 |  |
|  | mHBCSS | WL | Responder | At least 5% loss | Around 6 | At 6 months |  |  |  |  |  |
|  | mHBCSS | WL | Responder | At least 10% loss | Around 6 | At 6 months |  |  |  |  |  |
| McConnon2007[46] | Internet group | Usual care | BMI |  | Around 12 | At 12 months |  | 0.30 | -0.50 | 1 | 0.38 |
|  | Internet group | Usual care | Absolute weight loss |  | Around 12 | At 12 months | kg | 0.50 | -0.80 | 1.8 | 0.66 |
|  | Internet group | Usual care | Responder | At least 5% loss | Around 12 | At 12 months |  |  |  |  |  |
| Morgan2009,[47]2011[48] | Internet | Information and self-help | Absolute weight loss |  | Around 6 | At 6 months | kg | -1.90 | -4.80 | 1 | 1.48 |
|  | Internet | Information and self-help | Anthrop | Waist circumference (cm) | Around 6 | At 6 months |  | -1.40 | -4.40 | 1.6 |  |
|  | Internet | Information and self-help | BMI |  | Around 6 | At 6 months |  | -0.50 | -1.40 | 0.4 | 0.46 |
|  | Internet | Information and self-help | Pct loss |  | Around 6 | At 6 months |  | -1.80 |  |  | 1.49 |
|  | Internet | Information and self-help | Responder | At least 5% loss | Around 6 | At 6 months |  |  |  |  |  |
|  | Internet | Information and self-help | Absolute weight loss |  | Around 12 | At 12 months | kg | -2.20 | -5.50 | 1.05 | 1.67 |
|  | Internet | Information and self-help | Anthrop | Waist circumference (cm) | Around 12 | At 12 months |  | -1.90 | -5.00 | 1.1 |  |
|  | Internet | Information and self-help | BMI |  | Around 12 | At 12 months |  | -0.70 | -1.70 | 0.3 | 0.51 |
|  | Internet | Information and self-help | Pct loss |  | Around 12 | At 12 months |  | -2.70 |  |  | 3.26 |
|  | Internet | Information and self-help | Responder | At least 5% loss | Around 12 | At 12 months |  |  |  |  |  |
| Morgan2013,[49] Blomfield2014[50] | Resource group | WL | Absolute weight loss |  | Around 6 | At 6 months | kg | -3.20 | -4.90 | -1.5 | 0.87 |
|  | Online | WL | Absolute weight loss |  | Around 6 | At 6 months | kg | -4.20 | -5.90 | -2.5 | 0.87 |
|  | Online | Resource | Absolute weight loss |  | Around 6 | At 6 months | kg | -1.00 | -2.60 | 0.7 | 0.84 |
|  | Resource group | WL | Anthrop | Waist circumference umbilicus (cm) | Around 6 | At 6 months |  | -2.80 | -4.40 | -1.2 |  |
|  | Online | WL | Anthrop | Waist circumference umbilicus (cm) | Around 6 | At 6 months |  | -4.50 | -6.10 | -2.9 |  |
|  | Online | Resource | Anthrop | Waist circumference umbilicus (cm) | Around 6 | At 6 months |  | -1.70 | -3.30 | -0.1 |  |
|  | Resource group | WL | BMI |  | Around 6 | At 6 months |  | -1.00 | -1.60 | -0.5 | 0.28 |
|  | Online | WL | BMI |  | Around 6 | At 6 months |  | -1.30 | -1.90 | -0.8 | 0.28 |
|  | Online | Resource | BMI |  | Around 6 | At 6 months |  | -0.30 | -0.80 | 0.2 | 0.26 |
|  | Resource group | WL | Pct loss |  | Around 6 | At 6 months |  | -3.30 | -5.00 | -1.6 | 0.87 |
|  | Online | WL | Pct loss |  | Around 6 | At 6 months |  | -4.30 | -5.90 | -2.6 | 0.84 |
|  | Online | Resource | Pct loss |  | Around 6 | At 6 months |  | -1.00 | -2.60 | 0.7 | 0.84 |
|  | Resource group | WL | Anthrop | Waist circumference largest (cm) | Around 6 | At 6 months |  | -2.90 | -4.60 | -1.1 |  |
|  | Online | WL | Anthrop | Waist circumference largest (cm) | Around 6 | At 6 months |  | -4.90 | -6.70 | -3.1 |  |
| Morgan2013,[49] Blomfield2014[50] | Online | Resource | Anthrop | Waist circumference largest (cm) | Around 6 | At 6 months |  | -2.00 | -3.80 | -0.3 |  |
|  | Resource group | WL | Anthrop | Fat mass (%) | Around 6 | At 6 months |  | -1.70 | -2.80 | -0.5 |  |
|  | Online | WL | Anthrop | Fat mass (%) | Around 6 | At 6 months |  | -2.10 | -3.30 | -0.9 |  |
|  | Online | Resource | Anthrop | Fat mass (%) | Around 6 | At 6 months |  | -0.40 | -1.60 | 0.7 |  |
|  | Resource group | WL | Anthrop | Visceral fat area (cm2) | Around 6 | At 6 months |  | -6.90 | -12.60 | -1.2 |  |
|  | Online | WL | Anthrop | Visceral fat mass (%) | Around 6 | At 6 months |  | -10.80 | -16.50 | -5.1 |  |
|  | Online | Resource | Anthrop | Visceral fat mass (%) | Around 6 | At 6 months |  | -3.90 | -9.60 | 1.8 |  |
|  | Resource group | WL | Anthrop | Skeletal muscle mass (kg) | Around 6 | At 6 months |  | -0.20 | -0.70 | 0.2 |  |
|  | Online | WL | Anthrop | Skeletal muscle mass (kg) | Around 6 | At 6 months |  | -0.50 | -1.00 | 0.1 |  |
|  | Online | Resource | Anthrop | Skeletal muscle mass (kg) | Around 6 | At 6 months |  | -0.30 | -0.80 | 0.1 |  |
|  | Online | Resource | Responder | At least 5% loss | Around 6 | At 6 months |  |  |  |  |  |
| Mueller2022,[51]2023[52] | SWiM-C | UC | Absolute weight loss |  | Around 12 | At 12 months | kg | -0.81 | -2.24 | 0.61 | 0.73 |
| Patel2019[53] | Sim | App | Absolute weight loss |  | Around 6 | At 6 months | kg | -1.17 |  |  | 1.11 |
|  | Seq | App | Absolute weight loss |  | Around 6 | At 6 months | kg | -0.38 | -2.46 | 1.71 | 1.06 |
|  | Sim | Seq | Absolute weight loss |  | Around 6 | At 6 months | kg | -0.80 |  |  | 1.06 |
|  | Sim | App | BMI |  | Around 6 | At 6 months |  | -0.39 |  |  | 0.39 |
|  | Seq | App | BMI |  | Around 6 | At 6 months |  | -0.15 | -0.88 | 0.59 | 0.38 |
|  | Sim | Seq | BMI |  | Around 6 | At 6 months |  | -0.25 |  |  | 0.37 |
| Patrick2011[54] | Web-based intervention | Control | Absolute weight loss |  | Around 12 | At 12 months | kg | -0.69 | -1.52 | 0.135 | 0.42 |
|  | Web-based intervention | Control | BMI |  | Around 12 | At 12 months |  | -0.27 | -0.54 | 0.003 | 0.14 |
|  | Web-based intervention | Control | Anthrop | Waist circumference (cm) | Around 12 | At 12 months |  | -0.29 | -1.16 | 0.585 |  |
| Rogers2016[55] | EN-TECH | TECH | Absolute weight loss |  | Around 6 | At 6 months | kg | -1.20 |  |  | 5.25 |
|  | EN-TECH | TECH | BMI |  | Around 6 | At 6 months |  | -0.10 |  |  | 1.56 |
|  | EN-TECH | TECH | Anthrop | Waist circumference (cm) | Around 6 | At 6 months |  | -2.40 |  |  | 4.60 |
|  | EN-TECH | TECH | Anthrop | Fat mass (kg) | Around 6 | At 6 months |  | -1.60 |  |  | 3.61 |
|  | EN-TECH | TECH | Anthrop | Lean body mass (kg) | Around 6 | At 6 months |  | 0.00 |  |  | 3.35 |
|  | EN-TECH | TECH | Anthrop | Percent body fat (%) | Around 6 | At 6 months |  | -1.10 |  |  | 2.27 |
| Ross2016[56] | TECH | ST | Absolute weight loss |  | Around 6 | At 6 months | kg | -2.76 |  |  | 1.82 |
|  | TECH | ST | Pct loss |  | Around 6 | At 6 months |  | -3.13 |  |  | 1.85 |
|  | TECH | ST | Responder | At least 5% loss | Around 6 | At 6 months |  |  |  |  |  |
|  | TECH | ST | Responder | At least 3% loss | Around 6 | At 6 months |  |  |  |  |  |
|  | TECH | ST | Responder | At least 10% loss | Around 6 | At 6 months |  |  |  |  |  |
| Roth2023[57] | Zanadio | WL | Pct loss |  | Around 6 | At 6 months |  | -4.25 |  |  | 0.95 |
|  | Zanadio | WL | Pct loss |  | Around 12 | At 12 months |  | -7.75 | -9.61 | -5.89 | 0.95 |
|  | Zanadio | WL | Anthrop | Waist to hip ratio | Around 12 | At 12 months |  | -0.03 | -0.04 | -0.01 |  |
|  | Zanadio | WL | Responder | At least 5% loss | Around 12 | At 12 months |  |  |  |  |  |
|  | Zanadio | WL | Responder | At least 10% loss | Around 12 | At 12 months |  |  |  |  |  |
| Shapiro2012[58] | Text4Diet | Control | Absolute weight loss |  | Around 6 | At 6 months | lbs | -0.50 |  |  | 0.31 |
|  | Text4Diet | Control | Absolute weight loss |  | Around 12 | At 12 months | lbs | -0.30 |  |  | 0.44 |
|  | Text4Diet | Control | Pct loss |  | Around 12 | At 12 months |  | 1.00 |  |  | 1.17 |
| Shuger2011[59] | SenseWear Alone | Standard care | Absolute weight loss |  | Around 6 | At 9 months | kg | -3.72 |  |  | 4.31 |
|  | SenseWear Alone | Standard care | BMI |  | Around 6 | At 9 months |  | -0.60 |  |  | 1.33 |
|  | SenseWear Alone | Standard care | Anthrop | Waist circumference (%) | Around 6 | At 9 months |  | -0.45 |  |  | 3.22 |
|  | SenseWear Alone | Standard care | Anthrop | Body fat (%) | Around 6 | At 9 months |  | -0.74 |  |  | 1.26 |
| Silina2017[60] | Intervention | Control | Absolute weight loss |  | Around 12 | At 12 months | kg | -3.18 | -5.30 | -1.04 | 1.08 |
|  | Intervention | Control | BMI |  | Around 12 | At 12 months |  | -1.13 | -1.86 | -0.4 | 0.37 |
|  | Intervention | Control | Anthrop | Waist circumference (cm) | Around 12 | At 12 months |  | -4.56 | -6.80 | -2.3 | 1.13 |
|  | Intervention | Control | Anthrop | Hip circumference (cm) | Around 12 | At 12 months |  | -4.00 | -5.90 | -2 | 0.99 |
|  | Intervention | Control | Anthrop | Waist-hip ratio | Around 12 | At 12 months |  | -0.01 | -0.26 | 0.006 |  |
|  | Intervention | Control | Responder | At least 10% loss | Around 12 | At 12 months |  |  |  |  |  |
|  | Intervention | Control | Responder | At least 5% loss | Around 12 | At 12 months |  |  |  |  |  |
| Simpson2020,[61]2020[62] | HelpMeDoIt! | Control | BMI |  | Around 12 | At 12 months |  | -0.20 | -1.40 | 1.1 | 0.64 |
|  | HelpMeDoIt! | Control | Absolute weight loss |  | Around 12 | At 12 months | kg | -0.08 | -0.53 | 0.37 | 0.23 |
|  | HelpMeDoIt! | Control | Anthrop | Waist circumference (cm) | Around 12 | At 12 months |  | 0.24 | -0.22 | 0.7 |  |
|  | HelpMeDoIt! | Control | Anthrop | Waist to hip ratio | Around 12 | At 12 months |  | 0.30 | -0.12 | 0.73 |  |
|  | HelpMeDoIt! | Control | Responder | At least 5 kg loss | Around 12 | At 12 months |  |  |  |  |  |
|  | HelpMeDoIt! | Control | Responder | At least 10 kg loss | Around 12 | At 12 months |  |  |  |  |  |
| Sniehotta2019[63] | NULevel | Control | Absolute weight loss |  | Around 12 | At 12 months | kg | -0.07 | -1.90 | 1.7 | 0.92 |
| Steinberg2013[64] | Shape Plan | Education control | Absolute weight loss |  | Around 6 | At 6 months | kg | -2.41 | -5.22 | 0.39 | 1.43 |
|  | Shape Plan | Education control | Pct loss |  | Around 6 | At 6 months |  | -2.29 | -4.70 | 0.12 | 1.23 |
|  | Shape Plan | Education control | BMI |  | Around 6 | At 6 months |  | -0.89 | -1.93 | 0.15 | 0.53 |
| Tate2001[65] | Behaviour therapy | Education | Absolute weight loss |  | Around 6 | At 6 months | kg | -1.60 |  |  | 0.78 |
|  | Behaviour therapy | Education | Responder | At least 5% loss | Around 6 | At 6 months |  |  |  |  |  |
|  | Behaviour therapy | Education | Anthrop | Waist circumference (cm) | Around 6 | At 6 months |  | -2.30 |  |  | 0.99 |
| Tate2006[66] | AF | NC | Absolute weight loss |  | Around 6 | At 6 months | kg | -1.50 |  |  | 1.07 |
|  | HC | NC | Absolute weight loss |  | Around 6 | At 6 months | kg | -3.90 |  |  | 1.46 |
|  | AF | HC | Absolute weight loss |  | Around 6 | At 6 months | kg | 2.40 |  |  | 1.67 |
|  | AF | NC | Pct loss |  | Around 6 | At 6 months |  | -2.50 |  |  | 1.20 |
|  | HC | NC | Pct loss |  | Around 6 | At 6 months |  | -5.30 |  |  | 1.11 |
|  | AF | HC | Pct loss |  | Around 6 | At 6 months |  | 2.80 |  |  | 1.21 |
|  | AF | NC | Responder | At least 5% loss | Around 6 | At 6 months |  |  |  |  |  |
|  | HC | NC | Responder | At least 5% loss | Around 6 | At 6 months |  |  |  |  |  |
|  | AF | HC | Responder | At least 5% loss | Around 6 | At 6 months |  |  |  |  |  |
| Tate2022[67] | IWL | EUC | Absolute weight loss |  | Around 6 | At 6 months | kg | -2.60 |  |  | 0.72 |
|  | IWL+PCP | EUC | Absolute weight loss |  | Around 6 | At 6 months | kg | -2.26 |  |  | 0.69 |
|  | IWL | IWL+PCP | Absolute weight loss |  | Around 6 | At 6 months | kg | -0.34 |  |  | 0.66 |
|  | IWL | EUC | Absolute weight loss |  | Around 12 | At 12 months | kg | -2.23 |  |  | 0.69 |
|  | IWL+PCP | EUC | Absolute weight loss |  | Around 12 | At 12 months | kg | -2.17 |  |  | 0.69 |
|  | IWL | IWL+PCP | Absolute weight loss |  | Around 12 | At 12 months | kg | -0.06 |  |  | 0.78 |
|  | IWL | EUC | Pct loss |  | Around 6 | At 6 months |  | -2.86 |  |  | 0.87 |
|  | IWL+PCP | EUC | Pct loss |  | Around 6 | At 6 months |  | -2.65 |  |  | 0.81 |
|  | IWL | IWL+PCP | Pct loss |  | Around 6 | At 6 months |  | -0.21 |  |  | 0.84 |
|  | IWL | EUC | Pct loss |  | Around 12 | At 12 months |  | -2.76 |  |  | 0.84 |
|  | IWL+PCP | EUC | Pct loss |  | Around 12 | At 12 months |  | -2.77 |  |  | 0.84 |
|  | IWL | IWL+PCP | Pct loss |  | Around 12 | At 12 months |  | 0.01 |  |  | 0.84 |
|  | IWL | EUC | BMI |  | Around 6 | At 6 months |  | -1.04 |  |  | 0.32 |
|  | IWL+PCP | EUC | BMI |  | Around 6 | At 6 months |  | -0.94 |  |  | 0.29 |
|  | IWL | IWL+PCP | BMI |  | Around 6 | At 6 months |  | -0.10 |  |  | 0.30 |
|  | IWL | EUC | BMI |  | Around 12 | At 12 months |  | -0.98 |  |  | 0.30 |
|  | IWL+PCP | EUC | BMI |  | Around 12 | At 12 months |  | -0.98 |  |  | 0.30 |
|  | IWL | IWL+PCP | BMI |  | Around 12 | At 12 months |  | 0.00 |  |  | 0.30 |
|  | IWL | EUC | Responder | At least 5% loss | Around 6 | At 6 months |  |  |  |  |  |
|  | IWL+PCP | EUC | Responder | At least 5% loss | Around 6 | At 6 months |  |  |  |  |  |
|  | IWL | IWL+PCP | Responder | At least 5% loss | Around 6 | At 6 months |  |  |  |  |  |
|  | IWL | EUC | Responder | At least 5% loss | Around 12 | At 12 months |  |  |  |  |  |
|  | IWL+PCP | EUC | Responder | At least 5% loss | Around 12 | At 12 months |  |  |  |  |  |
|  | IWL | IWL+PCP | Responder | At least 5% loss | Around 12 | At 12 months |  |  |  |  |  |
| Teeriniemi2018[68] | SHG + HBCSS | HBCSS | Absolute weight loss |  | Around 12 | At 12 months | kg | -0.20 |  |  | 0.61 |
|  | SHG + HBCSS | UC | Absolute weight loss |  | Around 12 | At 12 months | kg | -1.10 |  |  | 0.58 |
|  | HBCSS | UC | Absolute weight loss |  | Around 12 | At 12 months | kg | -0.90 |  |  | 0.54 |
|  | SHG + HBCSS | HBCSS | Absolute weight loss |  | Longer than 12 | At 24 months | kg | 0.60 |  |  | 0.76 |
|  | SHG + HBCSS | UC | Absolute weight loss |  | Longer than 12 | At 24 months | kg | -0.40 |  |  | 0.69 |
|  | HBCSS | UC | Absolute weight loss |  | Longer than 12 | At 24 months | kg | -1.00 |  |  | 0.69 |
|  | SHG + HBCSS | HBCSS | Pct loss |  | Around 12 | At 12 months |  | -0.80 |  |  | 0.88 |
|  | SHG + HBCSS | UC | Pct loss |  | Around 12 | At 12 months |  | -1.80 |  |  | 0.90 |
|  | HBCSS | UC | Pct loss |  | Around 12 | At 12 months |  | -1.00 |  |  | 0.80 |
|  | SHG + HBCSS | HBCSS | Pct loss |  | Longer than 12 | At 24 months |  | 0.50 |  |  | 1.06 |
|  | SHG + HBCSS | UC | Pct loss |  | Longer than 12 | At 24 months |  | -0.60 |  |  | 1.02 |
|  | HBCSS | UC | Pct loss |  | Longer than 12 | At 24 months |  | -1.10 |  |  | 1.01 |
|  | SHG + HBCSS | HBCSS | Responder | At least 10% loss | Longer than 12 | At 24 months |  |  |  |  |  |
|  | SHG + HBCSS | UC | Responder | At least 10% loss | Longer than 12 | At 24 months |  |  |  |  |  |
|  | HBCSS | UC | Responder | At least 10% loss | Longer than 12 | At 24 months |  |  |  |  |  |
|  | SHG + HBCSS | HBCSS | Responder | At least 5% loss | Longer than 12 | At 24 months |  |  |  |  |  |
|  | SHG + HBCSS | UC | Responder | At least 5% loss | Longer than 12 | At 24 months |  |  |  |  |  |
|  | HBCSS | UC | Responder | At least 5% loss | Longer than 12 | At 24 months |  |  |  |  |  |
|  | SHG + HBCSS | HBCSS | Anthrop | Waist circumference (cm) | Longer than 12 | At 24 months |  | 0.10 |  |  | 0.74 |
|  | SHG + HBCSS | UC | Anthrop | Waist circumference (cm) | Longer than 12 | At 24 months |  | -1.50 |  |  | 0.76 |
|  | HBCSS | UC | Anthrop | Waist circumference (cm) | Longer than 12 | At 24 months |  | -1.60 |  |  | 0.74 |
| Dombrowski2020,[69]2020[70] | SMS | WL | Absolute weight loss |  | Around 12 | At 12 months | kg | -0.47 |  |  | 1.29 |
|  | SMS + incentive | WL | Absolute weight loss |  | Around 12 | At 12 months | kg | -2.12 |  |  | 1.25 |
|  | SMS | SMS + incentive | Absolute weight loss |  | Around 12 | At 12 months | kg | 1.65 |  |  | 1.20 |
|  | SMS | WL | Pct loss |  | Around 12 | At 12 months |  | -0.42 |  |  | 1.08 |
|  | SMS + incentive | WL | Pct loss |  | Around 12 | At 12 months |  | -1.88 |  |  | 1.14 |
|  | SMS | SMS + incentive | Pct loss |  | Around 12 | At 12 months |  | 1.46 |  |  | 1.10 |
|  | SMS | WL | Responder | At least 3% loss | Around 12 | At 12 months |  |  |  |  |  |
|  | SMS + incentive | WL | Responder | At least 3% loss | Around 12 | At 12 months |  |  |  |  |  |
|  | SMS | SMS + incentive | Responder | At least 3% loss | Around 12 | At 12 months |  |  |  |  |  |
|  | SMS | WL | Responder | At least 5% loss | Around 12 | At 12 months |  |  |  |  |  |
|  | SMS + incentive | WL | Responder | At least 5% loss | Around 12 | At 12 months |  |  |  |  |  |
|  | SMS | SMS + incentive | Responder | At least 5% loss | Around 12 | At 12 months |  |  |  |  |  |
|  | SMS | WL | Responder | At least 10% loss | Around 12 | At 12 months |  |  |  |  |  |
|  | SMS + incentive | WL | Responder | At least 10% loss | Around 12 | At 12 months |  |  |  |  |  |
|  | SMS | SMS + incentive | Responder | At least 10% loss | Around 12 | At 12 months |  |  |  |  |  |
|  | SMS | WL | BMI |  | Around 12 | At 12 months |  | -0.01 |  |  | 0.42 |
|  | SMS + incentive | WL | BMI |  | Around 12 | At 12 months |  | -0.56 |  |  | 0.42 |
|  | SMS | SMS + incentive | BMI |  | Around 12 | At 12 months |  | 0.55 |  |  | 0.37 |
|  | SMS | WL | Anthrop | Waist circumference (cm) | Around 12 | At 12 months |  | -0.03 |  |  | 1.03 |
|  | SMS + incentive | WL | Anthrop | Waist circumference (cm) | Around 12 | At 12 months |  | -1.69 |  |  | 1.06 |
|  | SMS | SMS + incentive | Anthrop | Waist circumference (cm) | Around 12 | At 12 months |  | 1.66 |  |  | 1.02 |
|  | SMS | SMS + incentive | Absolute weight loss |  | Around 6 | At 6 months | kg | 1.17 |  |  | 1.13 |
|  | SMS | SMS + incentive | Pct loss |  | Around 6 | At 6 months |  | 1.05 |  |  | 1.05 |
| Dombrowski2020,[69]2020[70] | SMS | SMS + incentive | Responder | At least 3% loss | Around 6 | At 6 months |  |  |  |  |  |
|  | SMS | SMS + incentive | Responder | At least 5% loss | Around 6 | At 6 months |  |  |  |  |  |
|  | SMS | SMS + incentive | Responder | At least 10% loss | Around 6 | At 6 months |  |  |  |  |  |
|  | SMS | SMS + incentive | BMI |  | Around 6 | At 6 months |  | 0.24 |  |  | 0.35 |
|  | SMS | SMS + incentive | Anthrop | Waist circumference (cm) | Around 6 | At 6 months |  | 1.48 |  |  | 1.21 |
| Young2017[71] | SHED-IT | WL | Absolute weight loss |  | Around 6 | At 6 months | kg | -1.90 | -3.70 | -0.1 | 0.92 |
|  | SHED-IT | WL | Absolute weight loss |  | Around 12 | At 12 months | kg | -1.60 | -3.80 | 0.6 | 1.12 |
|  | SHED-IT | WL | Absolute weight loss |  | Longer than 12 | At 36 months | kg | 0.50 | -1.90 | 2.9 | 1.22 |
|  | SHED-IT | WL | BMI |  | Around 6 | At 6 months |  | -0.60 | -1.10 | 0 | 0.28 |
|  | SHED-IT | WL | BMI |  | Around 12 | At 12 months |  | -0.40 | -1.10 | 0.2 | 0.33 |
|  | SHED-IT | WL | BMI |  | Longer than 12 | At 36 months |  | 0.30 | -0.40 | 1.1 | 0.38 |
|  | SHED-IT | WL | Anthrop | Waist circumference (umbilicus) (cm) | Around 6 | At 6 months |  | -1.30 | -2.80 | 0.2 |  |
|  | SHED-IT | WL | Anthrop | Waist circumference (umbilicus) (cm) | Around 12 | At 12 months |  | -1.20 | -3.20 | 0.7 |  |
|  | SHED-IT | WL | Anthrop | Waist circumference (umbilicus) (cm) | Longer than 12 | At 36 months |  | 0.40 | -2.10 | 2.9 |  |
|  | SHED-IT | WL | Anthrop | Body fat (%) | Around 6 | At 6 months |  | -0.80 | -1.90 | 0.3 |  |
|  | SHED-IT | WL | Anthrop | Body fat (%) | Around 12 | At 12 months |  | -0.90 | -2.30 | 0.6 |  |
|  | SHED-IT | WL | Anthrop | Body fat (%) | Longer than 12 | At 36 months |  | -0.60 | -2.20 | 1 |  |
|  | SHED-IT | WL | Anthrop | Skeletal muscle mass (%) | Around 6 | At 6 months |  | 0.50 | -0.20 | 1.1 |  |
|  | SHED-IT | WL | Anthrop | Skeletal muscle mass (%) | Around 12 | At 12 months |  | 0.50 | -0.30 | 1.2 |  |
|  | SHED-IT | WL | Anthrop | Skeletal muscle mass (%) | Longer than 12 | At 36 months |  | 0.40 | -0.50 | 1.3 |  |
|  | SHED-IT | WL | Anthrop | Visceral fat area (cm2) | Around 6 | At 6 months |  | -6.10 | -11.90 | -0.4 |  |
|  | SHED-IT | WL | Anthrop | Visceral fat area (cm2) | Around 12 | At 12 months |  | -4.00 | -11.20 | 3.2 |  |
|  | SHED-IT | WL | Anthrop | Visceral fat area (cm2) | Longer than 12 | At 36 months |  | 4.60 | -7.00 | 16.3 |  |
|  | SHED-IT | WL | Responder | Maintained at least 5% weight loss | Longer than 12 | At 36 months |  |  |  |  |  |
| Womble2004[72] | E.diets | Weight loss manual | Pct loss |  | Around 12 | At 52 weeks |  | 2.90 |  |  | 1.41 |
|  | E.diets | Weight loss manual | Absolute weight loss |  | Around 12 | At 52 weeks | kg | 2.50 |  |  | 1.22 |
| Olson2016, [73]Wipfli2019[74] | SHIFT | Control | Absolute weight loss |  | Around 6 | At 6 months | lbs | -7.29 | -9.76 | -4.81 | 1.26 |
|  | SHIFT | Control | BMI |  | Around 6 | At 6 months |  | -1.00 | -1.39 | -0.62 | 0.20 |
|  | SHIFT | Control | Anthrop | Waist circumference (cm) | Around 6 | At 6 months |  | -0.76 | -1.25 | -0.27 |  |
|  | SHIFT | Control | Anthrop | Waist to hip ratio | Around 6 | At 6 months |  | -0.01 | -0.02 | 0 |  |
|  | SHIFT | Control | Anthrop | Body fat (%) | Around 6 | At 6 months |  | -2.09 | -2.99 | -1.19 |  |
| West2019[75] | Video-based chat with cellular-enabled scale | Text-based chat | Absolute weight loss |  | Around 6 | At 6 months | kg | -1.70 |  |  | 1.69 |
|  | Video-based chat with cellular-enabled scale | Text-based chat | Pct loss |  | Around 6 | At 6 months |  | -2.00 |  |  | 1.82 |
|  | Video-based chat with cellular-enabled scale | Text-based chat | Responder | At least 5% loss | Around 6 | At 6 months |  |  |  |  |  |
|  | Video-based chat with cellular-enabled scale | Text-based chat | Responder | At least 10% loss | Around 6 | At 6 months |  |  |  |  |  |
| West2016[76] | BT+MI | BT | Absolute weight loss |  | Around 6 | At 6 months | kg | 0.40 |  |  | 0.32 |
|  | BT+MI | BT | Absolute weight loss |  | Longer than 12 | At 18 months | kg | -0.20 |  |  | 1.36 |
|  | BT+MI | BT | Responder | At least 5% loss | Around 6 | At 6 months |  |  |  |  |  |
|  | BT+MI | BT | Responder | At least 5% loss | Longer than 12 | At 18 months |  |  |  |  |  |
|  | BT+MI | BT | Responder | At least 10% loss | Around 6 | At 6 months |  |  |  |  |  |
|  | BT+MI | BT | Responder | At least 10% loss | Longer than 12 | At 18 months |  |  |  |  |  |
| van Wier2009,[77]2011,[78]2012[79] | Internet group | Control | Absolute weight loss |  | Around 6 | At 6 months | kg | -0.60 | -1.30 | -0.01 | 0.33 |
|  | Internet group | Control | Responder | At least 5% loss | Around 6 | At 6 months |  |  |  |  |  |
|  | Internet group | Control | Anthrop | Waist circumference (cm) | Around 6 | At 6 months |  | -1.20 | -2.10 | -0.4 |  |
|  | Internet group | Control | Absolute weight loss |  | Longer than 12 | At 24 months | kg | -0.90 | -2.00 | 0.3 | 0.59 |
|  | Internet group | Control | Anthrop | Waist circumference (cm) | Longer than 12 | At 24 months |  | -0.30 | -1.30 | 0.8 |  |
|  | Internet group | Control | Responder | At least 5% loss | Longer than 12 | At 24 months |  |  |  |  |  |
| van Genugten2012[80] | Tailored | Generic | BMI |  | Around 6 | At 8 months post-baseline (6 months post-intervention) |  | 1.24 |  |  | 0.73 |
|  | Tailored | Generic | Anthrop | Waist circumference (cm) | Around 6 | At 8 months post-baseline (6 months post-intervention) |  | 0.93 |  |  | 0.60 |
|  | Tailored | Generic | Anthrop | Skin fold thickness (cm) | Around 6 | At 8 months post-baseline (6 months post-intervention) |  | -0.21 |  |  | 3.35 |
| Turner-McGrievy2017[81] | Bite | App | Absolute weight loss |  | Around 6 | At 6 months | kg | 3.80 |  |  | 1.13 |
|  | Bite | App | Responder | At least 5% loss | Around 6 | At 6 months |  |  |  |  |  |
| Turner-McGrievy2011[82] | Podcast + mobile | Podcast | Absolute weight loss |  | Around 6 | At 6 months | kg | -0.10 |  |  | 0.66 |
|  | Podcast + mobile | Podcast | Pct loss |  | Around 6 | At 6 months |  | 0.00 |  |  | 1.09 |
| Thomas2020[83] | WW+ES | WW | Absolute weight loss |  | Around 6 | At 6 months | kg | -2.10 |  |  | 1.06 |
|  | WW+ES | WW | Pct loss |  | Around 6 | At 6 months |  | -2.40 |  |  | 1.21 |
|  | WW+ES | WW | Responder | At least 5% loss | Around 6 | At 6 months |  |  |  |  |  |
| Thomas2017[84] | WWO+AL | Control | Absolute weight loss |  | Around 6 |  | kg | -0.20 |  |  | 1.28 |
|  | WWO | Control | Absolute weight loss |  | Around 6 |  | kg | -1.80 |  |  | 1.27 |
|  | WWO+AL | WWO | Absolute weight loss |  | Around 6 |  | kg | 1.60 |  |  | 1.27 |
|  | WWO+AL | Control | Absolute weight loss |  | Around 12 |  | kg | -0.40 |  |  | 0.74 |
|  | WWO | Control | Absolute weight loss |  | Around 12 |  | kg | -0.90 |  |  | 0.72 |
|  | WWO+AL | WWO | Absolute weight loss |  | Around 12 |  | kg | 0.50 |  |  | 0.71 |
|  | WWO+AL | Control | Responder | At least 5% loss | Around 12 |  |  |  |  |  |  |
|  | WWO | Control | Responder | At least 5% loss | Around 12 |  |  |  |  |  |  |
|  | WWO+AL | WWO | Responder | At least 5% loss | Around 12 |  |  |  |  |  |  |
| MD, mean difference; LCI, lower confidence interval; UCI, upper confidence interval; SE, standard error | | | | | | | | | | | |

Bibliography

1. Apinaniz A, Cobos-Campos R, Saez de Lafuente-Morinigo A, Parraza N, Aizpuru F, Perez I, et al. Effectiveness of randomized controlled trial of a mobile app to promote healthy lifestyle in obese and overweight patients. Fam Pract. 2019 Nov 18;36(6):699-705. PMID: 31093681. doi: 10.1093/fampra/cmz020.

2. Backman DR, Kohatsu ND, Padovani AJ, Dao C, Ritley D, Fleuret JE, et al. Achieving weight loss through a community-based, telewellness programme: A randomised controlled trial. Health Educ J. 2022;82(1):82-94. PMID: 2020396037. doi: 10.1177/00178969221139234.

3. Beleigoli A, Andrade AQ, Diniz MF, Ribeiro AL. Personalized Web-Based Weight Loss Behavior Change Program With and Without Dietitian Online Coaching for Adults With Overweight and Obesity: Randomized Controlled Trial. J Med Internet Res. 2020 Nov 5;22(11):e17494. PMID: 33151151. doi: 10.2196/17494.

4. Berli C, Scholz U. Long-Term and Transfer Effects of an Action Control Intervention in Overweight Couples: A Randomized Controlled Trial Using Text Messages. Front Psychol. 2021;12:754488. PMID: 34899496. doi: 10.3389/fpsyg.2021.754488.

5. Braun TD, Olson K, Panza E, Lillis J, Schumacher L, Abrantes AM, et al. Internalized weight stigma in women with class III obesity: A randomized controlled trial of a virtual lifestyle modification intervention followed by a mindful self-compassion intervention. Obes Sci Pract. 2022 Dec;8(6):816-27. PMID: 36483124. doi: 10.1002/osp4.616.

6. Burke LE, Conroy MB, Sereika SM, Elci OU, Styn MA, Acharya SD, et al. The effect of electronic self-monitoring on weight loss and dietary intake: a randomized behavioral weight loss trial. Obesity (Silver Spring). 2011 Feb;19(2):338-44. PMID: 20847736. doi: 10.1038/oby.2010.208.

7. Burke LE, Styn MA, Sereika SM, Conroy MB, Ye L, Glanz K, et al. Using mHealth technology to enhance self-monitoring for weight loss: a randomized trial. Am J Prev Med. 2012 Jul;43(1):20-6. PMID: 22704741. doi: 10.1016/j.amepre.2012.03.016.

8. Turk MW, Elci OU, Wang J, Sereika SM, Ewing LJ, Acharya SD, et al. Self-monitoring as a mediator of weight loss in the SMART randomized clinical trial. Int J Behav Med. 2013 Dec;20(4):556-61. PMID: 22936524. doi: 10.1007/s12529-012-9259-9.

9. Conroy MB, Yang K, Elci OU, Gabriel KP, Styn MA, Wang J, et al. Physical activity self-monitoring and weight loss: 6-month results of the SMART trial. Med Sci Sports Exerc. 2011 Aug;43(8):1568-74. PMID: 21200337. doi: 10.1249/MSS.0b013e31820b9395.

10. Burke LE, Sereika SM, Bizhanova Z, Parmanto B, Kariuki J, Cheng J, et al. The Effect of Tailored, Daily, Smartphone Feedback to Lifestyle Self-Monitoring on Weight Loss at 12 Months: the SMARTER Randomized Clinical Trial. J Med Internet Res. 2022 Jul 5;24(7):e38243. PMID: 35787516. doi: 10.2196/38243.

11. Burke LE, Sereika SM, Parmanto B, Bizhanova Z, Kariuki JK, Cheng J, et al. Effect of tailored, daily feedback with lifestyle self-monitoring on weight loss: The SMARTER randomized clinical trial. Obesity (Silver Spring). 2022 Jan;30(1):75-84. PMID: 34898011. doi: 10.1002/oby.23321.

12. Carter MC, Burley VJ, Nykjaer C, Cade JE. Adherence to a smartphone application for weight loss compared to website and paper diary: pilot randomized controlled trial. J Med Internet Res. 2013 Apr 15;15(4):e32. PMID: 23587561. doi: 10.2196/jmir.2283.

13. Collins CE, Morgan PJ, Hutchesson MJ, Callister R. Efficacy of standard versus enhanced features in a Web-based commercial weight-loss program for obese adults, part 2: randomized controlled trial. J Med Internet Res. 2013 Jul 22;15(7):e140. PMID: 23876832. doi: 10.2196/jmir.2626.

14. Collins CE, Morgan PJ, Hutchesson MJ, Oldmeadow C, Barker D, Callister R. Efficacy of Web-Based Weight Loss Maintenance Programs: A Randomized Controlled Trial Comparing Standard Features Versus the Addition of Enhanced Personalized Feedback over 12 Months. Behav Sci (Basel). 2017 Nov 8;7(4). PMID: 29117105. doi: 10.3390/bs7040076.

15. Conroy MB, McTigue KM, Bryce CL, Tudorascu D, Gibbs BB, Arnold J, et al. Effect of Electronic Health Record-Based Coaching on Weight Maintenance: A Randomized Trial. Ann Intern Med. 2019 Dec 3;171(11):777-84. PMID: 31711168. doi: 10.7326/M18-3337.

16. Duncan MJ, Fenton S, Brown WJ, Collins CE, Glozier N, Kolt GS, et al. Efficacy of a Multi-component m-Health Weight-loss Intervention in Overweight and Obese Adults: A Randomised Controlled Trial. Int J Environ Res Public Health. 2020 Aug 26;17(17). PMID: 32859100. doi: 10.3390/ijerph17176200.

17. Eisenhauer CM, Brito F, Kupzyk K, Yoder A, Almeida F, Beller RJ, et al. Mobile health assisted self-monitoring is acceptable for supporting weight loss in rural men: a pragmatic randomized controlled feasibility trial. BMC Public Health. 2021 Aug 18;21(1):1568. PMID: 34407782. doi: 10.1186/s12889-021-11618-7.

18. Gemesi K, Winkler S, Schmidt-Tesch S, Schederecker F, Hauner H, Holzapfel C. Efficacy of an app-based multimodal lifestyle intervention on body weight in persons with obesity: results from a randomized controlled trial. Int J Obes (Lond). 2024 Jan;48(1):118-26. PMID: 38017117. doi: 10.1038/s41366-023-01415-0.

19. Gold BC, Burke S, Pintauro S, Buzzell P, Harvey-Berino J. Weight loss on the web: A pilot study comparing a structured behavioral intervention to a commercial program. Obesity (Silver Spring). 2007 Jan;15(1):155-64. PMID: 17228043. doi: 10.1038/oby.2007.520.

20. Haapala I, Barengo NC, Biggs S, Surakka L, Manninen P. Weight loss by mobile phone: a 1-year effectiveness study. Public Health Nutr. 2009 Dec;12(12):2382-91. PMID: 19323865. doi: 10.1017/S1368980009005230.

21. Hageman PA, Pullen CH, Hertzog M, Pozehl B, Eisenhauer C, Boeckner LS. Web-Based Interventions Alone or Supplemented with Peer-Led Support or Professional Email Counseling for Weight Loss and Weight Maintenance in Women from Rural Communities: Results of a Clinical Trial. J Obes. 2017;2017:1602627. PMID: 28480078. doi: 10.1155/2017/1602627.

22. Hesseldal L, Christensen JR, Olesen TB, Olsen MH, Jakobsen PR, Laursen DH, et al. Long-term Weight Loss in a Primary Care-Anchored eHealth Lifestyle Coaching Program: Randomized Controlled Trial. J Med Internet Res. 2022 Sep 23;24(9):e39741. PMID: 36149735. doi: 10.2196/39741.

23. Christensen JR, Hesseldal L, Olesen TB, Olsen MH, Jakobsen PR, Laursen DH, et al. Long-term weight loss in a 24-month primary care-anchored telehealth lifestyle coaching program: Randomized controlled trial. J Telemed Telecare. 2022 Dec;28(10):764-70. PMID: 36346936. doi: 10.1177/1357633X221123411.

24. Hutchesson MJ, Callister R, Morgan PJ, Pranata I, Clarke ED, Skinner G, et al. A Targeted and Tailored eHealth Weight Loss Program for Young Women: The Be Positive Be Healthe Randomized Controlled Trial. Healthcare (Basel). 2018 May 2;6(2). PMID: 29724054. doi: 10.3390/healthcare6020039.

25. Jane M, Hagger M, Foster J, Ho S, Kane R, Pal S. Effects of a weight management program delivered by social media on weight and metabolic syndrome risk factors in overweight and obese adults: A randomised controlled trial. PLoS One. 2017;12(6):e0178326. PMID: 28575048. doi: 10.1371/journal.pone.0178326.

26. Jane M, Foster J, Hagger M, Ho S, Kane R, Pal S. Psychological effects of belonging to a Facebook weight management group in overweight and obese adults: Results of a randomised controlled trial. Health Soc Care Community. 2018 May 18. PMID: 29774616. doi: 10.1111/hsc.12584.

27. Dunn CG, Turner-McGrievy GM, Wilcox S, Hutto B. Dietary Self-Monitoring Through Calorie Tracking but Not Through a Digital Photography App Is Associated with Significant Weight Loss: The 2SMART Pilot Study-A 6-Month Randomized Trial. J Acad Nutr Diet. 2019 Sep;119(9):1525-32. PMID: 31155474. doi: 10.1016/j.jand.2019.03.013.

28. Falkenhain K, Locke SR, Lowe DA, Reitsma NJ, Lee T, Singer J, et al. Keyto app and device versus WW app on weight loss and metabolic risk in adults with overweight or obesity: A randomized trial. Obesity (Silver Spring). 2021 Oct;29(10):1606-14. PMID: 34124856. doi: 10.1002/oby.23242.

29. Joseph RP, Todd M, Ainsworth BE, Vega-Lopez S, Adams MA, Hollingshead K, et al. Smart Walk: A Culturally Tailored Smartphone-Delivered Physical Activity Intervention for Cardiometabolic Risk Reduction among African American Women. Int J Environ Res Public Health. 2023 Jan 5;20(2). PMID: 36673756. doi: 10.3390/ijerph20021000.

30. Kempf K, Rohling M, Martin S, Schneider M. Telemedical coaching for weight loss in overweight employees: a three-armed randomised controlled trial. BMJ Open. 2019 Apr 11;9(4):e022242. PMID: 30975666. doi: 10.1136/bmjopen-2018-022242.

31. Kempf K, Rohling M, Stichert M, Fischer G, Boschem E, Konner J, et al. Telemedical Coaching Improves Long-Term Weight Loss in Overweight Persons: A Randomized Controlled Trial. Int J Telemed Appl. 2018;2018:7530602. PMID: 30271433. doi: 10.1155/2018/7530602.

32. Keshavarz M, Senechal M, Bouchard DR. Online Circuit Training Increases Adherence to Physical Activity: A Randomized Controlled Trial of Men with Obesity. Med Sci Sports Exerc. 2023 Dec 1;55(12):2308-15. PMID: 37535330. doi: 10.1249/MSS.0000000000003270.

33. Kharmats AY, Wang C, Fuentes L, Hu L, Kline T, Welding K, et al. Monday-focused tailored rapid interactive mobile messaging for weight management 2 (MTRIMM2): results from a randomized controlled trial. Mhealth. 2022;8:1. PMID: 35178432. doi: 10.21037/mhealth-21-3.

34. Kim M, Kim Y, Go Y, Lee S, Na M, Lee Y, et al. Multidimensional Cognitive Behavioral Therapy for Obesity Applied by Psychologists Using a Digital Platform: Open-Label Randomized Controlled Trial. JMIR Mhealth Uhealth. 2020 Apr 30;8(4):e14817. PMID: 32352391. doi: 10.2196/14817.

35. Kohl J, Brame J, Centner C, Wurst R, Fuchs R, Sehlbrede M, et al. Effects of a Web-Based Lifestyle Intervention on Weight Loss and Cardiometabolic Risk Factors in Adults With Overweight and Obesity: Randomized Controlled Clinical Trial. J Med Internet Res. 2023 Jun 27;25:e43426. PMID: 37368484. doi: 10.2196/43426.

36. Kurtzman GW, Day SC, Small DS, Lynch M, Zhu J, Wang W, et al. Social Incentives and Gamification to Promote Weight Loss: The LOSE IT Randomized, Controlled Trial. J Gen Intern Med. 2018 Oct;33(10):1669-75. PMID: 30003481. doi: 10.1007/s11606-018-4552-1.

37. Laing BY, Mangione CM, Tseng CH, Leng M, Vaisberg E, Mahida M, et al. Effectiveness of a smartphone application for weight loss compared with usual care in overweight primary care patients: a randomized, controlled trial. Ann Intern Med. 2014 Nov 18;161(10 Suppl):S5-12. PMID: 25402403. doi: 10.7326/M13-3005.

38. Bennett GG, Foley P, Levine E, Whiteley J, Askew S, Steinberg DM, et al. Behavioral treatment for weight gain prevention among black women in primary care practice: a randomized clinical trial. JAMA Intern Med. 2013 Oct 28;173(19):1770-7. PMID: 23979005. doi: 10.1001/jamainternmed.2013.9263.

39. Lanpher MG, Askew S, Bennett GG. Health Literacy and Weight Change in a Digital Health Intervention for Women: A Randomized Controlled Trial in Primary Care Practice. J Health Commun. 2016;21 Suppl 1(Suppl):34-42. PMID: 27043756. doi: 10.1080/10810730.2015.1131773.

40. LaRose JG, Leahey TM, Lanoye A, Bean MK, Fava JL, Tate DF, et al. Effect of a Lifestyle Intervention on Cardiometabolic Health Among Emerging Adults: A Randomized Clinical Trial. JAMA Netw Open. 2022 Sep 1;5(9):e2231903. PMID: 36121656. doi: 10.1001/jamanetworkopen.2022.31903.

41. Leahey TM, Subak LL, Fava J, Schembri M, Thomas G, Xu X, et al. Benefits of adding small financial incentives or optional group meetings to a web-based statewide obesity initiative. Obesity (Silver Spring). 2015 Jan;23(1):70-6. PMID: 25384463. doi: 10.1002/oby.20937.

42. Leahey TM, Fava JL, Seiden A, Fernandes D, Doyle C, Kent K, et al. A randomized controlled trial testing an Internet delivered cost-benefit approach to weight loss maintenance. Prev Med. 2016 Nov;92:51-7. PMID: 27095323. doi: 10.1016/j.ypmed.2016.04.013.

43. Little P, Stuart B, Hobbs FR, Kelly J, Smith ER, Bradbury KJ, et al. An internet-based intervention with brief nurse support to manage obesity in primary care (POWeR+): a pragmatic, parallel-group, randomised controlled trial. Lancet Diabetes Endocrinol. 2016 Oct;4(10):821-8. PMID: 27474214. doi: 10.1016/S2213-8587(16)30099-7.

44. Little P, Stuart B, Hobbs FR, Kelly J, Smith ER, Bradbury KJ, et al. Randomised controlled trial and economic analysis of an internet-based weight management programme: POWeR+ (Positive Online Weight Reduction). Health Technol Assess. 2017 Jan;21(4):1-62. PMID: 28122658. doi: 10.3310/hta21040.

45. Markkanen JO, Oikarinen N, Savolainen MJ, Merikallio H, Nyman V, Salminen V, et al. Mobile health behaviour change support system as independent treatment tool for obesity: a randomized controlled trial. Int J Obes (Lond). 2024 Mar;48(3):376-83. PMID: 38062218. doi: 10.1038/s41366-023-01426-x.

46. McConnon A, Kirk SF, Cockroft JE, Harvey EL, Greenwood DC, Thomas JD, et al. The Internet for weight control in an obese sample: results of a randomised controlled trial. BMC Health Serv Res. 2007 Dec 19;7:206. PMID: 18093289. doi: 10.1186/1472-6963-7-206.

47. Morgan PJ, Lubans DR, Collins CE, Warren JM, Callister R. The SHED-IT randomized controlled trial: evaluation of an Internet-based weight-loss program for men. Obesity (Silver Spring). 2009 Nov;17(11):2025-32. PMID: 19343018. doi: 10.1038/oby.2009.85.

48. Morgan PJ, Collins CE, Plotnikoff RC, Cook AT, Berthon B, Mitchell S, et al. Efficacy of a workplace-based weight loss program for overweight male shift workers: the Workplace POWER (Preventing Obesity Without Eating like a Rabbit) randomized controlled trial. Prev Med. 2011 May;52(5):317-25. PMID: 21300083. doi: 10.1016/j.ypmed.2011.01.031.

49. Morgan PJ, Callister R, Collins CE, Plotnikoff RC, Young MD, Berry N, et al. The SHED-IT community trial: a randomized controlled trial of internet- and paper-based weight loss programs tailored for overweight and obese men. Ann Behav Med. 2013 Apr;45(2):139-52. PMID: 23129021. doi: 10.1007/s12160-012-9424-z.

50. Blomfield RL, Collins CE, Hutchesson MJ, Young MD, Jensen ME, Callister R, et al. Impact of self-help weight loss resources with or without online support on the dietary intake of overweight and obese men: the SHED-IT randomised controlled trial. Obes Res Clin Pract. 2014 Sep-Oct;8(5):e476-87. PMID: 25263837. doi: 10.1016/j.orcp.2013.09.004.

51. Mueller J, Richards R, Jones RA, Whittle F, Woolston J, Stubbings M, et al. Supporting Weight Management during COVID-19: A Randomized Controlled Trial of a Web-Based, ACT-Based, Guided Self-Help Intervention. Obes Facts. 2022;15(4):550-9. PMID: 35417910. doi: 10.1159/000524031.

52. Mueller J, Richards R, Jones RA, Whittle F, Woolston J, Stubbings M, et al. Supporting Weight Management during COVID-19 (SWiM-C): twelve-month follow-up of a randomised controlled trial of a web-based, ACT-based, guided self-help intervention. Int J Obes (Lond). 2023 Jan;47(1):51-9. PMID: 36369513. doi: 10.1038/s41366-022-01232-x.

53. Patel ML, Hopkins CM, Brooks TL, Bennett GG. Comparing Self-Monitoring Strategies for Weight Loss in a Smartphone App: Randomized Controlled Trial. JMIR Mhealth Uhealth. 2019 Feb 28;7(2):e12209. PMID: 30816851. doi: 10.2196/12209.

54. Patrick K, Calfas KJ, Norman GJ, Rosenberg D, Zabinski MF, Sallis JF, et al. Outcomes of a 12-month web-based intervention for overweight and obese men. Ann Behav Med. 2011 Dec;42(3):391-401. PMID: 21822750. doi: 10.1007/s12160-011-9296-7.

55. Rogers RJ, Lang W, Barone Gibbs B, Davis KK, Burke LE, Kovacs SJ, et al. Applying a technology-based system for weight loss in adults with obesity. Obes Sci Pract. 2016 Mar;2(1):3-12. PMID: 27812375. doi: 10.1002/osp4.18.

56. Ross KM, Wing RR. Impact of newer self-monitoring technology and brief phone-based intervention on weight loss: A randomized pilot study. Obesity (Silver Spring). 2016 Aug;24(8):1653-9. PMID: 27367614. doi: 10.1002/oby.21536.

57. Roth L, Ordnung M, Forkmann K, Mehl N, Horstmann A. A randomized-controlled trial to evaluate the app-based multimodal weight loss program zanadio for patients with obesity. Obesity (Silver Spring). 2023 May;31(5):1300-10. PMID: 37140392. doi: 10.1002/oby.23744.

58. Shapiro JR, Koro T, Doran N, Thompson S, Sallis JF, Calfas K, et al. Text4Diet: a randomized controlled study using text messaging for weight loss behaviors. Prev Med. 2012 Nov;55(5):412-7. PMID: 22944150. doi: 10.1016/j.ypmed.2012.08.011.

59. Shuger SL, Barry VW, Sui X, McClain A, Hand GA, Wilcox S, et al. Electronic feedback in a diet- and physical activity-based lifestyle intervention for weight loss: a randomized controlled trial. Int J Behav Nutr Phys Act. 2011 May 18;8:41. PMID: 21592351. doi: 10.1186/1479-5868-8-41.

60. Silina V, Tessma MK, Senkane S, Krievina G, Bahs G. Text messaging (SMS) as a tool to facilitate weight loss and prevent metabolic deterioration in clinically healthy overweight and obese subjects: a randomised controlled trial. Scand J Prim Health Care. 2017 Sep;35(3):262-70. PMID: 28812403. doi: 10.1080/02813432.2017.1358435.

61. Simpson SA, Matthews L, Pugmire J, McConnachie A, McIntosh E, Coulman E, et al. An app-, web- and social support-based weight loss intervention for adults with obesity: the HelpMeDoIt! feasibility RCT. Public Health Research. 2020;8(3). doi: <https://dx.doi.org/10.3310/phr08030>.

62. Simpson SA, Matthews L, Pugmire J, McConnachie A, McIntosh E, Coulman E, et al. An app-, web- and social support-based weight loss intervention for adults with obesity: the 'HelpMeDoIt!' feasibility randomised controlled trial. Pilot Feasibility Stud. 2020;6:133. PMID: 32968544. doi: 10.1186/s40814-020-00656-4.

63. Sniehotta FF, Evans EH, Sainsbury K, Adamson A, Batterham A, Becker F, et al. Behavioural intervention for weight loss maintenance versus standard weight advice in adults with obesity: A randomised controlled trial in the UK (NULevel Trial). PLoS Med. 2019 May;16(5):e1002793. PMID: 31063507. doi: 10.1371/journal.pmed.1002793.

64. Steinberg DM, Levine EL, Askew S, Foley P, Bennett GG. Daily text messaging for weight control among racial and ethnic minority women: randomized controlled pilot study. J Med Internet Res. 2013 Nov 18;15(11):e244. PMID: 24246427. doi: 10.2196/jmir.2844.

65. Tate DF, Wing RR, Winett RA. Using Internet technology to deliver a behavioral weight loss program. JAMA. 2001 Mar 7;285(9):1172-7. PMID: 11231746. doi: 10.1001/jama.285.9.1172.

66. Tate DF, Jackvony EH, Wing RR. A randomized trial comparing human e-mail counseling, computer-automated tailored counseling, and no counseling in an Internet weight loss program. Arch Intern Med. 2006;166(15):1620-5.

67. Tate DF, Lutes LD, Bryant M, Truesdale KP, Hatley KE, Griffiths Z, et al. Efficacy of a Commercial Weight Management Program Compared With a Do-It-Yourself Approach: A Randomized Clinical Trial. JAMA Netw Open. 2022 Aug 1;5(8):e2226561. PMID: 35972742. doi: 10.1001/jamanetworkopen.2022.26561.

68. Teeriniemi AM, Salonurmi T, Jokelainen T, Vahanikkila H, Alahaivala T, Karppinen P, et al. A randomized clinical trial of the effectiveness of a Web-based health behaviour change support system and group lifestyle counselling on body weight loss in overweight and obese subjects: 2-year outcomes. J Intern Med. 2018 Nov;284(5):534-45. PMID: 29974563. doi: 10.1111/joim.12802.

69. Dombrowski SU, McDonald M, van der Pol M, Grindle M, Avenell A, Carroll P, et al. Game of Stones: feasibility randomised controlled trial of how to engage men with obesity in text message and incentive interventions for weight loss. BMJ Open. 2020 Feb 25;10(2):e032653. PMID: 32102807. doi: 10.1136/bmjopen-2019-032653.

70. Dombrowski SU, McDonald M, van der Pol M, Grindle M, Avenell A, Carroll P, et al. Text messaging and financial incentives to encourage weight loss in men with obesity: the Game of Stones feasibility RCT. Public Health Research 2020;8(11). doi: <https://dx.doi.org/10.3310/phr08110>.

71. Young MD, Callister R, Collins CE, Plotnikoff RC, Aguiar EJ, Morgan PJ. Efficacy of a gender-tailored intervention to prevent weight regain in men over 3 years: A weight loss maintenance RCT. Obesity (Silver Spring). 2017 Jan;25(1):56-65. PMID: 27925437. doi: 10.1002/oby.21696.

72. Womble LG, Wadden TA, McGuckin BG, Sargent SL, Rothman RA, Krauthamer-Ewing ES. A randomized controlled trial of a commercial internet weight loss program. Obes Res. 2004 Jun;12(6):1011-8. PMID: 15229342. doi: 10.1038/oby.2004.124.

73. Olson R, Wipfli B, Thompson SV, Elliot DL, Anger WK, Bodner T, et al. Weight Control Intervention for Truck Drivers: The SHIFT Randomized Controlled Trial, United States. Am J Public Health. 2016 Sep;106(9):1698-706. PMID: 27463067. doi: 10.2105/AJPH.2016.303262.

74. Wipfli B, Hanson G, Anger K, Elliot DL, Bodner T, Stevens V, et al. Process Evaluation of a Mobile Weight Loss Intervention for Truck Drivers. Saf Health Work. 2019 Mar;10(1):95-102. PMID: 30949387. doi: 10.1016/j.shaw.2018.08.002.

75. West DS, Stansbury M, Krukowski RA, Harvey J. Enhancing group-based internet obesity treatment: A pilot RCT comparing video and text-based chat. Obes Sci Pract. 2019 Dec;5(6):513-20. PMID: 31890241. doi: 10.1002/osp4.371.

76. West DS, Harvey JR, Krukowski RA, Prewitt TE, Priest J, Ashikaga T. Do individual, online motivational interviewing chat sessions enhance weight loss in a group-based, online weight control program? Obesity (Silver Spring). 2016 Nov;24(11):2334-40. PMID: 27616628. doi: 10.1002/oby.21645.

77. van Wier MF, Ariens GA, Dekkers JC, Hendriksen IJ, Smid T, van Mechelen W. Phone and e-mail counselling are effective for weight management in an overweight working population: a randomized controlled trial. BMC Public Health. 2009 Jan 9;9:6. PMID: 19134171. doi: 10.1186/1471-2458-9-6.

78. van Wier MF, Dekkers JC, Hendriksen IJ, Heymans MW, Ariens GA, Pronk NP, et al. Effectiveness of phone and e-mail lifestyle counseling for long term weight control among overweight employees. J Occup Environ Med. 2011 Jun;53(6):680-6. PMID: 21654441. doi: 10.1097/JOM.0b013e31821f2bbb.

79. van Wier MF, Dekkers JC, Bosmans JE, Heymans MW, Hendriksen IJ, Pronk NP, et al. Economic evaluation of a weight control program with e-mail and telephone counseling among overweight employees: a randomized controlled trial. Int J Behav Nutr Phys Act. 2012 Sep 11;9:112. PMID: 22967224. doi: 10.1186/1479-5868-9-112.

80. van Genugten L, van Empelen P, Boon B, Borsboom G, Visscher T, Oenema A. Results from an online computer-tailored weight management intervention for overweight adults: randomized controlled trial. J Med Internet Res. 2012 Mar 14;14(2):e44. PMID: 22417813. doi: 10.2196/jmir.1901.

81. Turner-McGrievy GM, Wilcox S, Boutte A, Hutto BE, Singletary C, Muth ER, et al. The Dietary Intervention to Enhance Tracking with Mobile Devices (DIET Mobile) Study: A 6-Month Randomized Weight Loss Trial. Obesity (Silver Spring). 2017 Aug;25(8):1336-42. PMID: 28600833. doi: 10.1002/oby.21889.

82. Turner-McGrievy G, Tate D. Tweets, Apps, and Pods: Results of the 6-month Mobile Pounds Off Digitally (Mobile POD) randomized weight-loss intervention among adults. J Med Internet Res. 2011 Dec 20;13(4):e120. PMID: 22186428. doi: 10.2196/jmir.1841.

83. Thomas JG, Goldstein CM, Bond DS, Hadley W, Tuerk PW. Web-based virtual reality to enhance behavioural skills training and weight loss in a commercial online weight management programme: The Experience Success randomized trial. Obes Sci Pract. 2020 Dec;6(6):587-95. PMID: 33354337. doi: 10.1002/osp4.451.

84. Thomas JG, Raynor HA, Bond DS, Luke AK, Cardoso CC, Foster GD, et al. Weight loss in Weight Watchers Online with and without an activity tracking device compared to control: A randomized trial. Obesity (Silver Spring). 2017 Jun;25(6):1014-21. PMID: 28437597. doi: 10.1002/oby.21846.
