# Supplementary material for: Evaluation of the Aspects of Digital Interventions That Successfully Support Weight Loss: Systematic Review With Component Network Meta-Analysis
Source: J Med Internet Res. 2025 May 22;27:e65443. doi: 10.2196/65443 (PMC12141966; doi:10.2196/65443)
Supplement: Multimedia Appendix 8 [file jmir_v27i1e65443_app8.docx]

### Multimedia Appendix 8. Results of component network meta-analysis for percentage weight loss.


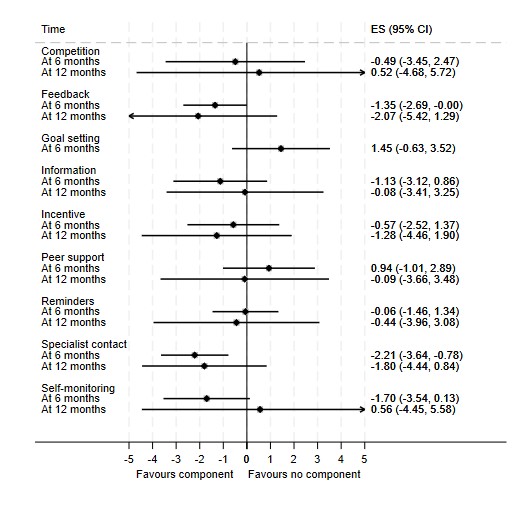


Component network meta-analysis of digital components of interventions, for percent weight loss at 6 and 12 months. ES, effect size; 95%CI, 95% confidence interval
